# Delineating the Effects of Counterions on the Structural and Vibrational Properties of U(IV) Lindqvist Polyoxometalate Complexes

**DOI:** 10.1021/acs.inorgchem.5c00033

**Published:** 2025-05-30

**Authors:** Primadi J. Subintoro, Korey P. Carter

**Affiliations:** Department of Chemistry, 4083 University of Iowa, Iowa City, Iowa 52242, United States

## Abstract

Herein we conducted a full investigation into the fundamental structural and vibrational properties of uranium­(IV) Peacock–Weakley-type lacunary Lindqvist (W_10_) polyoxometalate (POM) complexes. We recently demonstrated the importance of the secondary lattice elements in tuning the distortion of the D_4d_ symmetry in W_10_ POM complexes, and here, we synthesized eight UW_10_ complexes with different alkali metal counterions and evaluated how the composition and packing of counterion species affected complex structural and vibrational properties. Single-crystal X-ray diffraction analysis on complexes **1–8** revealed changes in structural distortion parameters as a function of differences in counterion configurations, while far-infrared and Raman spectra for **1**–**8** also demonstrated that vibrational mode frequencies were sensitive to changes in counterion composition and packing. To more effectively compare different counterion configurations, we developed counterion effective ionic radius (eIR) as a new structural parameter, and comparisons between structural distortion parameters and eIR values strongly suggested that modulation by the secondary lattice elements can affect structural and vibrational manifolds within POM complexes. Partial least squares (PLS) analysis was used to quantitatively evaluate correlations observed within this investigation, and PLS statistical models showed a strong correlation between counterion eIR and both structural distortion parameters and vibrational mode frequencies.

## Graphical abstract

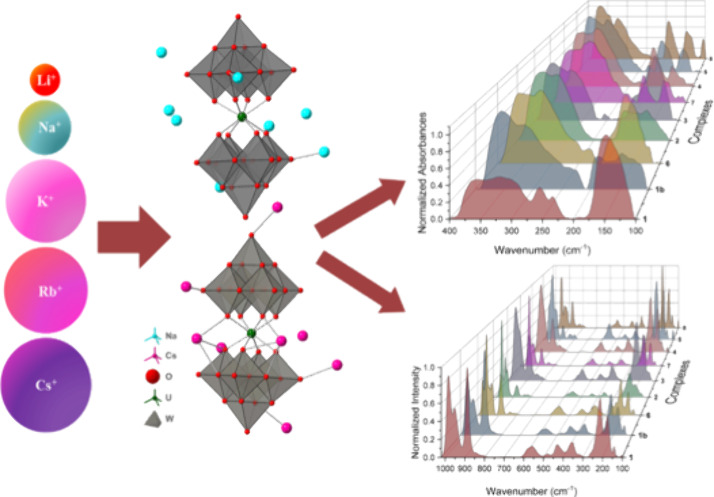

## Introduction

Polyoxometalates (POMs) are metallic oxide materials formed by MO_6_ octahedral moieties of primarily group V and VI metals (V, Nb, Ta, Mo, and W) that are connected through bridging oxygen atoms to form discrete structures.^1,2^ This class of materials is continuously relevant due to their topological diversity and modularity, which allow for unique electronic properties to be realized both in the POM and for the metals that POMs encapsulate.^3,4^ Evidence of the potential of POMs can be seen through the wide range of applications where POMs have been used, including catalysis, medical contrast agents, and, more recently, quantum information science (QIS).^5–9^ POMs are known to form stable complexes with highly oxophilic cations such as f-block elements, and this is especially true for lacunary POMs wherein vacancies in MO_6_ units are generated, thereby increasing the basicity of the remaining bridging oxygen atoms.^2^ The diverse array of known lacunary POM species provides a pathway to generate f-element coordination complexes with a range of unique topologies,^10^ and notable examples with lanthanide cations include Peacock–Weakley-type sandwich complexes with lacunary Lindqvist, Keggin, and Wells–Dawson POMs.^11–15^ More recently, lacunary POMs have also been documented to successfully chelate microgram quantities of Am­(III) and Cm­(III), which highlights the translatability of POM chemistry toward the actinides.^16–18^ Beyond providing a platform to study metal–ligand bonding with rare transplutonium elements, POM chemistry has also been investigated with early actinides including thorium, uranium, and neptunium.^19–23^

Actinide POM chemistry has been an area of sustained research interest since the 1970s as this class of complexes was initially investigated for the separation and storage of radioactive elements,^24^ and current efforts have evolved to take advantage of the rich redox and spin-based properties exhibited by actinide complexes.^25,26^ The advent of high-performance molecular magnets based on Ln­(III) cations, such as Dy­(III) and Tb­(III), is one new avenue for exploration, especially as theoretical calculations on uranium­(III) and U­(V) molecular magnets suggest that these systems should be able to eclipse the performance of their lanthanide counterparts, although experimental results have thus far shown otherwise.^25,27,28^ The challenge in realizing actinide single molecule magnets is not a limiting factor for other applications that can take advantage of the unique spin-based properties of the 5f elements, such as QIS where actinide complexes could function as electron spin quantum bits (qubits). Molecular spintronic materials, especially spin qubits, are often paramagnetic metal complexes wherein a two-level system can be generated via application of a magnetic field.^29–31^ A key metric for assessing spin qubits is the coherence time of a species, which is a measure of the length of time spins spend in the superposition state of the two spin-levels. Baldovi et al. have predicted that a U­(IV) molecule with a stabilized M_J_ = ±4 ground state could feature a large tunnel splitting gap akin to the one exhibited by the model spin qubit, Na_9_Ho­(W_5_O_18_)_2_·35H_2_O (HoW_10_),^32,33^ and realization of this property in a U­(IV) POM could lead to an extremely long coherence time via an accessible atomic clock transition. Inspired by the work of Shiddiq and colleagues, who detailed that HoW_10_ could act as a spin qubit, as well as our recent study that elucidated the role of structural polymorphism and second sphere interactions on vibrational properties of LnW_10_ complexes,^15,33^ we have extended our POM chemistry investigations to the [U­(IV)­(W_5_O_18_)_2_]^8–^ (UW_10_) system. The distorted square antiprismatic (D_4d_) symmetry that results from the formation of a sandwich complex with lacunary Lindqvist POMs stabilizes the ±4 M_J_ states of the Ho­(III) cation in HoW_10_, and our hypothesis here is that the same outcome can be achieved for the non-Kramers U­(IV) ion, which also possesses a M_J_ = ±4 ground state.

Herein we investigated the structural and vibrational properties of UW_10_ complexes with each of the alkali metals acting as charge balancing counterions. Na_8_[UW_10_] was first discovered by Ripan and Botar in 1970;^34^ however, they misidentified the complex as U­(IV) octatungstate, and it was not until 1975 when Golubev et al. correctly described the structure.^35^ Despite its fundamental importance in actinide POM chemistry, there has not been a study that elucidates the fundamental properties of UW_10_ complexes since the study from Golubev and colleagues five decades ago.^35^ In our recent work focusing on Na_9_Ln­(W_5_O_18_)_2_·*X*H_2_O complexes, we found that the secondary lattice influenced the effective symmetry of the Ln­(III) center, which affected both the vibrational and spin manifolds of the complexes. Colliard and Deblonde have also noted direct ion pairing interactions with POM clusters and actinide metal centers in their recent studies focused on transplutonium POMs, where Cs­(I) has been used as a counterion.^18^ Here we aimed to develop a more comprehensive understanding of the role of counterions on the second sphere interactions in POM complexes by synthesizing UW_10_ complexes with combinations of Na­(I) and each of the other alkali metals acting as charge balancing cations. Further, we also prepared UW_10_ complexes with only Li­(I), K­(I), or Cs­(I) counterions to investigate whether and to what extent the effective symmetry of a metal center can be tuned by controlling the composition of counterions within the secondary sphere. Overall, eight species of UW_10_ (Li_5_Na_3_[UW_10_] (UW_10_Li, **1**), Na_8_[UW_10_] (UW_10_Na, **2**), K_4_Na_4_[UW_10_] (UW_10_K, **3**), Rb_6_Na_2_[UW_10_] (UW_10_Rb, **4**), Cs_5.5_Na_2.5_[UW_10_] (UW_10_Cs, **5**), Li_8_[UW_10_] (UW_10_LiF, **6**), K_8_[UW_10_] (UW_10_KF, **7**), and Cs_8_[UW_10_] (UW_10_CsF, **8**)) were synthesized and characterized structurally using X-ray diffraction and vibrationally using Raman, mid-infrared (MIR), and far-infrared (FIR) spectroscopies. Structural analyses of complexes **1**–**8** show that larger counterions (K­(I), Rb­(I), and Cs­(I)) participate in more ion-pairing interactions with the cluster compared to smaller counterions (Li­(I) and Na­(I)). Manifestations of these differences in secondary sphere interactions are significant as the effective symmetry of the U­(IV) metal center in each complex is modulated, which we quantifiably probed using partial least-squares (PLS) analysis. Qualitative and PLS analyses of the structural and vibrational properties of complexes **1**–**8** also show correlations between the identity of secondary sphere cations and shifts in Raman and IR stretching frequencies. With this study, we aim to reinvigorate interest in the UW_10_ species as it could be a model complex for actinide QIS applications based on its relevant symmetry for magnetic and spintronic purposes as well as a template for extending POM chemistry to tetravalent transuranic systems.

## Materials and Methods

***Caution!*** ^238^U (*t*_1/2_ = 4.47 × 10^9^ years) is an α-emitting radionuclide that should be manipulated only in a specifically designated facility in accordance with appropriate safety controls. All measurements were taken either in the University of Iowa radiological laboratories or using multiple containment procedures.

### Materials

All chemicals were purchased from commercial vendors and were used as received. This includes uranyl nitrate hexahydrate (UO_2_(NO_3_)_2_·6H_2_O) (International Bioanalytical Industries, 98%), hexachloropropene (C_3_Cl_6_) (Sigma-Aldrich, ≥90%), sodium tungstate dihydrate (Na_2_WO_4_·2H_2_O) (Strem Chemicals, Inc., 99+%), potassium tungstate (K_2_WO_4_) (Beantown Chemical, 99.5%), cesium tungstate (Cs_2_WO_4_) (Beantown Chemical, 99.9%), lithium chloride (LiCl) (Strem Chemicals, Inc., 99%), sodium chloride (NaCl) (Fisher Scientific, 99%), potassium chloride (KCl) (VWR BDH Chemicals, 99%), rubidium chloride (RbCl) (Ambeed Inc., 99%), cesium chloride (CsCl) (TCI America, 99%), sodium hydroxide pellets (Fisher Scientific, ACS grade), and 70% nitric acid (Sigma-Aldrich or Fisher Scientific, ACS reagent).

### Experimental Methods

#### Synthesis of UCl_4_

The method used here for preparing UCl_4_ was adapted from Liddle et al. and Hermann et al.^36,37^ A 1.4779 g portion of uranyl nitrate hexahydrate (UO_2_(NO_3_)_2_·6H_2_O) (∼3 mmol) and 12 mL of hexachloropropene (C_3_Cl_6_) were added into a 50 mL round-bottom flask equipped with a reflux condenser. The suspension was then refluxed at 210 °C for approximately 12 h. The solid UO_2_(NO_3_)_2_·6H_2_O was dissolved in the solution at 180 °C, turning it brown in color. Brown fumes were produced at temperatures above 180 °C followed by white fumes at temperatures above 200 °C. At 210 °C, a dark green precipitate formed, and the brown solution darkened. After 12 h, the solution was cooled and then subsequently transferred to a 15 mL centrifuge tube with a minimal amount (∼5 mL) of dichloromethane (DCM). The solution was centrifuged at 7830 rpm for 2 min, and the supernatant liquid was removed. A dark green pellet remained, and it was washed with DCM three times and dried in a vacuum desiccator for approximately 3 h. The dark green solid was UCl_4_ (yield = 1.1856 g, 106.11%), and a greater than 100% yield was due to residual DCM. This impurity did not impact subsequent POM synthesis, so the UCl_4_ product was dissolved in 20 mL of 2 M hydrochloric acid, and the remaining undissolved solids were removed by centrifugation. The final UCl_4_ concentration was approximately 0.15 M, and this stock solution was stored in a 5 °C fridge to maintain uranium oxidation state stability between reactions.

#### Synthesis of M_8–*x*_Na_*x*_[U­(W_5_O_18_)_2_]·*Y*H_2_O (M = Li (**1**), Na (**2**), K (**3**), Rb (**4**), Cs (**5**)) and M_8_[U­(W_5_O_18_)_2_]·*X*H_2_O (M = Li (**6**))

Synthesis of UW_10_ complexes used a procedure adapted from the work of Mariichak and colleagues.^38^ 20 mL of 0.5 M Na_2_WO_4_·2H_2_O solution (10 mmol) and 0.51 mL of concentrated HNO_3_ (∼8 mmol) were mixed in a 100 mL round-bottom flask with vigorous stirring (pH = 7.4). 6.667 mL of 0.15 M UCl_4_ solution was added into the acidified tungstate solution (pH = 0.10) in 500 μL portions every 30 s. 3.333 mL of 4 M NaOH was then added to readjust the pH to 6.7, and 19.49 mL of Milli-Q H_2_O was added to yield a final volume of 50 mL. This solution was split equally into five vials, and then, 6.667 mL of the respective alkali metal chloride stock solution, either 2 M LiCl, KCl, RbCl, or CsCl (13.332 mmol), was added except for complex **2** where Na­(I) ions in the reaction solution from Na_2_WO_4_·2H_2_O and NaOH provided a sufficient concentration of this counterion. UW_10_ solutions were stirred for 15 min, and then, each was centrifuged for 5 min at 7830 rpm. Supernatant solutions were collected and left to slowly evaporate in plastic Petri dishes inside a 5 °C fridge. During this slow evaporation period, small amounts of white precipitate formed in each reaction, and these precipitates were removed via centrifugation at 5 °C. Supernatant solutions were extracted and left to slowly evaporate again until dark brown crystals formed after approximately 3 days. Worthy of additional comment, complexes **2**–**5** are indefinitely stable upon crystallization, while complex **6** was isolated from the first round of crystallization during the synthesis of complex **1**. Subsequently, complex **1** can be isolated; however, this species also breaks down into complex **2** after several rounds of recrystallization, which is a likely result of the Li­(I) ions being mobile within the lattice due to a lack of association interactions with the UW_10_ POM complex.

#### Synthesis of M_8_[U­(W_5_O_18_)_2_]·*X*H_2_O (M = K (**7**), Cs (**8**))

204 mg of K_2_WO_4_ (0.625 mmol) was dissolved in 5 mL of Milli-Q H_2_O. The pH of the tungstate solution was raised to approximately 12 with 4 M KOH, and then, the tungstate solution was acidified to a pH of 6.5–7 with concentrated HNO_3_. 417 μL of 0.15 M UCl_4_ solution was then added into the tungstate solution. The pH of the solution was readjusted back to between 6.5 and 7 with 4 M KOH solution, and the solution was stirred for 15 min at 60 °C. The solution was then centrifuged, and the supernatant was extracted and left to slowly evaporate until dark brown crystals formed after approximately 1 week. The Cs_8_ version of UW_10_ was synthesized following the same protocol outlined above for the K_8_ complex with 321 mg of Cs_2_WO_4_ (0.625 mmol) and 2 M CsOH used instead of K_2_WO_4_ and KOH.

#### Single-Crystal X-ray Structure Determination

Single-crystal X-ray diffraction data for complexes **1**–**8** were collected at 100(2) K on a Bruker D8 Venture Duo Diffractometer with a Mo X-ray source and Kα_1_ = 0.71073 Å. Adsorption corrections were applied using the SADABS multiscan method within the APEX4 software package.^39,40^ Structures were solved via intrinsic phasing using SHELXT and refined with SHELXL contained within Olex2 1.5.^41,42^ All non-hydrogen atoms were refined anisotropically. Due to disorder, some of the lattice counterions and water oxygen atoms were refined with less than full occupancy. ISOR restraints were also used, when necessary, on lattice atoms to yield reasonable thermal ellipsoids. Complex **4** crystallized in a noncentrosymmetric space group, and the Hooft parameter from the refinement suggested that the data set was collected on a racemically twinned crystal. To account for this twinning, a racemic twin law and BASF commands were applied to the refinement, which substantively improved the crystallographic refinement metrics. Hydrogen atoms on the lattice water molecules that could not be located in difference Fourier maps for complexes **1**–**8** were not modeled. All figures for complexes **1**–**8** were made using CrystalMaker.^43^ The packing of complexes **1**–**8** was determined by centering the structure on the U atoms and expanding the range until all counterions were located. Crystallographic parameters for complexes **1**–**8** are available in Table 1. CIF files available online in the Cambridge Crystallographic Database Center (CCDC) at http://www.ccdc.cam.ac.uk by citing reference numbers 2381891–2381898.

**Table 1:** Crystallographic Parameters for Complexes **1**–**8**

parameter	UW_10_Li (**1**)	UW_10_Na (**2**)	UW_10_K (**3**)	UW_10_Rb (**4**)	UW_10_Cs (**5**)	UW_10_LiF (**6**)	UW_10_KF (**7**)	UW_10_CsF (**8**)
formula	Li_5_Na_3_UW_10_O_36_·19H_2_O	Na_8_UW_10_O_36_·31H_2_O	K_4_Na_4_UW_10_O_36_·17H_2_O	Rb_6_Na_2_UW_10_O_36_·11H_2_O	Cs_5.5_Na_2.5_UW_10_O_36_·5H_2_O	Li_8_UW_10_O_36_·14H_2_O	K_8_UW_10_O_36_·5H_2_O	Cs_8_UW_10_O_36_·12H_2_O
Mr	3220.2	3332.45	3160.89	3387.33	3552.88	2932.05	3149.33	3899.81
SG	*P*2_1_/*n*	*C*2/*c*	*P*2_1_/*c*	*Cc*	*Pnma*	*P*2_1_/*n*	*P*2/*n*	*P*-1
*a* (Å)	18.1042 (6)	18.0046 (8)	25.9688 (14)	9.8894 (4)	27.4711 (12)	9.2004 (3)	21.7357 (7)	10.3539 (3)
*b* (Å)	18.2289 (6)	18.6315 (8)	11.2067 (5)	20.0696 (9)	11.3562 (4)	29.5003 (8)	9.4184 (3)	13.1902 (5)
*c* (Å)	18.2154 (6)	18.4017 (7)	17.58058 (9)	26.0571 (9)	17.0364 (7)	15.2620 (6)	25.5666 (9)	19.2245 (7)
α	90	90	90	90	90	90	90	88.171 (2)
β	98.962 (1)	95.213 (2)	92.994 (2)	93.641 (2)	90	93.980 (2)	103.065 (1)	85.149 (1)
γ	90	90	90	90	90	90	90	75.357 (1)
*V* (Å^3^)	5938.1 (3)	6147.4 (4)	5109.4 (4)	5161.3 (4)	5314.8(4)	4132.3 (2)	5098.4 (3)	2530.96 (15)
*R*int	0.0952	0.0789	0.0911	0.0779	0.0640	0.0775	0.0794	0.0719
*R*1	0.0336	0.0332	0.0365	0.0345	0.0574	0.0307	0.0341	0.0319
w*R*2	0.0763	0.0921	0.0829	0.0813	0.1631	0.0657	0.0803	0.0622
GooF	1.134	1.313	1.067	1.078	1.173	1.065	1.078	1.124
*M*u (mm^–1^)	22.147	21.434	26.043	31.051	28.404	31.744	26.38	31.584
*F*000	5600	5824	5480	5808	6047.0	5024	5456	3304
*Z*	4	4	4	4	4	4	4	2
Dx (g/cm^3^)	3.602	3.601	4.109	4.359	4.455	4.713	4.103	5.117
temp. (K)	100 (2)	100 (2)	100 (2)	100 (2)	100 (2)	100 (2)	100 (2)	100 (2)
wavelength (Å)	0.71073	0.71073	0.71073	0.71073	0.71073	0.71073	0.71073	0.71073

#### Raman and Infrared (IR) Spectroscopies

Raman spectra for complexes **1**–**8** were collected on a ReniShaw InVia Raman microscope. The spectra were collected using a 785 nm laser at varying powers, 20× magnification, and the extended scan setting with a spectral window of 1500–100 cm^–1^. The scan parameters were 10 s exposure times and 3 scans per spectrum. Data for each sample was collected in triplicate to minimize orientation effects. The Raman spectra were standardized by dividing the raw intensity values by laser power (200 mW, 100% for 785 nm laser) and exposure time. IR spectra for complexes **1**–**8** were collected in both mid-IR (MIR) (4500–400 cm^–1^) and far-IR (FIR) (400–100 cm^–1^) spectral windows under a vacuum on a Bruker VERTEX 70 V spectrometer using a platinum ATR microscope objective and the OPUS 8.5 software package. The resolutions for MIR and FIR measurements are 0.4 and 1 cm^–1^, respectively. Baseline corrections on the Raman and IR data were done using the PreDICT and Origin2024 software packages, respectively. Peak fitting for both Raman and IR spectra was done in Origin2024.^44^

#### Partial Least Squares (PLS) Analysis

PLS analysis was done in the Origin2024 software package by using the singular value decomposition (SVD) method.^44^ The leave-one-out cross-validation method was also used to prevent overfitting of the PLS models and to determine the number of latent variables to build into each model. PLS regression models were built for both structural distortion parameters (DPs) (skew angles, plane angles, and plane distances) and vibrational mode frequencies. The PLS analysis of the distortion parameters included unit cell parameters obtained from X-ray crystal structures (*a*, *b*, *c*, β, V), average *d*_U–O_ distances, the effective ionic radii (eIR) of the counterions, and the average *d*_U–M_ distances (M = Li­(I), Na­(I), K­(I), Rb­(I), Cs­(I)) as independent variables and the distortion parameters as the dependent variables. PLS models built to investigate correlations between the structural parameters and vibrational mode frequencies incorporated the DPs, counterion eIR, and average *d*_U–M_ distances as independent variables and the Raman or FIR mode frequencies as dependent variables. More details regarding the PLS analysis are included in the PLS Section of the Supporting Information **.**

## Results and Discussion

### Synthesis

UW_10_ was crystallographically characterized in 1975 by Golubev et al.;^35^ however, this complex has not received any subsequent research attention since this initial study. The most widely used procedure for synthesizing [An­(IV)/Ln­(III)­W_10_O_36_]^8/9–^ complexes is via the Peacock and Weakley method in which a solution of sodium tungstate is acidified with glacial acetic acid until the reaction solution pH is between 6.5 and 7.5.^14^ In recent work from our group focusing on LnW_10_ complexes,^15^ we found that this method produced a significant amount of byproducts in the form of sodium paratungstate, and hence, we used the procedure outlined by Mariichak and colleagues.^38^ This method employs a strong acid such as HNO_3_ or HCl as the acidifying agent, which reduces side product formation dramatically, and here, we extended this procedure to an actinide system for the first time. For the synthesis of **1**–**8**, the identity of the starting U­(IV) salt is also important as attempts to use U­(SO_4_)_2_ as a U­(IV) source resulted in the formation of an unknown dark gray precipitate that was likely a mixture of uranium oxide phases. In contrast, the addition of UCl_4_ into the sodium tungstate solution results in a solution color change (to brown) and the formation of a white precipitate. Since our UCl_4_ stock solution was in 2 M HCl, the pH of the reaction solution needed to be adjusted back to between 6.5 and 7 with NaOH. With each addition of a NaOH aliquot, in-growth of a dark green color can be seen in the U­(IV)-POM solution. The final color of the U­(IV)-POM solution can be either brown or dark green depending on the final pH, and after 1 day, the dark green solutions also turn brown. Despite the differences in reaction solution color, there are no differences in the final product as long as UCl_4_ is used as the uranium source and the final reaction pH is 6.5–7. After solutions are allowed to slowly evaporate for 3–5 days, dark brown crystals of **1**–**6** can be found in the mother liquor. The crystals of **6** can only be isolated during the first round of crystallization from the synthesis protocol for **1** and after subsequent rounds of recrystallization crystals of **1** evolve into a new polymorph (**1b**) and eventually into complex **2**.

The synthesis of complexes **7** and **8** follows the similar principles to those outlined above. Potassium and cesium tungstates along with potassium and cesium hydroxides are used instead of sodium tungstate and sodium hydroxide for the synthesis of compounds **7** and **8**, respectively. Besides the starting materials, the only difference between the synthesis method for **1**–**6** and the protocol for **7** and **8** is the first pH adjustment step. Without an initial pH adjustment, we found these reactions to be unsuccessful, yielding none of the desired products. Upon comparing the FTIR spectra of our K_2_WO_4_ and Cs_2_WO_4_ starting materials with that of our Na_2_WO_4_ source, we found that the sharp ν­(O–W–O) peak centered at 815 cm^–1^ that is characteristically observed for Na_2_WO_4_ is broader and contains multiple shoulders in our K_2_WO_4_ and Cs_2_WO_4_ spectra (Figure S1, Supporting Information).^45^ The FTIR spectrum of Na_2_WO_4_ also included stretching frequencies associated with the WO_4_^2–^ tetrahedron located in the 700–400 cm^–1^ region, which are absent in the K_2_WO_4_ and Cs_2_WO_4_ MIR spectra.^45^ These differences in tungstate FTIR spectra suggested that our K_2_WO_4_ and Cs_2_WO_4_ starting materials had potentially started to hydrolyze, thereby yielding unknown POM phases.^15,38,46–48^ It is well documented that tungsten-based POMs degrade under basic conditions;^1,2^ hence, adjusting the reaction solutions to higher pH values ensured that POM contaminants would break down. After basification, the synthesis protocol for **7** and **8** followed the same steps that were used to produce complexes **1**–**6**.

### Single-Crystal X-ray Structure Descriptions

Single-crystal X-ray diffraction data analysis revealed that complexes **1** and **3**–**8** are new structures, while complex **2** matched the previously reported structure from Golubev et al.^35^ Complexes **1**–**8** crystallized in seven different space groups, specifically complexes **1 (**Li_5_Na_3_[UW_10_]) and **6** (Li_8_[UW_10_]) in *P*2_1_/*n*, complex **2** (Na_8_[UW_10_]) in *C*2/*c*, complex **3** (K_4_Na_4_[UW_10_]) in *P*2_1_/*c*, complex **4** (Rb_6_Na_2_[UW_10_]) in *Cc*, complex **5** (Cs_5.5_Na_2.5_[UW_10_]) in *Pnma*, complex **7 (**K_8_[UW_10_]) in *P*2/*n*, and complex **8** (Cs_8_[UW_10_]) in *P*-1. Throughout **1**–**8**, UW_10_ moieties remain constant with differences in structures and packing due to the location of counterions and their interactions with lattice solvent and/or the POM clusters (Figure 1). The UW_10_ clusters in complexes **1**–**8** are formed by two lacunary [(W_5_O_18_)^6–^] (W_5_) POMs that are connected by a U­(IV) cation at the lacunary sites of the W_5_ moieties. The U­(IV) cations are coordinated by the four terminal oxygens of the lacunary sites from two W_5_ POMs, which results in U­(IV) coordination numbers of eight in all complexes and distorted square antiprismatic (D_4d_) symmetry for each of the U­(IV) cations. These metal-ion coordination number and point group symmetry characteristics for U­(IV) match what we observed for Ln­(III) cations in the LnW_10_ series,^15^ and average distances between the coordinating oxygens and U­(IV) cations (*d*_U–O_) are 2.371, 2.369, 2.368, 2.371, 2.365, 2.367, 2.371, and 2.361 Å for complexes **1**–**8**, respectively.

**Figure 1:**
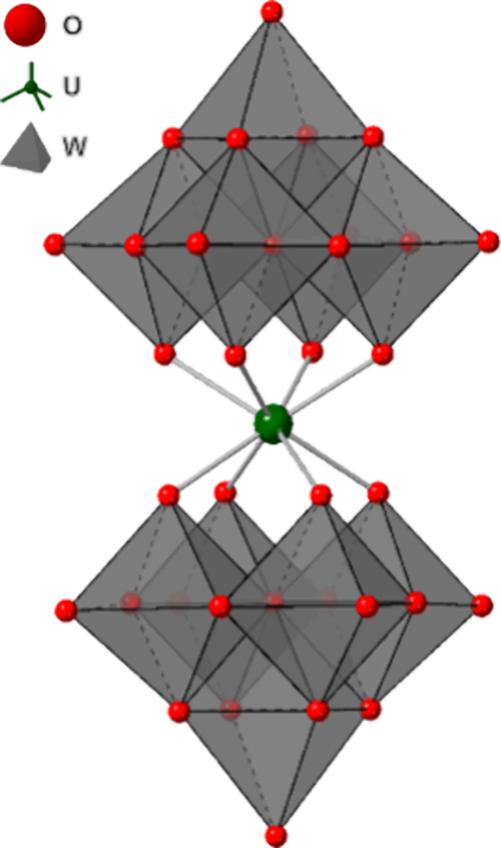
Polyhedral representation of the [U­(W_5_O_18_)_2_]^8–^ (UW_10_) moiety.

The two primary packing configurations observed in complexes **1**–**8** are highlighted in Figure 2. As counterions impact the vibrational and spin manifolds of f-element POMs, we have comprehensively tabulated interatomic distances that are discussed in this section, and these are compiled in Table S1 (Supporting Information). Overall, we found that larger alkali metal cations such as K­(I), Rb­(I), and Cs­(I) were generally located in the belt area of the clusters, closer to U­(IV) centers, while smaller alkali metal cations were primarily at the periphery of the POM complexes (Figures S2 and S4–S11, Supporting Information), and this observation suggests that larger counterions better associate with the clusters, especially in the belt region. This is illustrated to some extent by the asymmetric unit of **1**, Figure S2 (Supporting Information), and more so in complexes **2**–**5**, **7**, and **8**. Complex **1** features one UW_10_ moiety, five Li­(I) ions, and three Na­(I) ions. The packing of **1** shows **Na2** and **Na3** directly interacting with the terminal oxygens of the UW_10_ subunits via **O11** (*d*_Na2–O11_ = 2.447 Å) and **O25** (*d*_Na3–O25_ = 2.421 Å), respectively, and even though there is a higher abundance of Li­(I) in the lattice, we observe less interactions between the Li­(I) cations and the POM cluster. Interestingly, the closest distance between Li­(I) and U­(IV) atoms (*d*_U–Li_) is shorter than that between a Na­(I) atom and the U­(IV) metal center (*d*_U–Na_) with values of 5.613 Å (**U1–Li3**) and 5.837 Å (**U1–Na1**), respectively. However, the average distance between Li­(I) cations and U­(IV) cations (avg *d*_U–Li_ = 6.412 Å) is longer than that between Na­(I) cations and U­(IV) cations (avg *d*_U–Na_ = 6.358 Å). Complex **1**, Li_5_Na_3_[UW_10_], evolves after several rounds of recrystallization to form another polymorph of UW_10_Li (**1b**) and eventually breaks down into complex **2**. Complex **1b** has a completely different set of crystallographic parameters than **1**, Table S2 (Supporting Information), and it turns out to be the more stable polymorph as we often obtained complex **1b** when attempting to synthesize complex **1**. Unfortunately, the crystallography involving **1b** is very challenging and only a unit cell for this complex could be acquired despite the pristine physical morphology of the crystals, Figure S3 (Supporting Information). The source of disorder for **1b** might be the lack of association between the Li­(I) cations and the UW_10_ cluster, which would allow for more freedom of movement for the Li­(I) cations within the lattice, and the evolution of complexes of **1**/**1b** to **2** is probably a result of the thermodynamic instabilities that result from the transient nature of Li­(I) interactions with POM clusters and lattice water molecules.^49^

**Figure 2:**
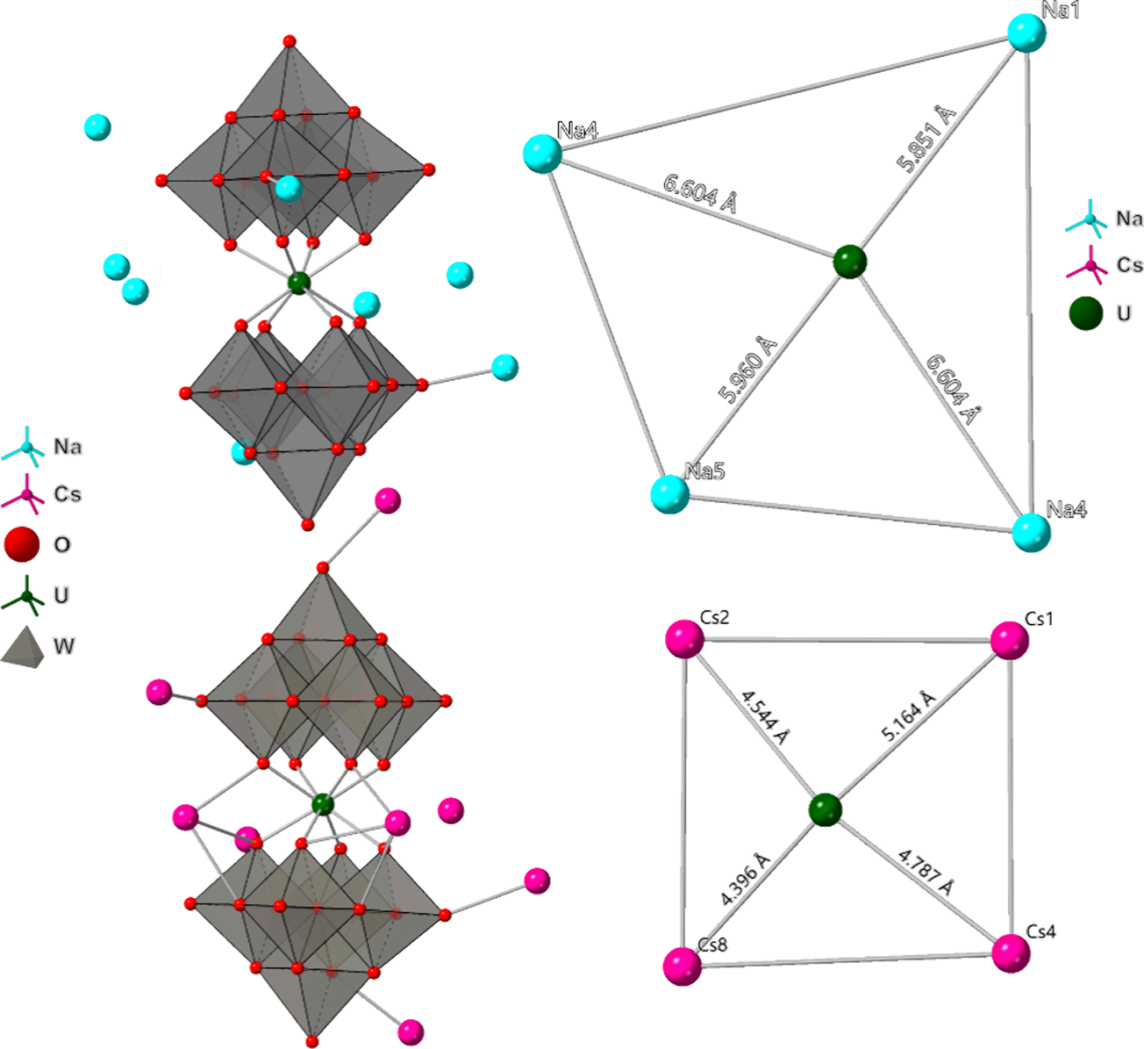
(Left) Polyhedral representations of complexes **2** and **8**. Oxygen atoms from lattice water molecules were excluded for clarity. (Right) Ball and stick representation of the UW_10_ belt area (boxed) for **2** and **8**. Complexes **2** and **8** serve as representative examples of species with lighter and heavier counterions, respectively. The heavier Cs­(I) ions in **8** are located closer to the cluster compared to the Na­(I) ions in **2**, indicating a preference for ion-pairing interactions between the Cs­(I) ions and the UW_10_ cluster.

Complex **2** is the only species with precedent in the literature, yet no description of the structure or details on the packing of the lattice are included in the work of Golubev et al.^35^ The asymmetric unit of **2**, Figure S4 (Supporting Information), contains half of a UW_10_ moiety and five unique Na­(I) cations that form a network with lattice water molecules that are localized within the asymmetric unit. The closest Na­(I)–U­(IV) distance (*d*_U–Na_) for **2** is 5.851 Å (**U1–Na1**), and the average *d*_U–Na_ distance is 6.494 Å. Comparing **2** with other tetravalent f-element analogues, specifically Na_8_[Ce­(IV)­W_10_] and Na_8_[Th­(IV)­W_10_], the Na­(I) cations occupy identical positions with similar closest Na­(I)–M­(IV) distances of 5.852 Å (*d*_Ce(IV)–Na_) and 5.863 Å (*d*_Th–Na_), respectively, Figure S4 (Supporting Information). Differences in packing begin to manifest with larger counterions, as exemplified by the asymmetric unit of **3**, Figure S5 (Supporting Information), which contains a full UW_10_ moiety, four Na­(I) cations, and four K­(I) cations. Inspection of the packing for **3** reveals that all K­(I) cations interact directly with the UW_10_ moiety, while Na­(I) cations in the asymmetric unit of **3** are located farther from the UW_10_ moiety and only the disordered sodium atom **Na4A/B** directly interacts with the cluster through oxygen atom **O7**. The greater association between K­(I) cations and the UW_10_ cluster is reflected in the closest interaction distances (*d*_U–M_) for each of the lattice counterions, and for **3**, these *d*_U–K_ and *d*_U–Na_ values are 4.035 Å (**U1–K2)** and 4.421 Å (**U1–Na3**). Moreover, the average *d*_U–K_ and *d*_U–Na_ distances also support the observation that association interactions with K­(I) are stronger in complex **3** with values of 6.189 Å (*d*_U–K_) and 6.375 Å (*d*_U–Na_), respectively.

The asymmetric unit of complex **4**, Figure S6 (Supporting Information), contains a full UW_10_ moiety, six Rb­(I) cations, and two Na­(I) cations. There are six unique positions for the Rb­(I) cations in the asymmetric unit of **4** with **Rb1**, **Rb2**, **Rb4**, and **Rb5** located at the periphery of the UW_10_ cluster and **Rb3** and **Rb6** located in the belt area of the structure where they engage in direct ion-pairing interactions with the UW_10_ POM. The two unique Na­(I) cations in **4** are part of a water network in the lattice and do not interact directly with the UW_10_ cluster, instead participating in interactions with **Rb1** and **Rb5**. This configuration of heavier counterions being located closer to the metal POM compared to the lighter counterions is reflected in the much shorter average *d*_U–Rb_ distance (6.010 Å) compared to the average *d*_U–Na_ distance (8.102 Å). The closest alkali metal–U­(IV) interaction distances are 4.212 Å (**U1–Rb3**) and 7.992 Å (**U1–Na2**), respectively, which further demonstrates that there are significant differences in interactions with the cluster for Rb­(I) and Na­(I). The increase in direct ion-pairing interactions with heavier counterions in both **3** and **4** is due to stronger electrostatic interactions between U­(IV) and the alkali metal cations, and similar findings have also been noted by Colliard and Deblonde and Zagrebin et al. in their studies on f-element POMs.^18,49^ The asymmetric unit of **5** contains half of a UW_10_ cluster, 2.75 Cs­(I) cations, and 1.25 Na­(I) cations, Figure S7 (Supporting Information), and the findings here are consistent with the observations for **3** and **4** as all Cs­(I) cations directly interact with the UW_10_ cluster. The two Na­(I) cations in **5** are part of a water network in the lattice, similar to **4**, and the preference for heavier counterions to engage in ion-pairing interaction with the UW_10_ cluster in **5** is reflected in the differences in average *d*_U–M_ distances, 5.803 Å (*d*_U–Cs_) and 7.646 Å (*d*_U–Na_), and the closest alkali metal–U­(IV) interaction distances, 4.563 Å (**U1–Cs3A-B**) and 6.970 Å (**U1**-**Na2**).

For complex **6**, the asymmetric unit contains a full UW_10_ cluster and eight Li­(I) cations, Figure S8 (Supporting Information). All Li­(I) cations in **6** exhibit typical Li­(I) coordination numbers, and notably, seven out of eight Li­(I) cations in **6** also interact directly with the UW_10_ cluster. The closest alkali metal cation–U­(IV) interaction distance (*d*_U–Li_) for **6** is 3.352 Å (**U1–Li1**), and the average *d*_U–Li_ is 5.766 Å. Both *d*_U–Li_ and average *d*_U–Li_ values for complex **6** are the shortest values found in this study. The interactions and proximity of Li­(I) cations to the UW_10_ cluster in **6** may seem inconsistent with our previous observations for complexes **1**–**5**, where we noted an increase in stronger ion-pairing interactions with larger alkali metal counterions. However, complex **6** is also short-lived and evolves to form complex **1** after one round of recrystallization, which is consistent with our earlier observation that the interactions between Li­(I) cations and UW_10_ clusters are limited and energetically unfavorable. As such, the structural parameters for complex **6** add complexity to our findings related to the UW_10_ system and indicate that the structural trends that we have noted are most applicable to the thermodynamically stable crystalline phases of UW_10_.

Complexes **7** and **8** were synthesized by varying the alkali tungstate starting material rather than introducing counterions via metal chloride solutions, and these species were characterized in an identical manner to complexes **1**–**6** to broaden our investigation into the effects of counterion size on lattice packing. The asymmetric unit of **7** contains two halves of two unique UW_10_ moieties and eight K­(I) counterions Figures S9 and 10 (Supporting Information), and the two UW_10_ (**U1** and **U2**) clusters are related to each other through a *n*-glide plane and connected by three K­(I) cations (**K1**, **K2**, and **K3**). Potassium atoms **K4**–**7** interact with only the **U2** cluster and are located at the periphery of the complex, while **K8**–**10** only interact with the **U1** cluster and are located in the belt region of the POM. The closest alkali metal–U­(IV) interaction distances (*d*_U–K_) for **U1** and **U2** are 4.065 Å (**U1–K9A/B**) and 5.711 Å (**U2–K3**), and the average K­(I)–U­(IV) interaction distance (avg *d*_U–K_) for complex **7** is 7.190 Å. The closest and average interaction distances between the K­(I) and U­(IV) cations in **7** are longer than those in complex **3**. Interestingly, the average *d*_U–K_ distance in **7** is also longer compared to the average *d*_U–Na_ distances in **2**, and while all of the K­(I) cations in **7** interact with the cluster, only one Na­(I) cation in **2** interacts with the UW_10_ moiety. These findings illustrate that while heavier alkali metal counterions preferentially participate in ion-pairing interactions, not all interactions are equivalent in distance or their ability to effectively modulate metal-ion symmetry or crystal field splitting, which is a notable finding as it relates to tuning complexes for applications in QIS. Finally, the asymmetric unit of **8** contains a full UW_10_ moiety and eight Cs­(I) counterions, Figure S11 (Supporting Information). Evaluating the packing of **8** reveals that **Cs1** and **Cs2** occupy the belt area of the structure, while the rest of the Cs­(I) ions in **8** are located on the periphery of the cluster. The closest Cs­(I)–U­(IV) distance (*d*_U–Cs_) for this complex is 4.396 Å (**U1–Cs8**), and the average Cs­(I)–U­(IV) distance (avg *d*_U–Cs_) is 5.945 Å. Both values in complex **8** are shorter than the closest and average distance values noted for Na­(I) and K­(I) ions in **2** and **7**, which confirm that f-element POMs prefer ion-pairing interaction with larger counterions.

### Structural Discussion

Complexes **1**–**5** are the first examples of the same POM being prepared and structurally characterized with five different counterions spanning the alkali metal series. Comparisons of the asymmetric units for **1**–**5** have demonstrated an evolution in POM interactions with the second coordination sphere as lighter counterions, Li­(I) and Na­(I), primarily form coordination networks with lattice water molecules, while heavier counterions, K­(I), Rb­(I), and Cs­(I), prefer ion-pairing interactions that result in greater association between counterions and UW_10_ clusters. These differences are best exemplified by comparing the configuration of counterion networks within the asymmetric units of complexes **1** and **3** (Figures S2 and S5, Supporting Information). Despite the higher number of Li­(I) cations compared to Na­(I) cations within the lattice in **1**, none of the Li­(I) cations directly interact with the UW_10_ cluster, whereas in complex **3**, all the K­(I) cations directly interact with the UW_10_ moiety and only one Na­(I) cation coordinates with the UW_10_ POM even though there is an equal amount of Na­(I) and K­(I) cations within the lattice. The comparison of complex **2** with complexes **7** and **8** provides a platform for directly evaluating counterion size effects on lattice packing as these species feature a single type of alkali metal cation in the lattice and we note an increase in packing density going from **2** to **7** and **8** that coincides with the increase in ion-pair interaction between the UW_10_ cluster and the heavier counterions. The decrease in unit cell volumes while keeping a constant *Z* value seen in **2** (6147.4(4) Å^3^, *Z* = 4), **7** (5098.4(3) Å^3^, *Z* = 4), and **8** (2530.96(15) Å^3^, *Z* = 2) captures this trend clearly as reduced volumes are associated with denser lattice packing. The volume of complex **6** (4132.3(2) Å^3^, *Z* = 4) is noteworthy, as it is significantly smaller than any other complexes described herein, yet this species is a likely outlier, as highlighted above, so we have not included it as part of our structural comparisons.

In general, tetravalent UW_10_ species characterized here crystallized in monoclinic space groups, while the trivalent LnW_10_Na complexes we studied recently exclusively crystallize in the triclinic space group *P*-1.^15^ There are instances in the literature where LnW_10_Na complexes crystallize in monoclinic or orthorhombic space groups as well;^50,51^ however, in our experience, these phases are only accessible when additional reactants are added during synthesis and crystallization procedures, so we will limit our comparisons here to UW_10_ complexes and LnW_10_Na species from our recent work.^15^ The lesser number of counterions required to charge balance the tetravalent complexes herein is a likely cause for the differences in packing between tetravalent and trivalent complexes, although the latter systems can provide valuable information when compared to tetravalent actinide complexes as it relates to delineating size and charge effects of the metal center in the POM complexes. The ionic radius for eight coordinate U­(IV) is 1.00 Å, and this is very similar to Er­(III) (1.004 Å).^52^ Comparing An/Ln–O bond distances in [Er­(W_5_O_18_)_2_]^9–^ (ErW_10_) and UW_10_ reveal that *d*_An–O_ for UW_10_ (*d*_An–O_ = 2.368–2.371 Å) are slightly longer than what was noted in ErW_10_ (*d*_Ln–O_ = 2.3601 Å) and instead are more comparable to *d*_Ln–O_ values for [Ho­(W_5_O_18_)_2_]^9–^ (HoW_10_; Ho­(III) = 1.015 Å, *d*_Ln–O_ = 2.366 Å).^15,52^ This is an unexpected observation, as metal–POM interactions with W_5_ ligands are primarily expected to be ionic in bonding character. As such, one would expect the bond distances to decrease when moving from +3 to +4 metal centers of similar sizes; however, here we note that M–O bonds have been elongated. This could mean that the sandwich configuration of two W_5_ ligands has a fixed cavity size that limits changes in *d*_An/Ln–O_ distances for metal centers of similar sizes, but this explanation would not account for the elongation of the U­(IV)–O distances observed herein. Another possible explanation is that the interaction between W_5_ ligands and metal centers are softer in nature than anticipated, which would mean that a hard–soft acid base pairing with harder tetravalent metal centers would be less preferred, thereby resulting in elongated *d*_An/Ln(IV)–O_ distances. The average *d*_An/Ln–O_ values for Na_8_[Th­(IV)­W_10_] (Th­(IV) = 1.05 Å, *d*_An–O_ = 2.423 Å) and Na_8_[Ce­(IV)­W_10_] (Ce­(IV) = 0.97 Å, *d*_Ln–O_ = 2.356 Å) also reveal the same elongation when compared with their respective Ln­(III) size analogues, Na_9_[Gd­(III)­W_10_] (Gd­(III) = 1.053 Å, *d*_Ln–O_ = 2.410 Å) and Na_9_[Lu­(III)­W_10_] (Lu­(III) = 0.977 Å, *d*_Ln–O_ = 2.337 Å), which supports the latter hypothesis. Comparing *d*_An/Ln–O_ distances for the different tetravalent species (Th­(IV), U­(IV), and Ce­(IV)) reveals that M­(IV)–O distances increase as the ionic radii of the M­(IV) center increases with similar magnitude to what is seen in the trivalent LnW_10_ series.^15^ Analysis of the crystal structures for complexes **1**–**8** also shows that, in general, the larger alkali metal counterions are positioned closer to the UW_10_ clusters compared to the smaller metal counterions. This finding suggests that the larger, softer alkali metal counterions associate more strongly with the W_10_ POM cluster, which is in line with the hypothesis explaining the elongation of *d*_An/Ln–O_ distances for +3 and +4 metal centers of similar sizes and consistent with the known ability of POMs to delocalize negative charges throughout the entire clusters.^2,49^

Since our overall hypothesis was that UW_10_ POMs could act as potential electron spin qubits, we investigated structural distortion parameters (DPs) for each cluster, specifically the skew angle (SA), plane angle (PA), and plane distance (PD), which can provide information about the extent of deviations from ideal D_4d_ symmetry in complexes **1**–**8**. In the previous section, we observed lattice packing modulations that result from changing the composition and configuration of the secondary sphere elements. Based on our previous work, we anticipated that these changes in lattice packing for complexes **1**–**8** would also manifest in DPs for these species.^15^ The details regarding the measurement of the DPs are located in Supporting Information (Figures S12 and S13). Distortion parameters for complexes **1**–**8** are detailed in Table 2, and to account for the different ionic radii of the counterions as well as the variations in adopted coordination numbers, we combined the ionic radii of the counterions present within each complex and averaged these values to generate an effective ionic radii (eIR) value.^52^ This normalization process enables numerical comparison between complexes **1**–**8** and provides a tool for quantitatively accounting for differences that result from varying compositions and configurations of counterions within the lattice (e.g., K_4_Na_4_[UW_10_] vs K_8_[UW_10_]) of POM complexes. Details regarding the calculation of eIR and average *d*_U–M_ distances are located in the Supporting Information (Equation S1, Table S3).

**Table 2:** Distortion and Structural Parameters for Complexes **1**–**8**

complexes	skew angle (°)	plane angle (°)	plane distance (Å)	effective ionic radius (Å)	average *d*_U–Li/K/Rb/Cs_ (Å)	average *d*_U–Na_ (Å)	average *d*_U–M_ (Å)
UW_10_Li (**1**)	0.935	2.29	0.011	0.736	6.412	6.358	6.396
UW_10_LiF (**6**)	7.625	2.29	0.011	0.590	5.766	N/A	5.766
UW_10_Na (**2**)	1.100	0.06	0	1.014	6.494	6.494	6.494
UW_10_K (**3**)	2.250	2.37	0.023	1.228	6.189	6.375	6.293
UW_10_KF (**7**)	2.238	3.67	0	1.473	7.215	N/A	7.190
UW_10_Rb (**4**)	0.303	2.14	0.017	1.425	6.010	8.102	6.531
UW_10_Cs (**5**)	0.260	1.00	0.069	1.520	5.803	7.646	6.417
UW_10_ CsF (**8**)	3.343	2.41	0.027	1.736	5.941	N/A	5.941

The effective ionic radii were then plotted against the different distortion parameters, as shown in Figure 3. In Figure 3a, we observe an increase in SAs as the counterion eIR increases, except for complexes **4**, **5**, and **6**, which are outliers in this series. Figure 3b reveals that there are no clear trends between the plane angle and counterion eIR values other than a qualitative increase in PA values as eIR values increase, with complexes **1** and **6** being clear outliers. Figure 3c also shows that there is not a clear correlation between PDs and counterion eIR values other than a general increase in PDs as the eIR values increase. The PD value of complex **6** is also an outlier as it is significantly higher than those of the other complexes despite the small eIR of **6**. Across Figure 3a–c, complex **6** displays anomalous behavior compared to other complexes within **1**–**8**, and this may be a result of the transience and instability of this species, which is not comparable with the greater stability that we observed for complexes **1**–**5**, **7**, and **8**. The average distance between U­(IV) cations and alkali metal counterions is another variable that was investigated and compared with the structural DPs for complexes **1**–**8**. The average U­(IV)–counterion distances (average *d*_U–M_; M = Li­(I), Na­(I), K­(I), Rb­(I), and Cs­(I)) are plotted against the DPs in Figure 4. Figure 4a reveals that SAs decrease as the average *d*_U–M_ distance increases, except for complex **7**. There is also a general decrease in PAs as the average *d*_U–M_ distance decreases (Figure 4b), consistent with the trend that we noted for SAs, with complex **7** again being a clear outlier in this comparison. The comparison of PDs with average *d*_U–M_ distance reveals that plane distances in **1**–**8** decrease as the average *d*_U–M_ distance increases, mimicking the observations from SA and PA comparisons, with complex **5** being the outlier in this plot. Worthy of an additional comment, it is notable that **6** displays unusually high degrees of distortion, as observed in SA and PD values that are almost twice as large as the distortion parameter values that we obtained for other complexes. This further suggests that complex **6** is an unstable species, which may be a result of weak interactions between Li­(I) cations and the UW_10_ cluster that allow for greater deviations from ideal symmetry for this complex.

**Figure 3:**
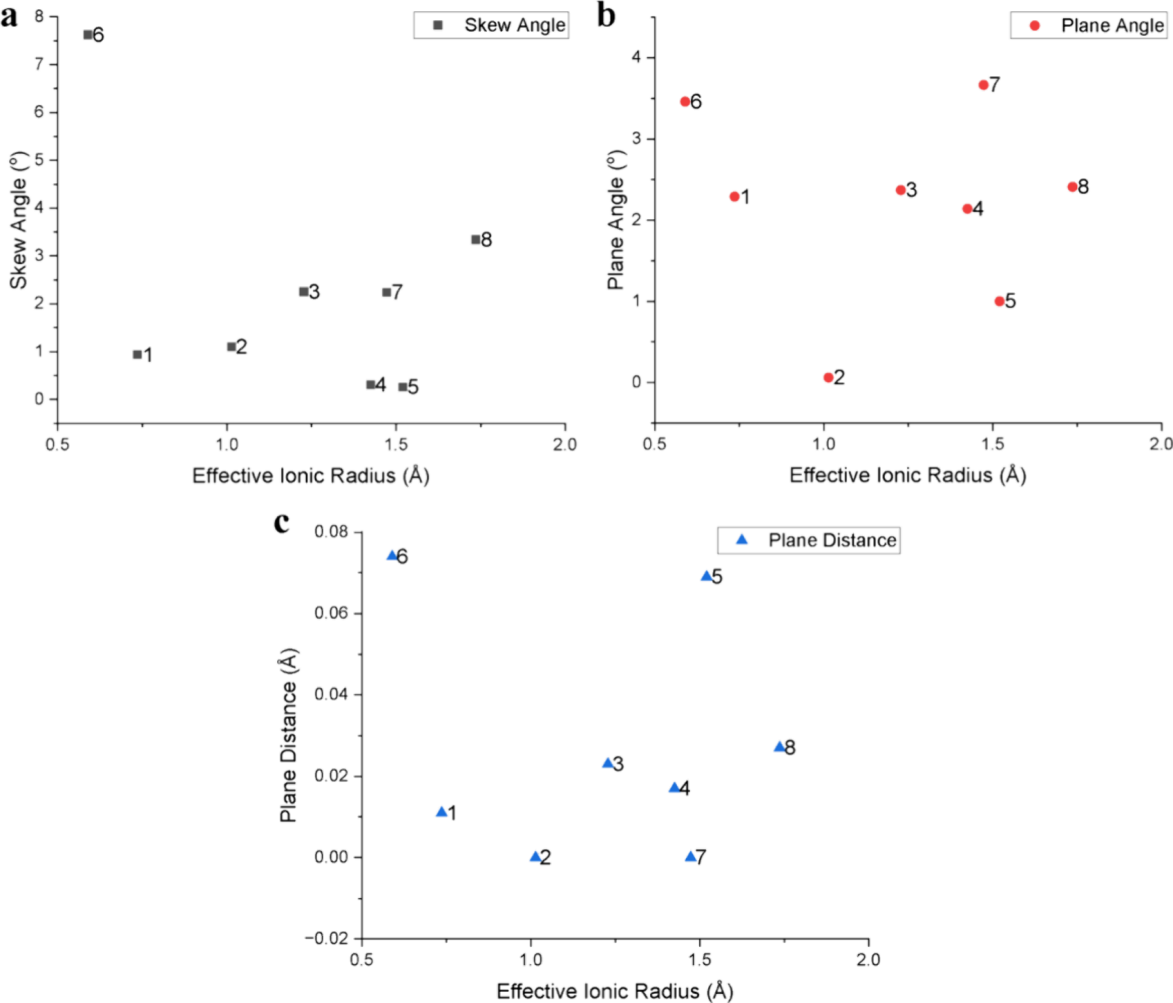
Plots of skew angle (SA) vs. counterion effective ionic radius (eIR) (a), plane angle (PA) vs. eIR (b), and plane distance (PD) vs. eIR (c) for complexes **1**–**8**.

**Figure 4:**
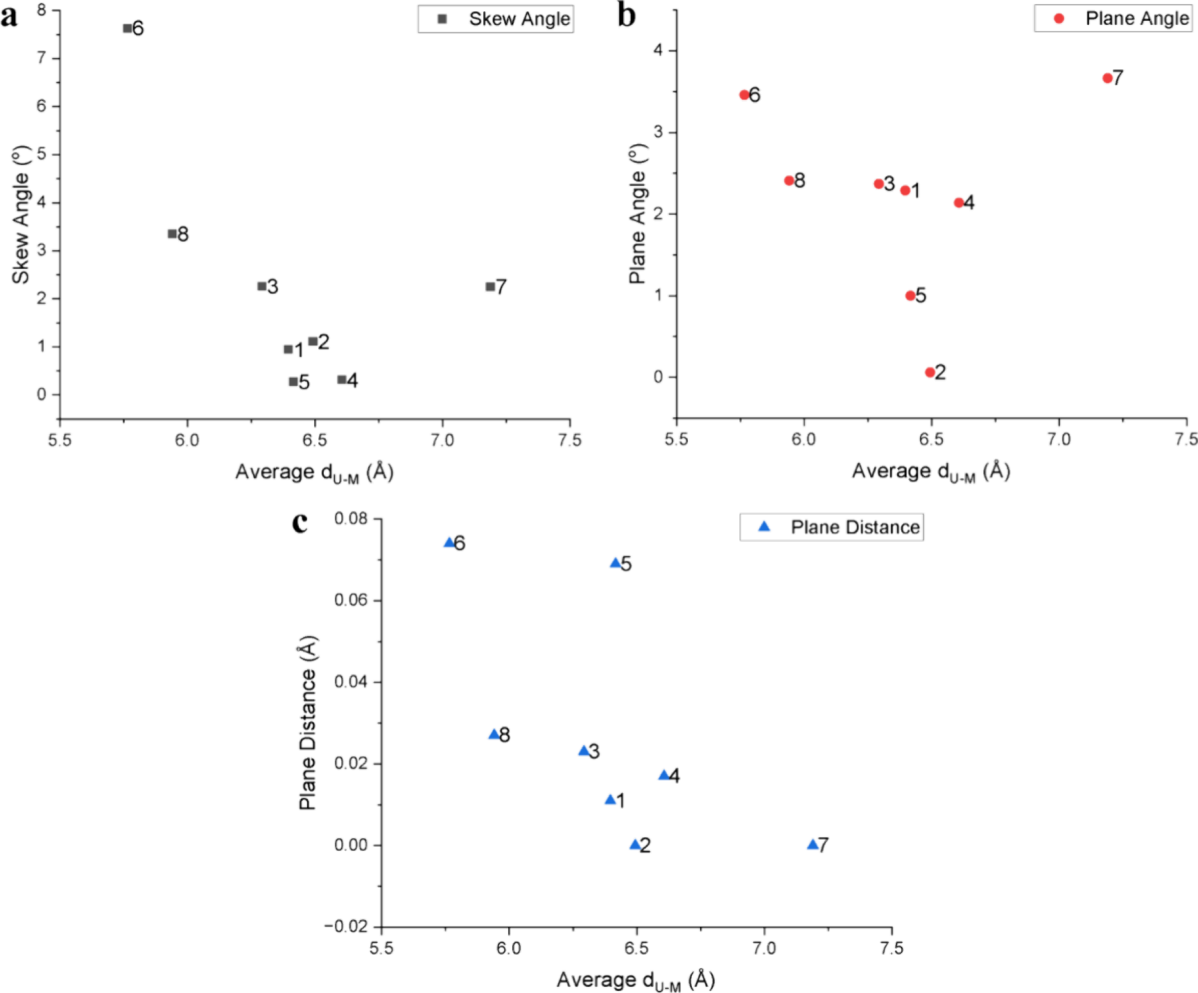
Plots of SA vs. average *d*_U–M_ distance (a), PA vs. avg *d*_U–M_ distance (b), and PD vs. avg *d*_U–M_ distance (c) for complexes **1**–**8**.

To more rigorously understand how counterions affect complex DPs, partial least-squares analysis (PLS) was conducted, Figures S14–S19 (Supporting Information). Counterion eIR and average *d*_U–M_ distance were chosen as independent variables to capture how changes in counterion size and interaction distances with POM clusters impacted DPs, and unit cell parameters and average *d*_U–O_ distances were chosen as independent variables to capture how changes in overall lattice packing impacted DPs. The full details regarding the PLS analysis can be found in the Partial Least Square Analysis section of the Supporting Information. Initially, the PLS regression models were unable to model the observed DP variances while passing cross-validation tests that prevent model overfitting, Figure S14 (Supporting Information). When values involving complex **6** were removed from the analysis, cross-validated models could be built for SA and PD values and the independent variables described above. The rationale to remove complex **6** from this analysis derives from the observation that complex **6** is highly unstable compared to the rest of the species investigated even when compared to complex **1** or polymorph **1b**. This instability suggests that complex **6** is likely a kinetic product of the crystallization process, while all other complexes described herein are thermodynamic products. As such, we believe that **6** belongs to a different class of unstable crystal phases that should not be compared with complexes **1**–**5**, **7**, and **8**, and we updated our PLS models accordingly. According to the PLS analysis model built without data from **6**, four factors can be synthesized out of the independent variables to account for 87.9% of the variance seen in skew angle values, Figure S15 (Supporting Information), and based on the loading scores, we were able to narrow the cause of SA variance to unit cell volume and counterion eIR values (Figures S15 and S16, Supporting Information). As the latter affects the packing and thus the unit cell volume, it is highly probable that counterion eIR has the largest impact on SA values. The PLS model where plane distance is the dependent variable produces one latent variable that accounts for 69.1% of the variance observed in these DP values, Figures S18 and S19 (Supporting Information). Loading scores show that the β angle and average *d*_U–M_ distance are the two most correlated variables with complex PDs, and consistent with our analysis above, we attribute changes in average *d*_U–M_ distances as the primary driver of plane distance variance. Despite the lack of statistical correlation found between PA values and the independent variables, Figure S17 (Supporting Information), PLS analysis still found that structural parameters, especially the counterion eIR and the average *d*_U–M_ distance, were highly correlated with PA values, which supports our approach for modifying the effective symmetry around the metal center by tuning the composition and packing of lattice counterions.

### Vibrational Spectroscopy

The FIR and Raman spectra of complexes **1**–**8** were collected on single crystals, and features were identified through peak fitting regimes executed using the Origin2024 software package. This section will be split into two different parts with the initial discussion focused on the FIR spectra and the second part describing the results from Raman spectra. Raw data, baseline corrected spectra, and fit parameters for the FIR and Raman spectra of complexes **1**–**8** can be found in Figures S20–S37 (Supporting Information), and the data here are the first examples of vibrational spectroscopy measurements conducted on UW_10_ POMs. Despite the similarities between the first coordination spheres of UW_10_ POMs, FIR and Raman spectra exhibit distinct changes in vibrational features that we attribute to differences in interactions between POM clusters and alkali metal counterions, Figures 5 and 6. FIR vibrational assignments were made using peaks that had been previously identified in the literature and were primarily based on our recent work and a 2021 study from Blockmon et al.^15,53^

**Figure 5:**
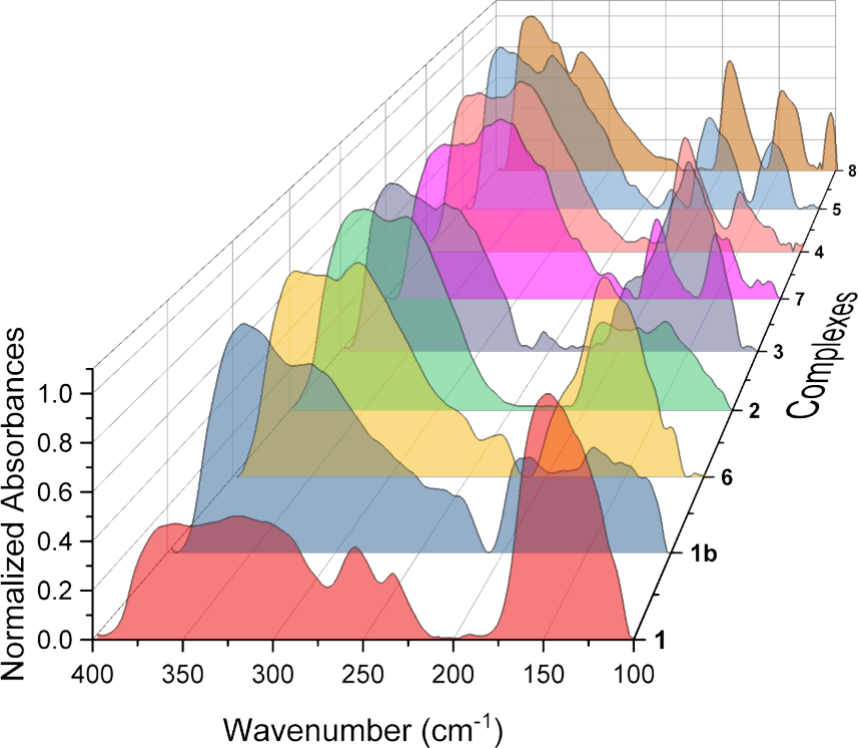
Normalized and baseline-corrected far-IR spectra for complexes **1**–**8**.

**Figure 6:**
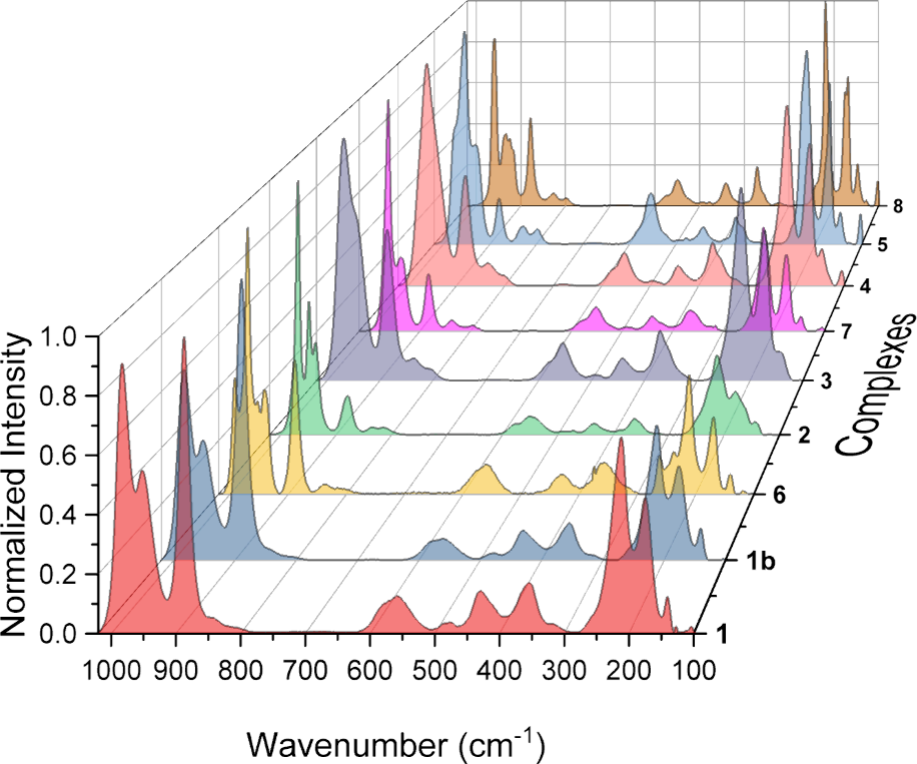
Normalized and baseline-corrected Raman spectra for complexes **1**–**8**.

Since the FIR spectra of complex **2**, Na_8_[UW_10_], is akin to the FIR spectra of HoW_10_, it was chosen to be our model species for vibrational modes assignments, which were then translated to complexes **1** and **3**–**8**. Based on the peak fitting regime, complex **2** possesses six features in the FIR (Figure S23, Supporting Information), similar to what was observed for HoW_10_. The peak centered at 363 cm^–1^ matches well with the δ/ρ­(HoO_4_) mode and was assigned here as the δ/ρ­(UO_4_) mode. The peak at 322 cm^–1^ is consistent with the frequency of the ν/ρ­(HoO_8_) mode; thus, we assigned it here as the ν/ρ­(UO_8_) mode. The peak centered at 191 cm^–1^ is in agreement with where we observed the ρ­(HoO_8_) mode, and here, it was assigned as the ρ­(UO_8_) mode. Finally, the peak centered at 145 cm^–1^ was assigned as a ν­(WO_5_)_2_ mode, which is also consistent with results from our group and Blockmon and colleagues.^15,53^ The peaks at 169 and 129 cm^–1^ do not match with any known FIR stretches in the literature for U­(IV), and thus, they were not assigned. The complete list of FIR vibrational mode assignments for complexes **1**–**8** can be found in Table 3.

**Table 3:** Far-IR (FIR) Mode Assignments for Complexes **1**–**8**

complex	δ/ρ(UO_4_) modes (cm^–1^)	ν/ρ(UO_8_) modes (cm^–1^)	ρ(UO_8_) modes (cm^–1^)	ν(WO_5_)_2_ modes (cm^–1^)	unassigned (cm^–1^)
UW10 Li (**1**)	360.77, 373.34	329.75		142.32	119.93, 158.27, 232.46, 253.90, 291.80
UW10 Li 2 (**1b**)	353.83, 370.82	314.41	195.11	146.06	110.79, 124.70, 165.85, 183.84, 221.36, 232.43, 259.25
UW10 LiF (**6**)	350.91, 371.09	321.70	192.10	142.43	119.70, 130.10, 163.88, 229.09, 253.65, 293.85
UW10 Na (**2**)	363.21	321.98	191.14	145.02	128.99, 168.99
UW10 K (**3**)	358.22, 374.52	322.50	194.41	145.24	127.24, 165.74, 251.96, 291.73
UW10 KF (**7**)	356.03, 369.71, 378.79	312.47	194.74	151.25	110.67, 118.09, 142.21, 180.85, 215.56, 227.72, 250.63, 266.81, 277.63
UW10 Rb (**4**)	361.33, 375.85	329.30	194.37	148.90	124.05, 136.54, 181.45, 280.69, 297.05
UW10 Cs (**5**)	360.15, 375.90	325.75	195.29	144.34	131.83, 178.66, 223.84, 250.90, 286.42
UW10 CsF (**8**)	363.38, 378.04	325.63	195.29	149.30	104.68, 137.50, 184.56, 246.39, 299.95, 345.86

Comparing the FIR spectra across complexes **1**–**8**, the spectrum of **2** is qualitatively similar to those of **1, 3**, and **6**, while complexes **4**, **5**, **7**, and **8** feature spectra that are different from complex **2** yet similar to each other. The FIR spectrum for complex **1**, Li_5_Na_3_[UW_10_], exhibits nine features, Figure S20 (Supporting Information), compared to the six features observed for **2**. The peaks centered at 373 and 361 cm^–1^ were assigned as δ/ρ­(UO_4_) modes, while the peaks at 330 and 142 cm^–1^ were assigned to ν/ρ­(UO_8_) and ν­(WO_5_)_2_ modes, respectively. There is no peak that can be associated with the ρ­(UO_8_) mode in **1**. The additional peaks centered at 292, 254, 232, 158, and 120 cm^–1^ do not match with any known FIR stretches in the literature for U­(IV) and thus were not assigned. The bands observed at 254 and 232 cm^–1^ may originate from LiCl contamination in the sample; however, this is unlikely, as the collections are conducted on single crystals and done in triplicate. The FIR spectrum for polymorph complex **1b** was also collected and features 12 peaks, Figure S21 (Supporting Information). The peaks centered at 371 and 354 cm^–1^ were assigned as δ/ρ­(UO_4_) modes, while the peaks centered at 314, 195, and 146 cm^–1^ were assigned to the ν/ρ­(UO_8_), ρ­(UO_8_), and ν­(WO_5_)_2_ modes, respectively. The spectrum for **1b** also includes the peaks seen in **1** at 259 and 232 cm^–1^ as well as the two unassigned peaks at 166 and 125 cm^–1^; however, the FIR spectrum for **1b** also features extra peaks centered at 221, 184, and 111 cm^–1^. As these peaks do not match with known FIR peak assignments and computational calculations are outside our abilities and the scope of this study, the extra peaks for **1b** were not assigned. The FIR spectrum for complex **6**, Li_8_[UW_10_], includes 11 peaks that are centered at frequencies that are very similar to those observed for complex **1**, Figure S22 (Supporting Information). The peaks at 371, 351, 322, 192, and 142 cm^–1^ were assigned as δ/ρ­(UO_4_), ν/ρ­(UO_8_), ρ­(UO_8_), and ν­(WO_5_)_2_ modes, while the remainder of the peaks centered at 294, 254, 229, 164, 130, and 120 cm^–1^ do not match with known FIR peak assignments for W_10_ POM systems and were not assigned. Overall, the FIR spectra of **1**, **1b**, and **6** display similar features with the notable exception of the absence of the ρ­(UO_8_) stretch in the spectrum of **1**. Finally, the FIR spectrum for complex **3** exhibits nine features similar to those of **1**, Figure S24 (Supporting Information), and the peaks centered at 374, 358 and 323 cm^–1^ were assigned as δ/ρ­(UO_4_) and ν/ρ­(UO_8_) modes, while the peaks centered at 194 and 145 cm^–1^ were assigned as ρ­(UO_8_) and ν­(WO_5_)_2_ modes, respectively. The remaining peaks centered at 292, 252, 166, and 127 cm^–1^ do not match any known FIR stretches in the literature for U­(IV) and thus were not assigned.

The FIR spectrum for complex **4**, Rb_6_Na_2_[UW_10_], includes 10 peaks with those centered at 376 and 361 cm^–1^ assigned as δ/ρ­(UO_4_) modes and the peaks centered at 329, 194, and 149 cm^–1^ assigned as ν/ρ­(UO_8_), ρ­(UO_8_), and ν­(WO_5_)_2_ modes, respectively, Figure S26 (Supporting Information). The remaining features at 291, 281, 181, 136, and 124 cm^–1^ do not match with known FIR peak assignments and were not assigned. The difference in spectral features between complexes **4, 5, 7,** and **8** and complexes **1**–**3** and **6** can be most clearly observed in the lower wavenumber region of the FIR (250–100 cm^–1^). The peak centered around 165 cm^–1^ in the spectra for complexes **1**–**3** and **6** is absent for complexes **4**–**8** (excluding **6**), and instead, lower energy features are split into a series of smaller peaks. The FIR spectrum for complex **5**, Cs_5.5_Na_2.5_[UW_10_], also includes 10 features that were similarly assigned to **4**, Figure S27 (Supporting Information). Peaks centered at 376, 360 and 326 cm^–1^ were assigned as δ/ρ­(UO_4_) and ν/ρ­(UO_8_) modes, while the peaks centered at 195 and 144 cm^–1^ were assigned as ρ­(UO_8_) and ν­(WO_5_)_2_ modes, respectively. The remaining peaks centered at 286, 251, 224, 179, and 134 cm^–1^ do not match known FIR peak assignments for W_10_ POM systems and were not assigned as a result. The FIR spectrum for complex **7**, K_8_[UW_10_], includes the most features (Figure S25, Supporting Information) we observed in this family of complexes with 15 peaks noted between 400 and 100 cm^–1^. The peaks centered at 370 and 356 cm^–1^ were assigned as δ/ρ­(UO_4_) modes, while the peaks centered at 312, 195, and 151 cm^–1^ were assigned as ν/ρ­(UO_8_), ρ­(UO_8_), and ν­(WO_5_)_2_ modes, respectively. The remaining peaks that were unassigned as they do not match any known FIR assignments for U­(IV) complexes are located at 278, 267, 251, 228, 216, 181, 142, 118, and 111 cm^–1^. The abundance of features in the FIR spectrum of **7** can be explained by its distinct crystal structure as **7** is the only species with two unique UW_10_ moieties within its unit cell, which means that it is possible to observe the same mode for each moiety with peak splitting due to the difference in local environments. The FIR spectrum for complex **8**, Cs_8_[UW_10_], includes 11 features that are very similar to those included in the FIR spectrum for **5** (Figure S28, Supporting Information). The peaks centered at 378, 363, 326, 196, and 149 cm^–1^ are assigned as δ/ρ­(UO_4_), ν/ρ­(UO_8_), ρ­(UO_8_), and ν­(WO_5_)_2_ modes, while the stretches centered at 346, 300, 246, 185, 137, and 107 cm^–1^ do not match with known vibrational frequencies for W_10_ POMs, so these peaks remain unassigned.

For the Raman spectra of **1**–**8**, we once again utilized vibrational assignments from the literature, mainly from the works of Kazanskii and colleagues and Shiozaki et al.,^46,48^ to aid in making peak fitting assignments. Similar to our treatment of FIR results, we used complex **2**, Na_8_[UW_10_], as our model species for vibrational mode assignments, and the Raman spectrum for **2** exhibits 15 features over the spectral range 1050–100 cm^–1^, Figure S32 (Supporting Information). The peaks centered at 969, 949, and 937 cm^–1^ were assigned as ν­(W=O) modes. The peak centered at 880 cm^–1^ was assigned as a ν­(U–O–W) mode, while the peaks centered at 835, 814, 581, and 550 cm^–1^ were assigned as ν­(W–O–W) modes. The bands centered at 434 and 361 cm^–1^ were assigned as δ­(W–O–W/W=O/U–O–W) modes, and the remaining features centered at 232, 212, 179, 164, and 142 cm^–1^ were assigned as POM deformation modes. The full vibrational mode assignments for the Raman spectra of **1**–**8** can be found in Table 4.

**Table 4:** Raman Mode Assignments for Complexes **1**–**8**

complexes	ν(W=O) modes (cm^–1^)	ν(U–O–W) modes (cm^–1^)	ν(W–O–W) modes (cm^–1^)	δ(W–O–W/W=O/U–O–W) modes(cm^–1^)	POM deformation modes (cm^–1^)
UW10 Li (**1**)	950.55, 982.33	886.97	562.53	356.81, 426.40, 480.03	140.23, 174.52, 214.35
UW10 Li 2 (**1b**)	950.57, 982.72	889.20	565.58, 584.91	356.72, 427.06, 480.33	140.06, 175.25, 214.04
UW10 LiF (**6**)	938.95, 952.30, 969.97, 991.32	887.97	560.50, 584.52, 800.99, 834.25	347.37, 365.47, 430.69	140.10, 170.23, 211.76, 238.62
UW10 Na (**2**)	936.59, 949.22, 969.45	879.97	549.25, 581.24, 814.05, 835.55	360.88, 433.75	142.36, 163.62, 178.57, 211.75, 232.24
UW10 K (**3**)	944.52, 969.32	884.56	552.18, 562.71, 799.95, 835.49	367.06, 440.48	139.17, 172.90, 218.75
UW10 KF (**7**)	935.91, 962.94	882.16	552.41, 585.25, 795.28, 835.20	359.72, 371.35, 417.20, 439.73	145.54, 175.01, 219.94
UW10 Rb (**4**)	941.14, 963.22	880.81	551.19, 562.35, 803.48, 833.64	351.49, 371.32, 440.02, 492.70	104.42, 142.96, 172.06, 218.93, 323.62
UW10 Cs (**5**)	927.46, 955.67, 976.41	879.44	554.51, 799.82, 830.47	363.15, 372.30, 442.49	102.92, 145.29, 170.28, 217.13, 228.46
UW10 CsF (**8**)	920.64, 934.92, 959.44	877.97	548.29, 580.46, 796.36, 826.15	363.33, 371.44, 440.61	101.68, 145.97, 167.58, 175.10, 217.52, 241.54

The Raman spectra for complexes **1** and **3**–**8** (Figures S29–S31 and S33–S37, Supporting Information) display different numbers of peaks compared to the spectrum for **2**; however, the major features are in identical regions, Figure 6. The Raman spectrum of **1**, Li_5_Na_3_[UW_10_], includes five fewer peaks than **2**, Figure S29 (Supporting Information). The peaks centered at 982 and 951 cm^–1^ were assigned as ν­(W=O) modes, while peaks centered at 887 and 563 cm^–1^ were assigned as ν­(U–O–W) and ν­(W–O–W) modes, respectively. Peaks centered at 480, 426, and 357 cm^–1^ were assigned as δ­(W–O–W/W=O/U–O–W) modes, and the remaining peaks centered at 214, 175, and 140 cm^–1^ were assigned as POM deformation modes. Complex **1b** has an almost identical Raman spectrum to that of **1** with one notable difference at 585 cm^–1^ that we assign as an extra ν­(W–O–W) stretch, Figures S29 and S30 (Supporting Information). The similarity between the Raman spectra of the two polymorphs is surprising due to the differences in crystallographic unit cells, Table 1 and Table S1 (Supporting Information), and the distinctions between the FIR spectra of **1** and **1b**, Figures S20 and S21 (Supporting Information). The source of this similarity could be decomposition of less stable **1** to more favorable **1b** due to dehydration of the lattice when the crystals are taken out of the mother liquor during the Raman data collection process. This dehydration happens in a matter of minutes for **1**–**8** and has been observed in our previous work focusing on LnW_10_ complexes and in the literature as well.^15,53^

The Raman spectrum of **3**, K_4_Na_4_[UW_10_], includes one less ν­(W=O) mode and two less features in the POM deformation region compared to **2** and 12 peaks overall, Figure S33 (Supporting Information). The peaks centered at 969 and 945 cm^–1^ were assigned as ν­(W=O) modes. The peak centered at 885 cm^–1^ was assigned as a ν­(U–O–W) mode, while the peaks centered at 835, 800, 563, and 552 cm^–1^ were assigned as ν­(W–O–W) modes. Peaks centered at 440 and 367 cm^–1^ were assigned as δ­(W–O–W/W=O/U–O–W) modes, and the remaining peaks centered at 219, 173, and 139 cm^–1^ were assigned as POM deformation modes. The Raman spectrum of **4**, Rb_6_Na_2_[UW_10_], exhibits one less feature in the ν­(W=O) region and two more features in the δ­(W–O–W/W=O/U–O–W) region compared to **2** and includes 16 total peaks, Figure S35 (Supporting Information). The stretches centered at 963 and 941 cm^–1^ were assigned as ν­(W=O) modes, and the peak centered at 881 cm^–1^ was assigned as a ν­(U–O–W) mode. Stretches centered at 834, 803, 562, and 551 cm^–1^ were assigned as ν­(W–O–W) modes, while the peaks centered at 493, 440, 371, and 351 cm^–1^ were assigned as δ­(W–O–W/W=O/U–O–W) modes. The remaining peaks centered at 324, 219, 172, 143, and 104 cm^–1^ were assigned as POM deformation modes. The Raman spectrum of **5**, Cs_5.5_Na_2.5_[UW_10_], includes one less feature in the ν­(W–O–W) region and one more feature in the δ­(W–O–W/W=O/U–O–W) region compared to **2**, and both spectra feature 15 peaks, Figures S32 and S36 (Supporting Information). The stretches centered at 976, 956, and 927 cm^–1^ were assigned as ν­(W=O) modes, and the peak centered at 879 cm^–1^ was assigned as a ν­(U–O–W) mode. The peaks centered at 830, 800, and 555 cm^–1^ were assigned as ν­(W–O–W) modes, while the peaks centered at 442, 372, and 363 cm^–1^ were assigned as δ­(W–O–W/W=O/U–O–W) modes. The remaining peaks centered at 228, 217, 170, 145, and 103 cm^–1^ were assigned as POM deformation modes.

The Raman spectrum for **6**, Li_8_[UW_10_], displays one additional feature in both ν­(W=O) and δ­(W–O–W/W=O/U–O–W) regions and one less stretch in the POM deformation mode region compared to **2** and 16 peaks overall, Figure S31 (Supporting Information). The bands centered at 991, 970, 952, and 939 cm^–1^ were assigned as ν­(W=O) modes, and the peak centered at 888 cm^–1^ was assigned as a ν­(U–O–W) mode. The peaks centered at 834, 801, 584, and 561 cm^–1^ were assigned as ν­(W–O–W) modes, while the stretches centered at 431, 365, and 347 cm^–1^ were assigned as δ­(W–O–W/W=O/U–O–W) modes. The remaining peaks centered at 239, 212, 170, and 140 cm^–1^ were assigned as POM deformation modes. The Raman spectrum of **7**, K_8_[UW_10_], includes one less feature in the ν­(W=O) region, one more feature in the δ­(W–O–W/W=O/U–O–W) region, and two less features in the POM deformation region compared to **2** and contains 14 assignable peaks, Figure S34 (Supporting Information). The peaks centered at 963 and 936 cm^–1^ were assigned as ν­(W=O) modes, and the peak centered at 882 cm^–1^ was assigned as a ν­(U–O–W) mode. The stretches centered at 835, 795, 585, and 552 cm^–1^ were assigned as ν­(W–O–W) modes, while the peaks centered at 440, 417, 371, and 360 cm^–1^ were assigned as δ­(W–O–W/W=O/U–O–W) modes. The remaining peaks centered at 220, 175, and 146 cm^–1^ were assigned as POM deformation modes. Finally, the Raman spectrum for **8**, Cs_8_[UW_10_], exhibits one more feature in both the δ­(W–O–W/W=O/U–O–W) and POM deformation regions compared to **2** and includes 17 peaks overall, Figure S37 (Supporting Information). The peaks centered at 959, 935, and 921 cm^–1^ were assigned as ν­(W=O) modes, and the peak centered at 878 cm^–1^ was assigned as a ν­(U–O–W) mode. The stretches centered at 826, 796, 580, and 548 cm^–1^ were assigned as ν­(W–O–W) modes, and the peaks centered at 441, 371, and 363 cm^–1^ were assigned as δ­(W–O–W/W=O/U–O–W) modes. The remaining peaks centered at 242, 218, 175, 168, 146, and 102 cm^–1^ were assigned as POM deformation modes.

In general, the Raman spectra of **1**–**8** are more alike compared to FIR spectra for the same species; however, the differences in the number of peaks suggest there is a distinct vibrational energy splitting regime for each of the complexes that may be related to the composition and packing of the lattice. When we directly compare related species, such as **1**, **1b**, and **6**, we note that the spectra of **1** and **1b** are nearly identical, whereas for **6** we observe extra features in the ν­(W=O) and ν­(W–O–W) regions. These differences may be a result of structural changes for **6**, which features extensive interactions between the Li­(I) cations and the UW_10_ cluster, in contrast to **1**, that could split the degeneracy of the ν­(W=O) and ν­(W–O–W) modes in these complexes, especially for the ν­(W=O) mode as there are abundant interactions between the terminal cluster oxygen atoms and the Li­(I) counterions in **6**. However, if this was the case, we would expect to see more extensive peak splitting in the Raman spectra of **3**–**8** (excluding **6**), rather than the more limited selection of ν­(W=O) stretches we observed (Figures S33–S37, Supporting Information). An alternate explanation is that the ion-pairing interactions observed for larger counterions in **3**–**8** (excluding **6**) can shift the frequency of the ν­(W=O) peak, while the unique interactions provided by the Li­(I) cations in **6** lead to changes in packing symmetry that split the degeneracy of the ν­(W=O) band. Figure S38 (Supporting Information) shows a comparison of ν­(W=O) frequencies versus counterion eIR values wherein a redshift in the ν­(W=O) frequencies as the eIR values increase is noted. This trend confirms that the ν­(W=O) band frequency shifts as a function of counterion size and suggests that interactions between the cluster and the larger counterions may reduce the strength of the ν­(W=O) bond. To further elucidate the origins of vibrational band splitting, computational efforts are required that are outside the scope of this investigation; however, we can confirm that there are overall shifts in the major Raman and FIR features as a function of structural parameters that will be explored further in the next section.

### Structural-Vibrational Correlations

We used qualitative comparisons and partial least squares (PLS) analysis to understand the specific correlations between structural and vibrational features in UW_10_ systems. The structural features evaluated in this study include skew angles (SA), plane angles (PA), plane distances (PD), effective ionic radii (eIR), and average *d*_U–M_ distances, which capture structural characteristics within the first and second coordination spheres. All relevant structural parameters are listed in Table 2. The vibrational bands of interest here are the ν­(WO_5_)_2_, ρ­(UO_8_), ν/ρ­(UO_8_), and δ/ρ­(UO_4_) modes from the FIR and the δ­(W–O–W/W=O/U–O–W), ν­(W–O–W), ν­(U–O–W), and low energy POM deformation modes from the Raman. We will first discuss the correlations found between structural parameters and FIR modes before moving onto analysis featuring Raman modes.

The qualitative comparison of DPs versus the ν­(WO_5_)_2_ mode reveals a general redshift of the ν­(WO_5_)_2_ frequencies as the SAs and PDs increase in value, Figure S39 (Supporting Information). In contrast, the plane angles do not display any clear correlation with the ν­(WO_5_)_2_ frequencies, as shown in Figure S39 (Supporting Information). When the frequencies of ν­(WO_5_)_2_ FIR modes are compared to counterion eIR values and average *d*_U–M_ distances, we note linear increases as both structural parameters increase, Figure 7 and Figure S39 (Supporting Information). There were a couple of outliers in the comparison between ν­(WO_5_)_2_ frequencies and average *d*_U–M_ distance, with complexes **5** and **8** not following the linear trend. PLS analysis on this FIR mode produces two latent variables that account for 89.1% of the variance observed in the ν­(WO_5_)_2_ frequencies, Figures S40 and 41 (Supporting Information). Based on the X- and Y-loading plots, Figure S40 (Supporting Information), counterion eIR and average *d*_U–M_ distance are the two most correlated variables with the ν­(WO_5_)_2_ frequencies, and this quantitative conclusion matches the qualitative observations highlighted earlier.

**Figure 7:**
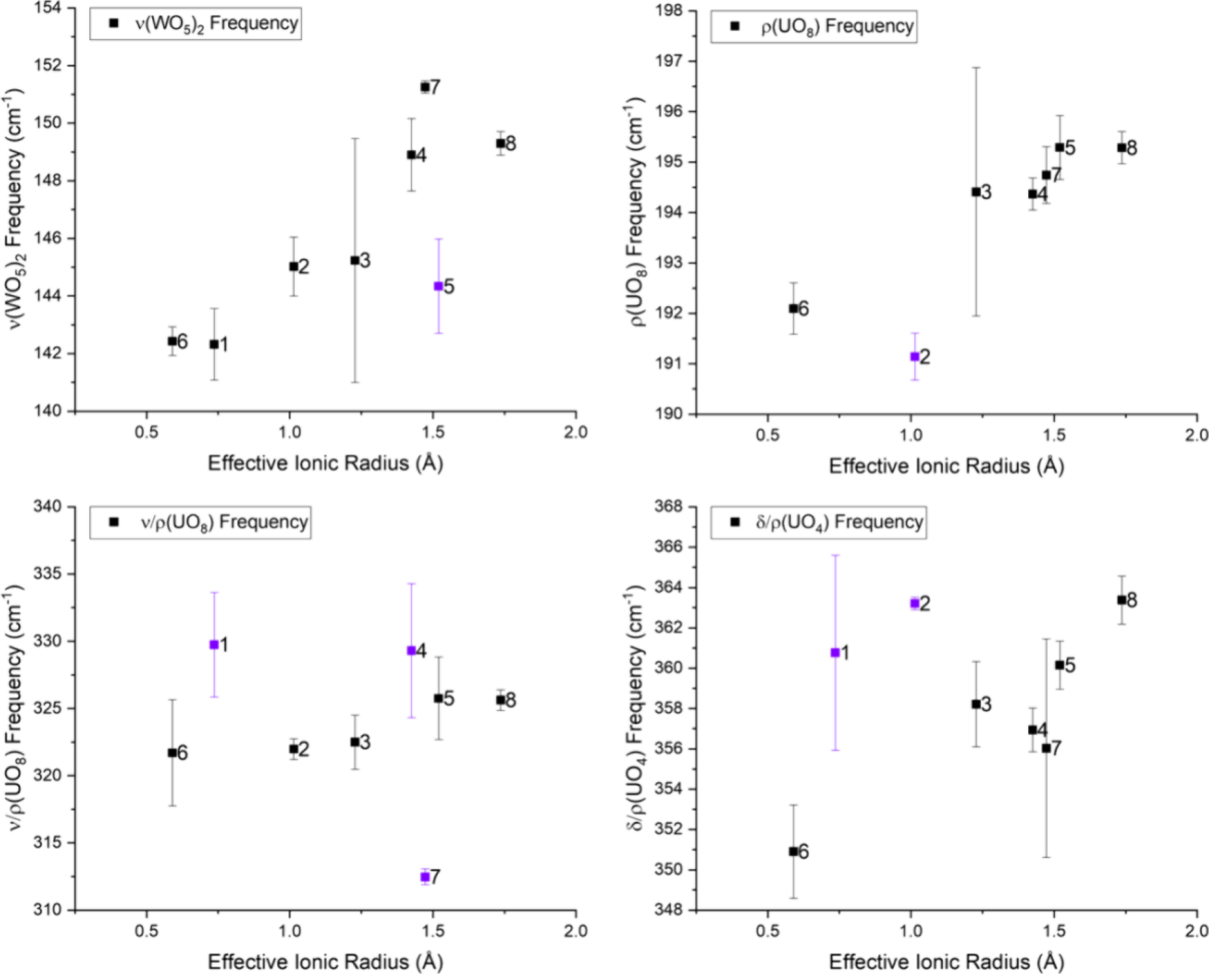
(Top Left) Plot of FIR ν­(WO_5_)_2_ frequencies vs counterion eIR values. (Top Right) Plot of FIR ρ­(UO_8_) frequencies vs counterion eIR values. (Bottom Left) Plot of FIR ν/ρ­(UO_8_) frequencies vs counterion eIR values. (Bottom Right) Plot of FIR δ/ρ­(UO_4_) frequencies vs counterion eIR values. Purple data points indicate outliers to the observed trends.

Qualitative comparisons of FIR ρ­(UO_8_) mode frequencies and structural parameters were also conducted, except for complex **1** where this vibrational stretch was not observed in the corresponding FIR spectrum (Figure 7 and Figure S42 (Supporting Information). The comparisons of ρ­(UO_8_) mode frequencies versus structural distortion parameters reveal general redshifts in the ρ­(UO_8_) frequencies as DP values increase, with the skew angle comparison displaying the most linear correlation, Figure S42 (Supporting Information). In contrast, the plot of ρ­(UO_8_) frequencies versus counterion eIR shows a linear correlation between this FIR mode frequency and the eIR value, with complex **2** being an outlier to this trend, Figure 7. Similarly, when we compare ρ­(UO_8_) frequencies versus average *d*_U–M_ distances, we also observe a blueshift in vibrational frequencies as the average *d*_U–M_ distance increases, Figure S42 (Supporting Information). PLS regression analysis (Figures S43 and S44, Supporting Information) extracted three latent variables out of the independent variables, which managed to account for 96.1% of the variance observed in ρ­(UO_8_) frequencies. This result is consistent with our qualitative analysis, and according to the X- and Y-loading plots, Figure S43 (Supporting Information), the variables with the strongest correlation with the ρ­(UO_8_) frequencies are counterion eIR followed by the average *d*_U–M_ distance. Comparing ν/ρ­(UO_8_) mode frequencies with skew angle and plane angle values reveals redshifts in this stretch as both DP values increase, Figure S45 (Supporting Information). In contrast, the plot of ν/ρ­(UO_8_) frequencies versus plane distance values does not show any correlation, Figure S45 (Supporting Information). When ν/ρ­(UO_8_) mode frequencies are compared with counterion eIR values, a blue shift in vibrational frequencies is noted as eIR values increase, Figure 7, and this trend is also observed in the plot of ν/ρ­(UO_8_) frequencies versus average *d*_U–M_ distances, Figure S45 (Supporting Information). It is worth nothing that the correlations seen in these figures are not strong correlations, and expectedly, the PLS regression analysis for this FIR mode failed to pass cross validation and extract any latent variables from the structural parameters, Figure S46 (Supporting Information). When the cross validation method is bypassed, the PLS model shows that complex plane angles and average *d*_U–M_ distances are the two most correlated variables with this FIR mode. The failure of the PLS regression analysis to build a statistically relevant model supports the idea that there are weak to no correlations between this FIR mode and the investigated structural parameters.

Finally, the comparisons of δ/ρ­(UO_4_) frequencies versus structural DPs revealed that mode stretches are redshifted as DPs values increase, Figure S47 (Supporting Information). In contrast, the qualitative comparison between δ/ρ­(UO_4_) mode frequency and counterion eIR revealed a blueshift in this FIR mode as eIR values increased, Figure 7, while there was no correlation observed when δ/ρ­(UO_4_) mode frequencies and average *d*_U–M_ distances were compared, Figure S47 (Supporting Information). Despite the qualitative correlations, the cross validation tests for the PLS models failed to identify a statistically significant factor out of the independent variables. When the cross validation test was bypassed, complex skew angles and plane angles were the two most correlated variables with this FIR mode, Figure S48 (Supporting Information), yet contributions to mode variance were limited.

Qualitative structural-vibrational comparisons indicated that there were correlations between the distortion and structural parameters and FIR modes, and these were quantitatively supplemented using PLS analysis. The PLS regression models for the ν­(WO_5_)_2_ and ρ­(UO_8_) modes pass cross validation tests and show that counterion eIR and average *d*_U–M_ distances are strongly correlated with these FIR modes. Interestingly, based on these PLS models, structural distortion parameters do not seem to be strongly correlated with FIR modes. When we look at PLS models without the cross validation test results included, structural DPs do appear more relevant, but this may be due to overfitting of regression models; thus, no definitive conclusions can be made about the relevance of structural DPs for modulating FIR vibrational modes. Overall, the qualitative and quantitative correlations that we have identified validate and emphasize the importance of the secondary lattice in modulating the FIR properties for complexes **1**–**8**. In particular, the correlations between counterion eIR and average *d*_U–M_ distance with the FIR modes demonstrate the importance of counterion identity and size as routes to modulate POM vibrational frequencies.

Moving on to the Raman modes, the POM deformation mode frequencies do not display clear correlations with structural DPs based on qualitative comparisons, Figure S49 (Supporting Information); however, comparisons of POM deformation mode frequencies and counterion eIR and average *d*_U–M_ distance do feature linear relationships, Figure 8 and Figure S49 (Supporting Information). The PLS regression model for the POM deformation modes included two latent variables that account for 50.7% of the variance observed in these Raman modes, Figures S50 and S51 (Supporting Information). Based on the X- and Y-loading plots, counterion eIR is the most strongly correlated parameter with the POM deformation mode frequencies followed by skew angles and average *d*_U–M_ distances, Figure S50 (Supporting Information).

**Figure 8:**
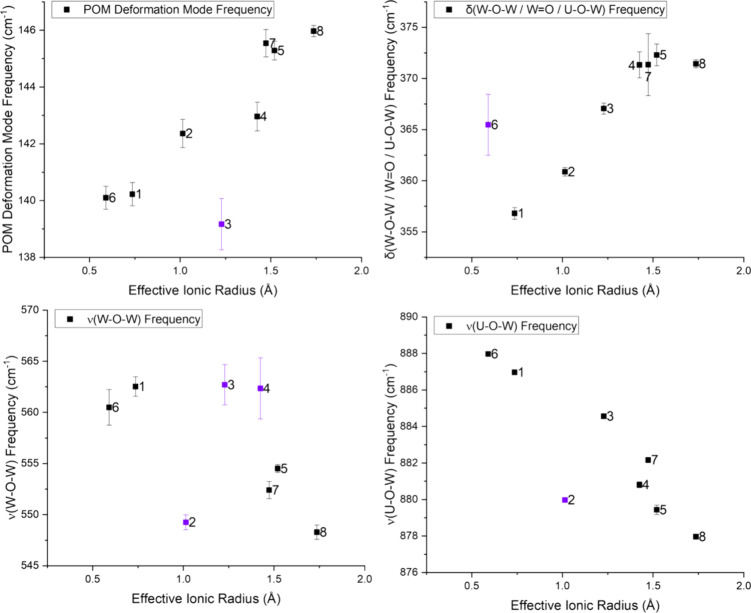
(Top Left) Plot of Raman POM deformation mode frequencies vs counterion eIR values. (Top Right) Plot of Raman (W–O–W/W=O/U–O–W) frequencies vs counterion eIR values. (Bottom Left) Plot of Raman ν­(W–O–W) frequencies vs counterion eIR values. (Bottom Right) Plot of Raman ν­(U–O–W) frequencies vs counterion eIR values. Purple data points indicate outliers to observed trends.

Comparisons of δ­(W–O–W/W=O/U–O–W) Raman frequencies versus structural DPs do not show any clear correlations, Figure S52 (Supporting Information), and similarly, no clear trend can be observed in the plot comparing δ­(W–O–W/W=O/U–O–W) frequencies and average *d*_U–M_ distances, Figure S52 (Supporting Information). In contrast, we observe a blueshift in δ­(W–O–W/W=O/U–O–W) frequencies as the counterion eIR values increase (Figure 8), and the PLS analysis model for this Raman mode extracted two latent variables that account for 91.0% of the variance in this mode frequency, Figures S53 and S54 (Supporting Information). Based on the X- and Y-loading plots, Figure S53 (Supporting Information), the structural parameter most correlated with variance in the δ­(W–O–W/W=O/U–O–W) stretching frequency is the counterion eIR. The plots comparing ν­(W–O–W) Raman frequencies and structural distortion parameters do not show any correlations between these variables, Figure S55 (Supporting Information). Similarly, the plots of ν­(W–O–W) Raman frequencies versus counterion eIR values and average *d*_U–M_ distance do not include any observable correlations, as shown in Figure 8 and Figure S55 (Supporting Information). PLS regression models echo our qualitative observations, as none of the structural parameters were found to be statistically valid for modeling ν­(W–O–W) Raman frequency variance based on a regression model that includes a cross validation test, Figure S56 (Supporting Information). When the cross validation test is bypassed, the resulting PLS model shows skew angles and counterion eIR values to be the correlated with the ν­(W–O–W) frequencies, Figure S56 (Supporting Information). Finally, the plots comparing ν­(U–O–W) Raman frequencies and structural distortion parameters do not show any correlations, as shown in Figure S57 (Supporting Information). The comparison of ν­(U–O–W) Raman frequencies versus counterion eIR values does display an inverse linear relationship between the two variables, with an outlier in complex **2**, Figure 8. This trend is not observed though when comparing ν­(U–O–W) Raman frequencies with average *d*_U–M_ distances, Figure S57 (Supporting Information). PLS analysis was able to generate a model with two latent variables that accounts for 88.1% of the variance in the ν­(U–O–W) frequencies, Figure S58 and S59 (Supporting Information), and based on the X- and Y-loading plots, Figure S58 (Supporting Information), the ν­(U–O–W) frequencies are heavily correlated with counterion eIR values.

Overall, qualitative and PLS analyses show that Raman mode frequencies for complexes **1**–**8** are also modulated by the structural parameters investigated herein. This is especially true for the δ­(W–O–W/W=O/U–O–W) and ν­(U–O–W) stretches, where greater than 80% of the variance in vibrational frequencies for these modes can be accounted for by structural and distortion parameters according to PLS regression analysis. The Raman modes we have focused on display strong correlations with counterion eIR values, similar to FIR modes, and these results further emphasize the ability of secondary sphere elements to modulate vibrational frequencies. The strong correlations observed between the ν­(U–O–W) mode frequencies and counterion eIR values demonstrate the vibrational tunability that is offered by changing second sphere counterions as these species can interact with the UO_8_ moiety to differing extents, which provides a route for modulating Raman frequencies. The findings here compliment and agree with our previous investigation that focused on the LnW_10_Na Lindqvist POM series.^15^ In our recent work, the structural parameters considered were the ionic radius of the Ln­(III) center and structural distortion parameters (skew angles, plane angles, and plane distances), while the vibrational modes we investigated were the ν­(WO_5_)_2_, ρ­(LnO_8_), and ν/ρ­(LnO_8_) modes in the FIR and the δ­(W–O–W/W=O/Ln–O-W) and ν­(Ln–O-W) modes in Raman spectra.^15^ Our results here show that ρ­(UO_8_), δ­(W–O–W/W=O/U–O–W), and ν­(U–O–W) vibrational modes are strongly impacted by counterion eIR values, which may explain why the PLS models from our previous investigation were only able to account for 30% and 51% of the variance observed for the ρ­(LnO_8_) and ν­(Ln–O-W) stretching frequencies, respectively, and no valid PLS model could be constructed for the δ­(W–O–W/W=O/Ln–O-W) Raman mode.^15^ Further, our previous study also showed how the ν/ρ­(LnO_8_) mode is heavily correlated with complex plane angles, and this matches the results observed here for the ν/ρ­(UO_8_) FIR band.^15^

## Conclusions

In this study, eight species of U­(IV)­W_10_ Peacock–Weakley-type lacunary Lindqvist POMs with a range of alkali metal counterions were synthesized and characterized structurally via X-ray diffraction and vibrationally via Raman, MIR, and FIR spectroscopies. This work is the first study to explore U­(IV)­W_10_ complexes since the initial work of Golubev et al. was published nearly 50 years ago,^35^ and the crystal structure for Na_8_[UW_10_] (complex **2**) matches what was published by Golubev and colleagues^35^ and is also isostructural with Ce­(IV) and Th­(IV)­W_10_ analogues. Changing POM counterions to other alkali metals ranging from Li­(I) to Cs­(I) facilitated the synthesis of complexes **1**–**8**, which include the first examples of UW_10_ structures with Li­(I), K­(I), Rb­(I), and Cs­(I). Complexes **3**–**8** (excluding **6**) include K­(I), Rb­(I), and Cs­(I) as counterions and display increased ion-pairing interactions with the UW_10_ cluster, which agrees with the findings of Colliard and Deblonde as well as Zagrebin et al.^18,49^ Specifically, larger counterions (K­(I), Rb­(I), and Cs­(I)) prefer to sit in the belt position surrounding the UW_10_ cluster, which positions these counterions close to the first coordination sphere of the U­(IV) metal center. Notably, the effective symmetry of the U­(IV) center is modulated by the identity and location of the counterions within the lattice, and this was shown through PLS regression analyses, which found that counterion eIR values and average *d*_U–M_ distances could be correlated with changes in structural distortion parameters such as skew and plane angles. These results support our hypothesis that by tuning the secondary sphere around a POM, we can modulate the electronic manifolds of the UW_10_ complex to hopefully realize a U­(IV)-based molecular qubit. The FIR spectra of **1**–**8** show clear distinctions from one another in the form of different major features either being absent or present despite having the same core cluster, and this suggests that some of these modes are likely coupled with lattice phonon modes. In contrast, the Raman spectra of complexes **1**–**8** show mostly identical major features for each of the eight UW_10_ complexes described herein. Qualitative and PLS analyses were used to probe the relationship between structural and vibrational properties for complexes **1**–**8**, which revealed significant correlations between counterion eIR values and vibrational modes that indicate the vibrational manifolds of U­(IV)­W_10_ complexes can be tuned via modulation of the composition and packing of secondary sphere elements. The strong correlations between vibrational modes involving the UO_8_ moiety with counterion eIR values and average *d*_U–M_ distances suggest that the extent of interactions between the UW_10_ cluster and the lattice counterions is driven by the size and location of the ions relative to the cluster, and these results provide the foundation to vastly expand the electronic tunability of Ln and An POM complexes due to the endless combination of secondary sphere elements that can be included with POM clusters. We are currently pursuing magnetic and electron paramagnetic resonance measurements to directly probe the electronic ground states and spin-properties of the UW_10_ species, and overall, this study has clearly demonstrated the importance of secondary sphere elements on the structural and vibrational manifolds of Peacock–Weakley-type lacunary Lindqvist POM complexes. U­(IV) chemistry is also an ideal platform for extending research efforts to other tetravalent actinides, and studies with Pu­(IV) are underway.

## Supplementary Material



## Data Availability

A preprint reflecting a preliminary version of the findings reported here was posted to ChemRxiv with DOI: 10.26434/chemrxiv-2025-mg1dr.

## Notes

MIR spectra of the tungstate starting materials, structural figures for complexes **1**–**8**, crystallographic parameters for polymorph **1b**, tables of interatomic distances for complexes **1–8**, methodological details for determining structural distortion parameters, counterion eIR, and average *d*_U–M_ distances for complexes **1**–**8**, methodological specifics for the PLS regression models, raw and fitted FIR and Raman spectra for complexes **1**–**8**, plots comparing FIR and Raman vibrational modes frequencies and structural parameters, and plots produced by PLS analysis where the relationships between FIR and Raman vibrational modes frequencies and structural distortion parameters were investigated (PDF)
