# Supplementary material for: Delineating the Effects of Counterions on the Structural and Vibrational Properties of U(IV) Lindqvist Polyoxometalate Complexes
Source: Inorg Chem. 2025 May 30;64(23):11380–97. doi: 10.1021/acs.inorgchem.5c00033 (PMC12175120; doi:10.1021/acs.inorgchem.5c00033)
Supplement: Supplementary file 1 [file ic5c00033_si_001.pdf]

Supporting Information

Delineating the Effects of Counterions on the Structural and Vibrational Properties of U(IV) Lindqvist Polyoxometalate Complexes

Primadi J. Subintoro[1] and Korey P. Carter[1*]
[1]Department of Chemistry, University of Iowa, Iowa City, IA 52242, United States
* Corresponding author's e-mail: korey-carter@uiowa.edu

Table of Contents

Mid-IR spectra of Na_2WO_4•$2H_2O$, Cs_2WO_4, and K_2WO_4	**S1**
Asymmetric unit and packing representation of complex **1** ($Li_5Na_3[UW_{10}]$)	**S2**
Table of selected interatomic distances in complexes **1-8**	**S3**
Crystal picture and crystallographic parameters of $UW_{10}Li$ polymorph (**1b**)	**S4**
Asymmetric unit and packing representation of complexes **2** ($Na_8[UW_{10}]$), **3** ($K_4Na_4[UW_{10}]$), **4** ($Rb_6Na_2[UW_{10}]$), **5** ($Cs_{5.5}Na_{2.5}[UW_{10}]$), **6** ($Li_8[UW_{10}]$), **7** ($K_8[UW_{10}]$), and **8** ($Cs_8[UW_{10}]$)	**S5-S12**
Distortion parameter schemes and methodological details	**S13**
Effective ionic radius and average $d_{U\text{-}M}$ methodological details and table of eIR and $d_{U\text{-}M}$ values for complexes **1-8**	**S14-15**
Partial Least Squares (PLS) analysis methodological details	**S16-S17**
PLS analysis plots for UW_{10} structural parameter versus distortion parameter comparisons	**S18-S23**
Fitted far IR spectra of complexes **1-8**	**S24-S32**
Raman spectra of complexes **1-8**	**S33-S41**
Qualitative comparisons and PLS plots of FIR and Raman vibrational mode [$\nu(WO_5)_2$, $\rho(UO_8)$, $\nu/\rho(UO_8)$, $\delta/\rho(UO_4)$, POM deformation mode, δ(W-O-W/W=O/U-O-W), ν(W-O-W), ν(U-O-W)] frequencies versus UW_{10} structural distortion parameters	**S42-S62**
References	**S63**

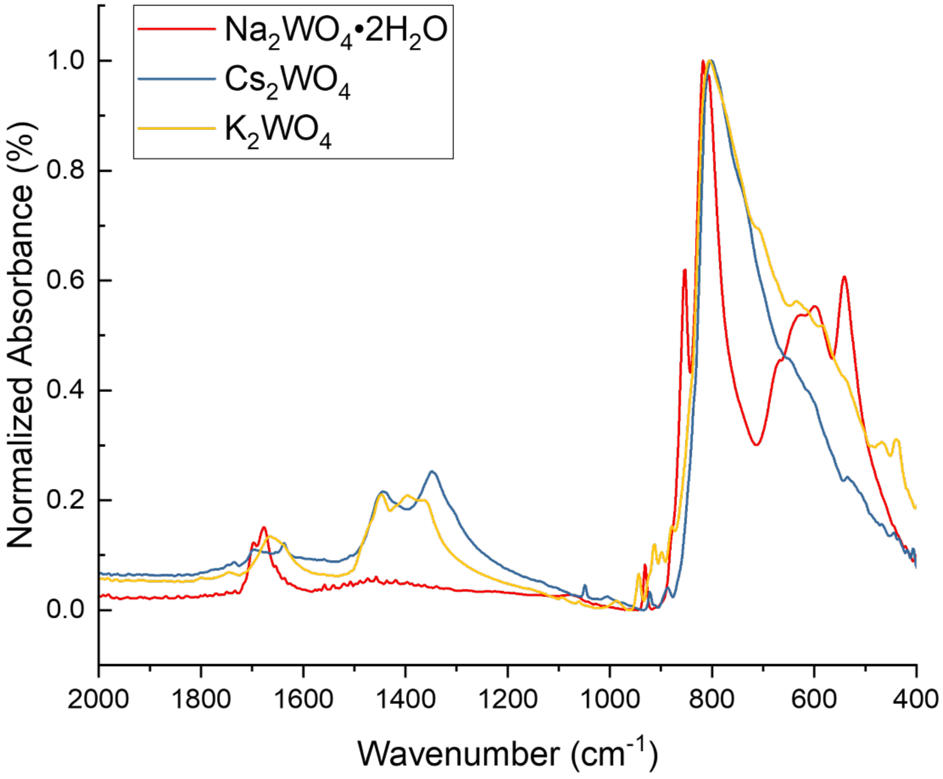

Figure S1. Mid-infrared (MIR) spectra of $Na_2WO_4•2H_2O$, Cs_2WO_4, and K_2WO_4.

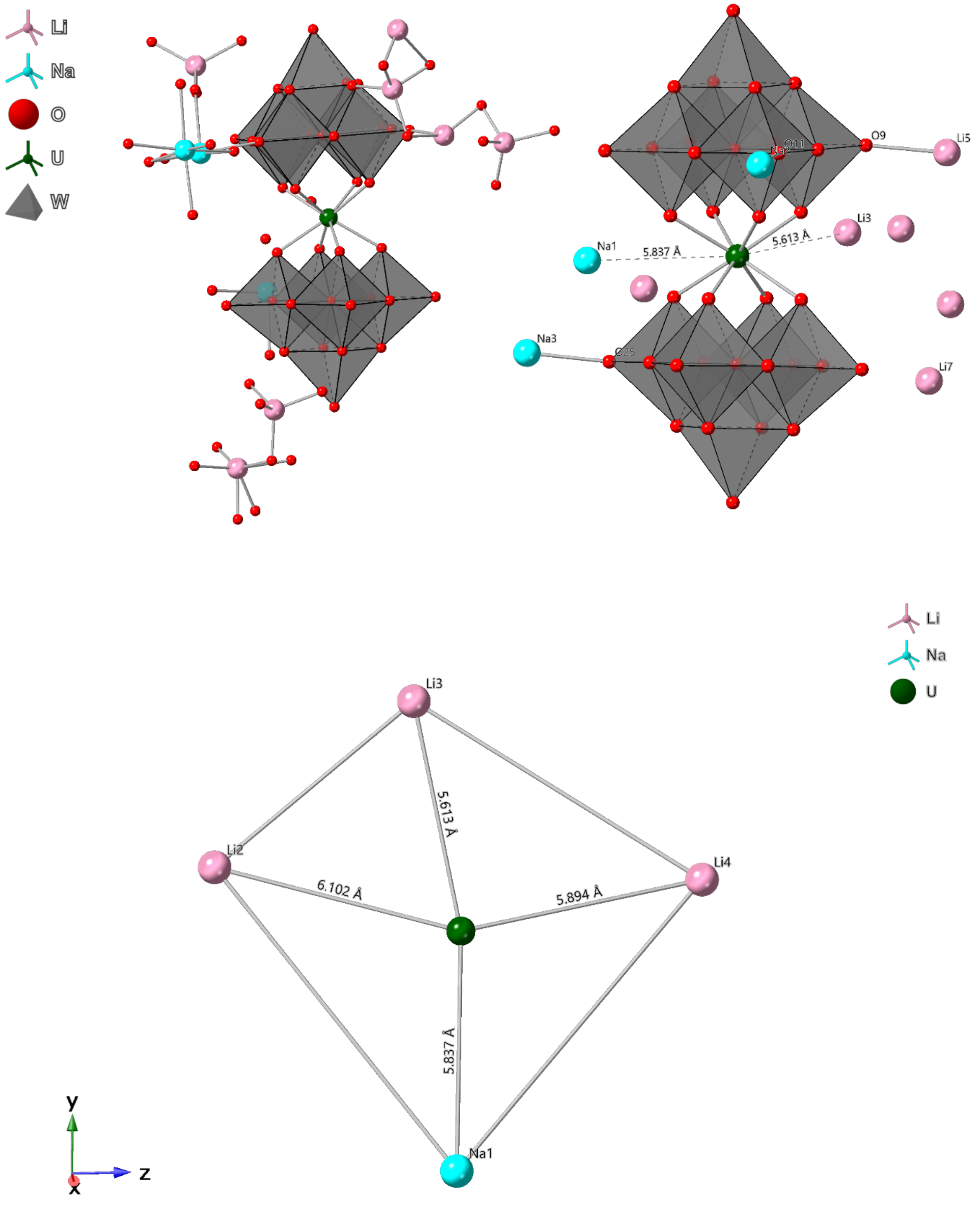

Figure S2. (**Top Left**) Polyhedral representation of the asymmetric unit of **1**. (**Top Right**) Polyhedral representation of the packing of **1** excluding lattice water molecules for clarity. (**Bottom**) Ball and stick representation of the belt area of **1** highlighting alkali metal interactions distances to the U(IV) metal center.

Table S1. Selected interatomic distances in complexes **1-8**

Complex	Interactions	Distance (Å)	Complex	Interactions	Distance (Å)	Complex	Interactions	Distance (Å)
1	**U001-O1**	2.4041(35)		**Rb6A-O3**	2.9578(158)		**U1-O4**	2.3655(59)
	**U001-O2**	2.3339(35)		**Rb6A-O11**	2.9480(176)		**U2-O19**	2.3875(61)
	**U001-O3**	2.3547(36)		**Rb6B-O3**	3.0788(200)		**U2-O20**	2.4006(57)
	**U001-O4**	2.3777(33)		**Rb6B-O10**	3.0562(209)		**U2-O21**	2.3599(66)
	**U001-O19**	2.3617(36)		**Rb6B-O21**	2.8156(204)		**U2-O22**	2.3655(55)
	**U001-O20**	2.3409(36)		**Rb6C-O2**	2.9042(264)		**K1-O9**	2.8304(71)
	**U001-O21**	2.4101(35)		**Rb6C-O21**	3.2504(292)		**K1-O25**	2.7645(68)
	**U001-O22**	2.3825(31)		**Rb6C-O30**	3.0109(270)		**K2-O18**	2.9036(66)
	**Li5-O9**	2.3686(154)		**Rb6D-O1**	3.2778(315)		**K2-O20**	3.0457(57)
	**Na2-O11**	2.4471(42)		**Rb6D-O2**	3.2409(260)		**K3-O11**	2.6862(63)
	**Na3-O25**	2.4211(41)		**Rb6D-O8**	3.0450(260)		**K3-O27**	2.7318(58)
2	**U1-O1**	2.3799(42)		**Rb6D-O22**	2.8409(258)		**K4-O28**	2.7325(56)
	**U1-O2**	2.3716(36)	5	**U1-O1**	2.3870(141)		**K4-O33**	3.1265(60)
	**U1-O3**	2.3697(39)		**U1-O2**	2.3704(117)		**K4-O34**	2.8156(66)
	**U1-O4**	2.3567(39)		**U1-O12**	2.3332(124)		**K5-O31**	2.5824(86)
	**Na2-O9**	2.5046(44)		**U1-O13**	2.3801(192)		**K6-O23**	2.6982(70)
3	**U1-O1**	2.3809(65)		**U1-O14**	2.3539(188)		**K7-O29**	2.9249(67)
	**U1-O2**	2.3463(56)		**Cs1-O18**	3.1298(137)		**K8A-O3**	2.7181(171)
	**U1-O3**	2.4001(62)		**Cs1-O21**	3.0440(98)		**K8B-O3**	2.8743(179)
	**U1-O4**	2.3633(55)		**Cs2A-O2**	2.9606(119)		**K9A-O3**	2.7990(128)
	**U1-O19**	2.3970(55)		**Cs2A-O12**	3.4551(139)		**K9A-O8**	2.8967(171)
	**U1-O20**	2.3777(75)		**Cs2A-O13**	3.3890(95)		**K9B-O2**	2.8509(106)
	**U1-O21**	2.3376(55)		**Cs2A-O18**	3.2357(132)		**K9B-O3**	3.0344(102)
	**U1-O22**	2.3414(57)		**Cs2B-O2**	3.3993(150)		**K9B-O8**	2.8337(123)
	**K1-O11**	2.7382(80)		**Cs2B-O4**	3.3547(206)		**K10A-O7**	2.6871(90)
	**K2-O2**	2.7283(61)		**Cs2B-O13**	3.2605(218)	8	**U1-O1**	2.3507(44)
	**K2-O19**	3.1197(74)		**Cs2C-O4**	3.2667(236)		**U1-O2**	2.3669(41)
	**K2-O20**	2.8875(63)		**Cs2C-O13**	3.4230(253)		**U1-O3**	2.3499(55)
	**K2-O26**	3.0012(62)		**Cs3A-O1**	3.0167(143)		**U1-O4**	2.3603(47)
	**K3-O24**	2.8757(59)		**Cs3A-O14**	3.4199(89)		**U1-O19**	2.3610(55)
	**K3-O33**	2.9156(61)		**Cs3A-O16**	3.2285(144)		**U1-O20**	2.4019(44)
	**K3-O34**	2.9357(58)		**Cs3B-O1**	3.1157(162)		**U1-O21**	2.3801(43)
	**K4-O18**	2.7674(73)		**Cs3B-O12**	3.1733(140)		**U1-O22**	2.3943(39)
	**Na4A-O7**	2.3096(111)		**Cs4-O3**	3.1525(128)		**Cs1-O26**	3.1705(52)
	**Na4B-O7**	2.6172(342)		**Cs4-O6**	3.3262(124)		**Cs2-O3**	3.0233(45)
4	**U1-O1**	2.3818(161)	6	**U1-O1**	2.3870(63)		**Cs2-O21**	3.1404(53)
	**U1-O2**	2.3734(148)		**U1-O2**	2.3803(74)		**Cs2-O28**	3.3091(58)
	**U1-O3**	2.3705(146)		**U1-O3**	2.3502(64)		**Cs3-O11**	3.0794(63)
	**U1-O4**	2.3735(147)		**U1-O4**	2.4109(72)		**Cs5-O18**	2.9811(59)
	**U1-O19**	2.3780(159)		**U1-O19**	2.3170(73)		**Cs6-O23**	3.0277(61)
	**U1-O20**	2.3638(158)		**U1-O20**	2.3192(73)		**Cs7-O34**	3.0585(55)
	**U1-O21**	2.3680(169)		**U1-O21**	2.4082(71)		**Cs8-O27**	2.9620(54)
	**U1-O22**	2.3384(150)		**U1-O22**	2.3580(76)		**Cs8-O19**	3.2365(46)
	**Rb1-O30**	3.0084(184)		**Li1-O4**	2.0271(291)		**Cs8-O22**	3.4481(45)
	**Rb1-O33**	3.1781(160)		**Li1-O22**	2.0135(278)		**Cs8-O30**	3.1367(44)
	**Rb1-O34**	3.0109(162)		**Li3-O8**	1.9363(209)			
	**Rb2-O36**	2.9317(158)		**Li4-O12**	1.9727(402)			
	**Rb3-O4**	2.7586(142)		**Li5-O35**	2.1336(284)			
	**Rb3-O19**	3.0440(163)		**Li6-O33**	1.9653(235)			
	**Rb3-O26**	3.1944(166)		**Li7-O23**	2.0277(199)			
	**Rb4-O6**	3.0859(164)		**Li8-O17**	2.0167(282)			
	**Rb4-O15**	3.2913(171)	7	**U1-O1**	2.3509(53)			
	**Rb4-O16**	2.9671(157)		**U1-O2**	2.3732(59)			
	**Rb5-O28**	2.9317(147)		**U1-O3**	2.3907(58)			

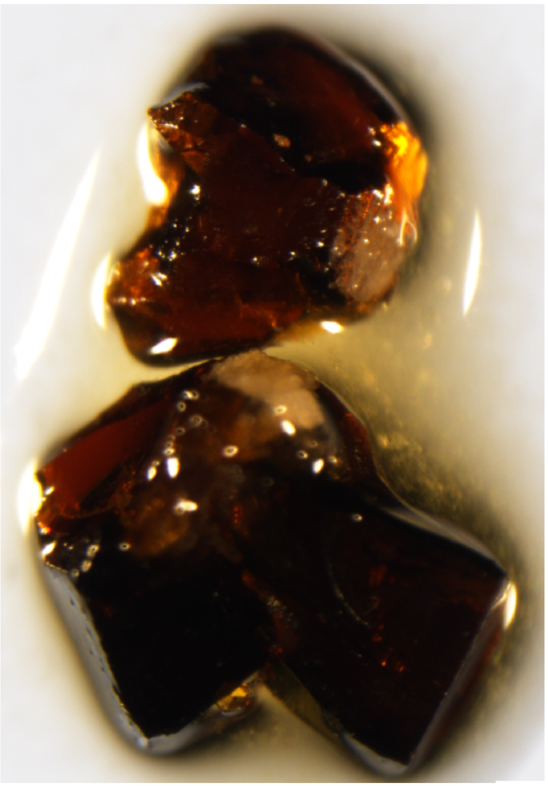

Figure S3. (**Left**) Picture of $UW_{10}Li$ Polymorph (**1b**).

Table S2. Unit cell parameters for **1b**.

	$UW_{10}Li$ (**1b**)
SG	$P6_5$
a(Å)	10.8017 (4)
b(Å)	10.8017 (4)
c(Å)	78.2680 (5)
α	90
β	90
γ	120
V(Å^3)	7908.6 (7)
Temp (K)	100 (2)
Wavelength	0.71703

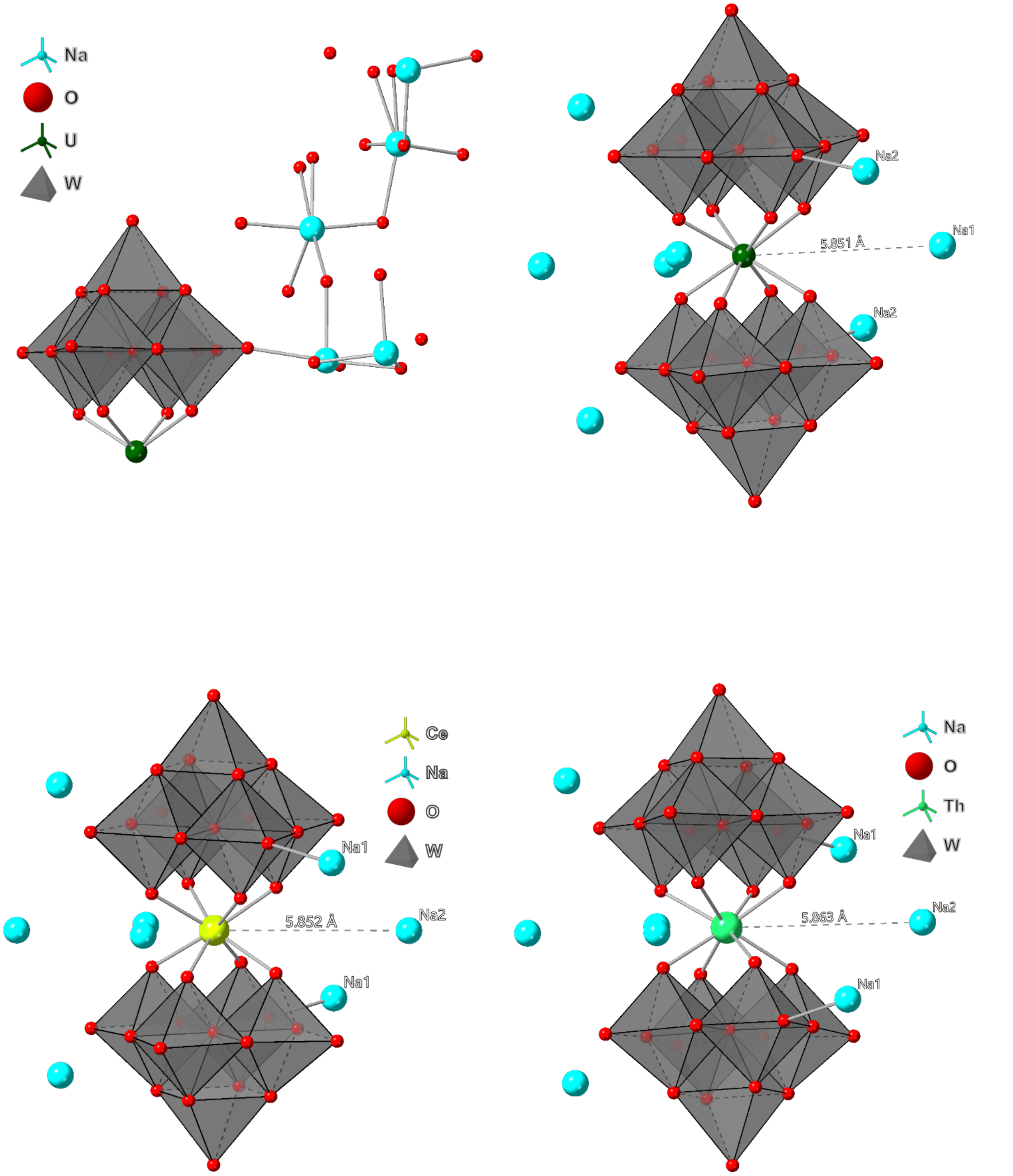

Figure S4. (**Top Left**) Polyhedral representation of the asymmetric unit of **2**. (**Top Right**) Polyhedral representation of the packing of **2** excluding lattice water molecules for clarity. (**Bottom**) Polyhedral representations of Na_8CeW_{10} (left) and Na_8ThW_{10} (right) POM complexes and lattice counterions for comparison.

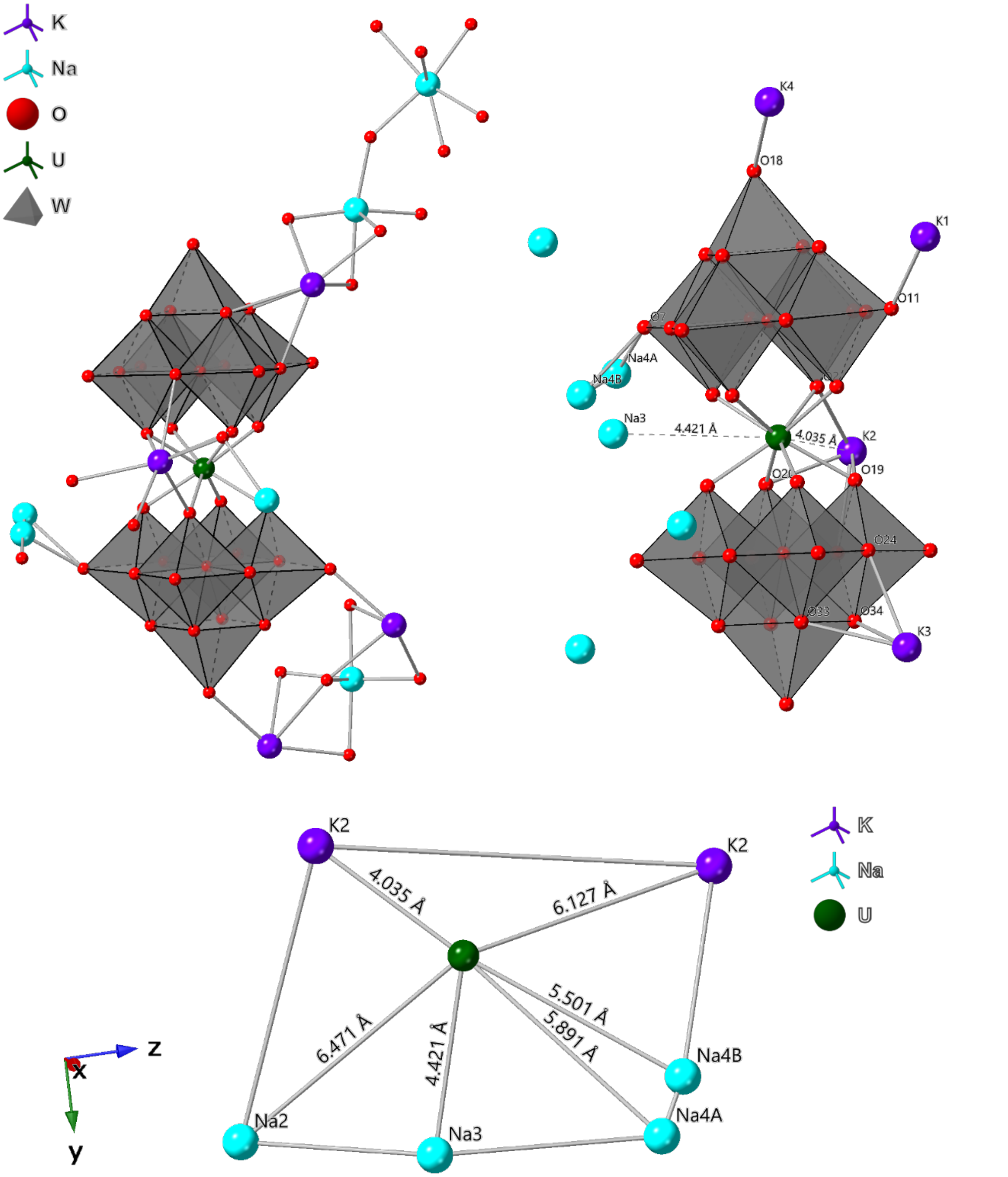

Figure S5. (**Top Left)** Polyhedral representation of the asymmetric unit of **3**. **(Top Right)** Polyhedral representation of the packing of **3** excluding lattice water molecules for clarity. (**Bottom)** Ball and stick representation of the belt area of **3** highlighting alkali metal interactions distances to the U(IV) metal center.

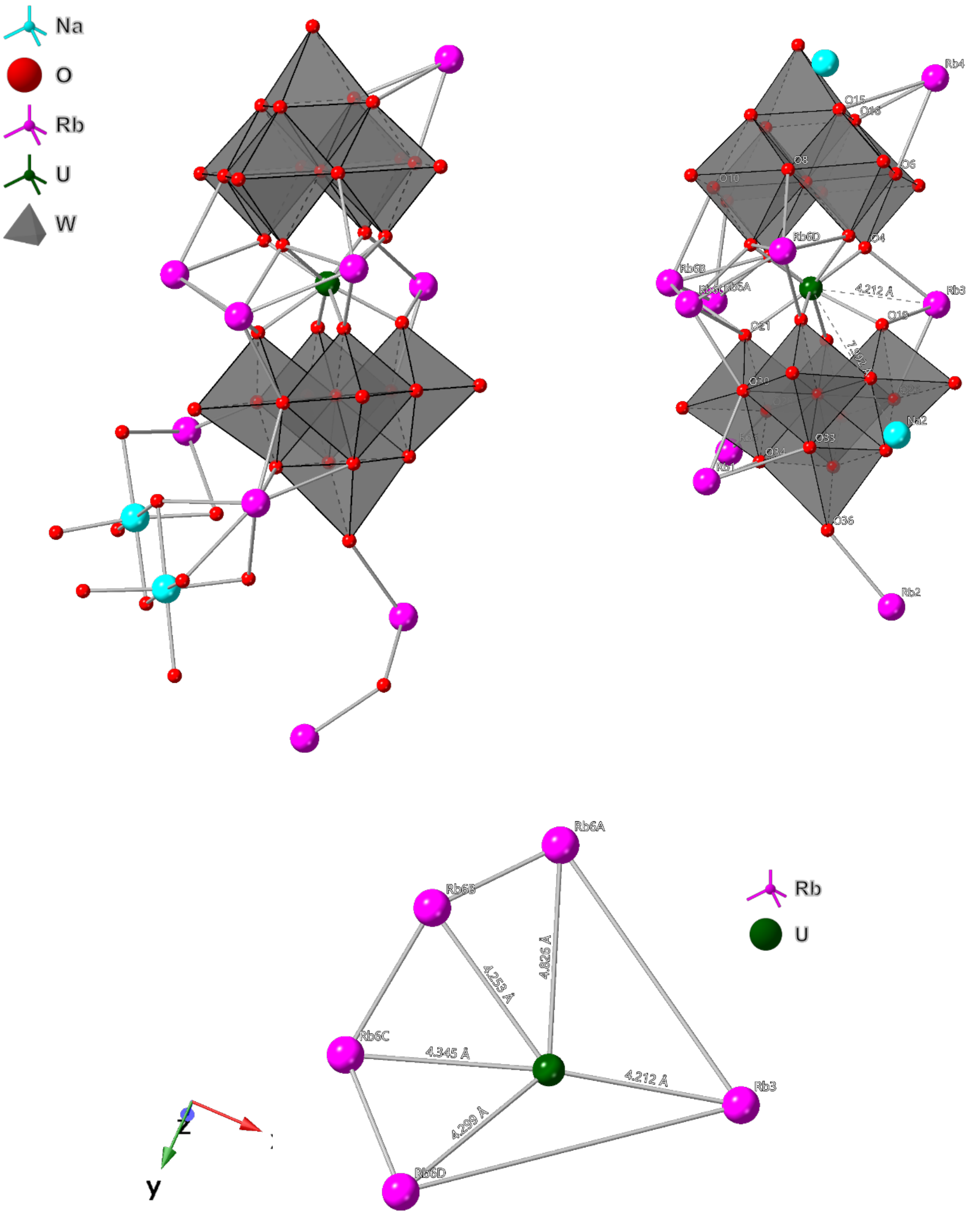

Figure S6. (Top Left) Polyhedral representation of the asymmetric unit of **4**. **(Top Right)** Polyhedral representation of the packing of **4** excluding lattice water molecules for clarity. (**Bottom)** Ball and stick representation of the belt area of **4** highlighting alkali metal interactions distances to the U(IV) metal center.

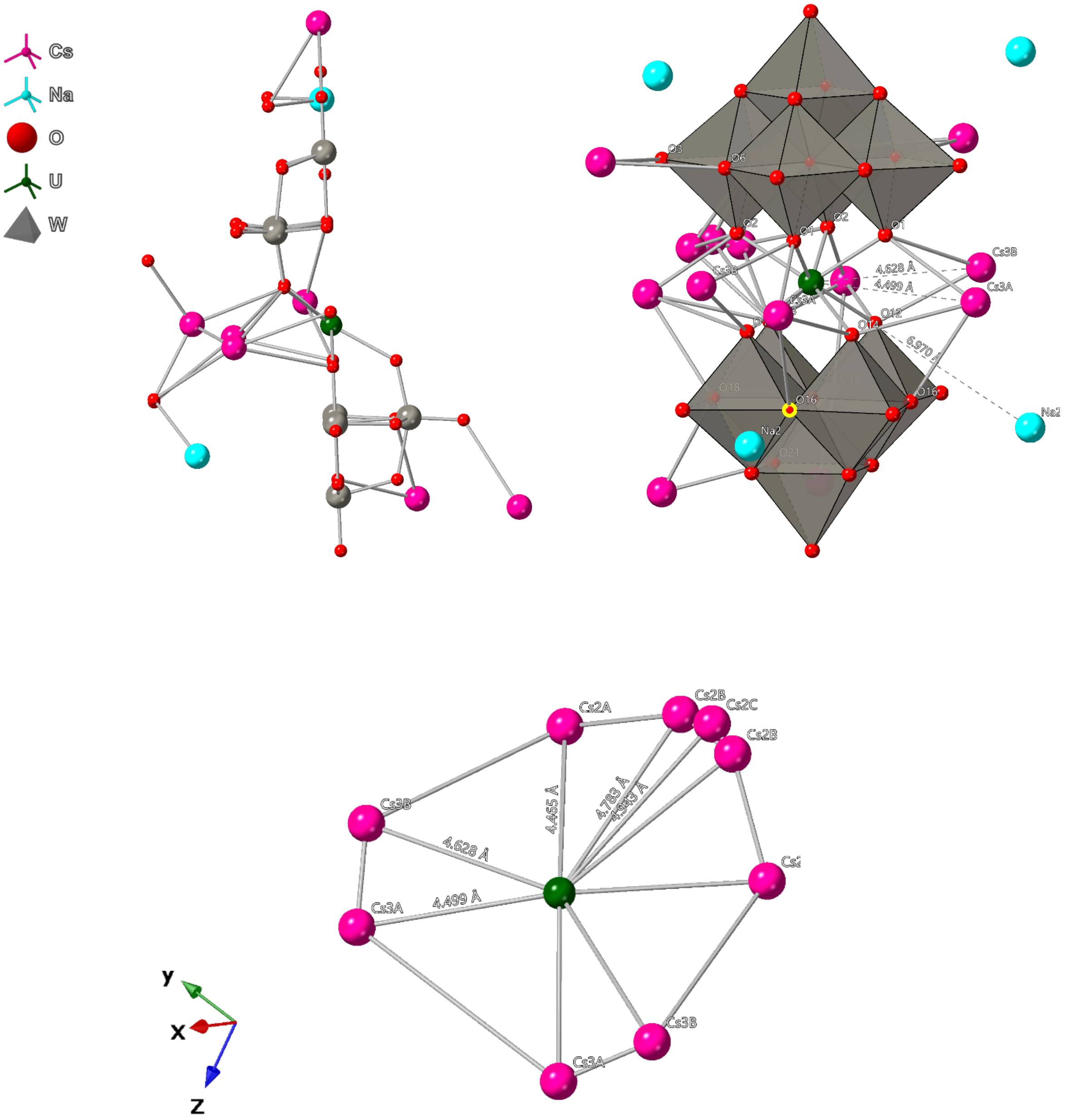

Figure S7. (**Top Left)** Ball and stick representation of the asymmetric unit of **5**. **(Top Right)** Polyhedral representation of the packing of **5** excluding lattice water molecules for clarity. (**Bottom)** Ball and stick representation of the belt area of **5** highlighting alkali metal interactions distances to the U(IV) metal center.

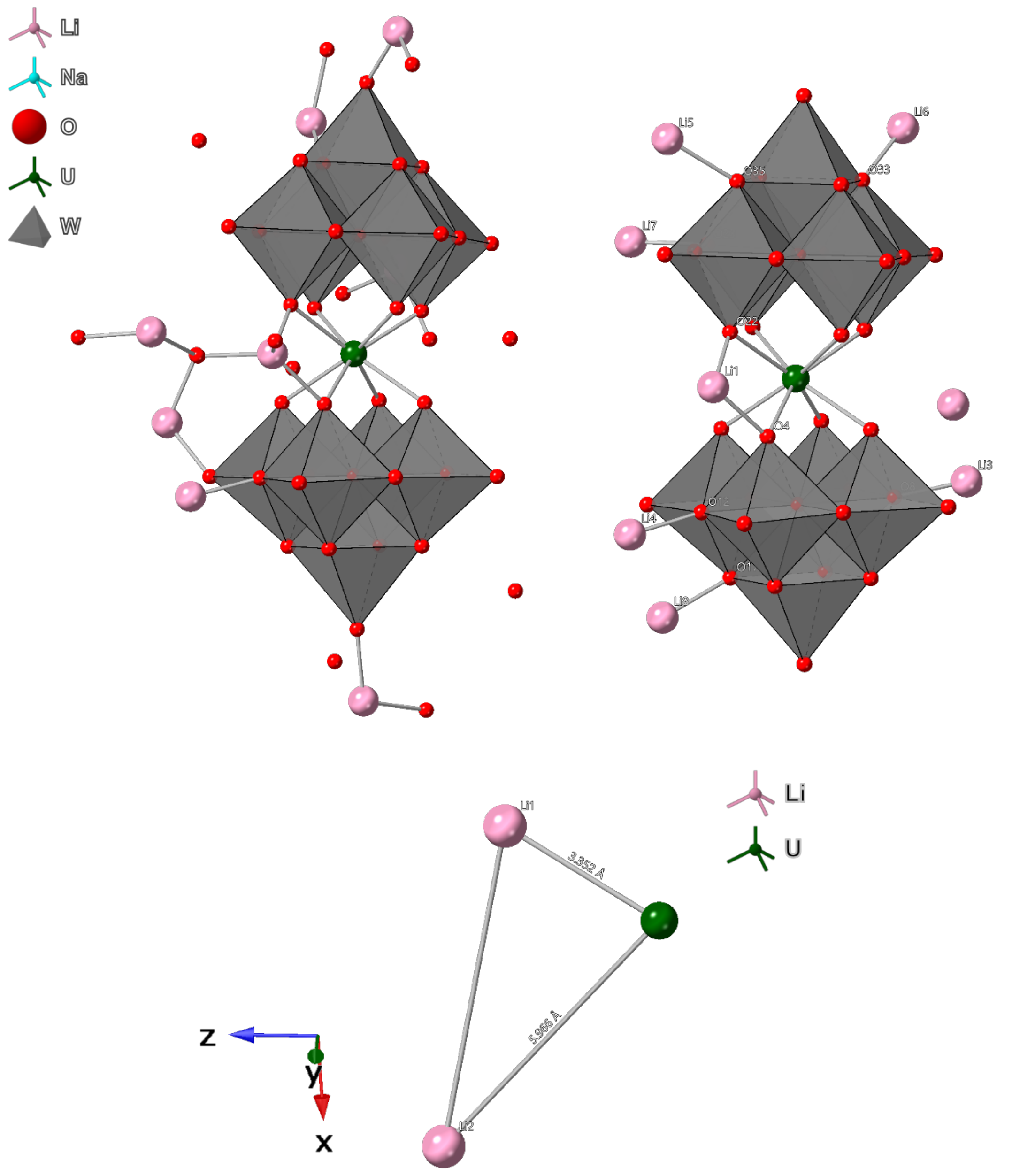

Figure S8. (**Top Left)** Polyhedral representation of the asymmetric unit of **6**. **(Top Right)** Polyhedral representation of the packing of **6** excluding lattice water molecules for clarity. (**Bottom)** Ball and stick representation of the belt area of **6** highlighting alkali metal interactions distances to the U(IV) metal center.

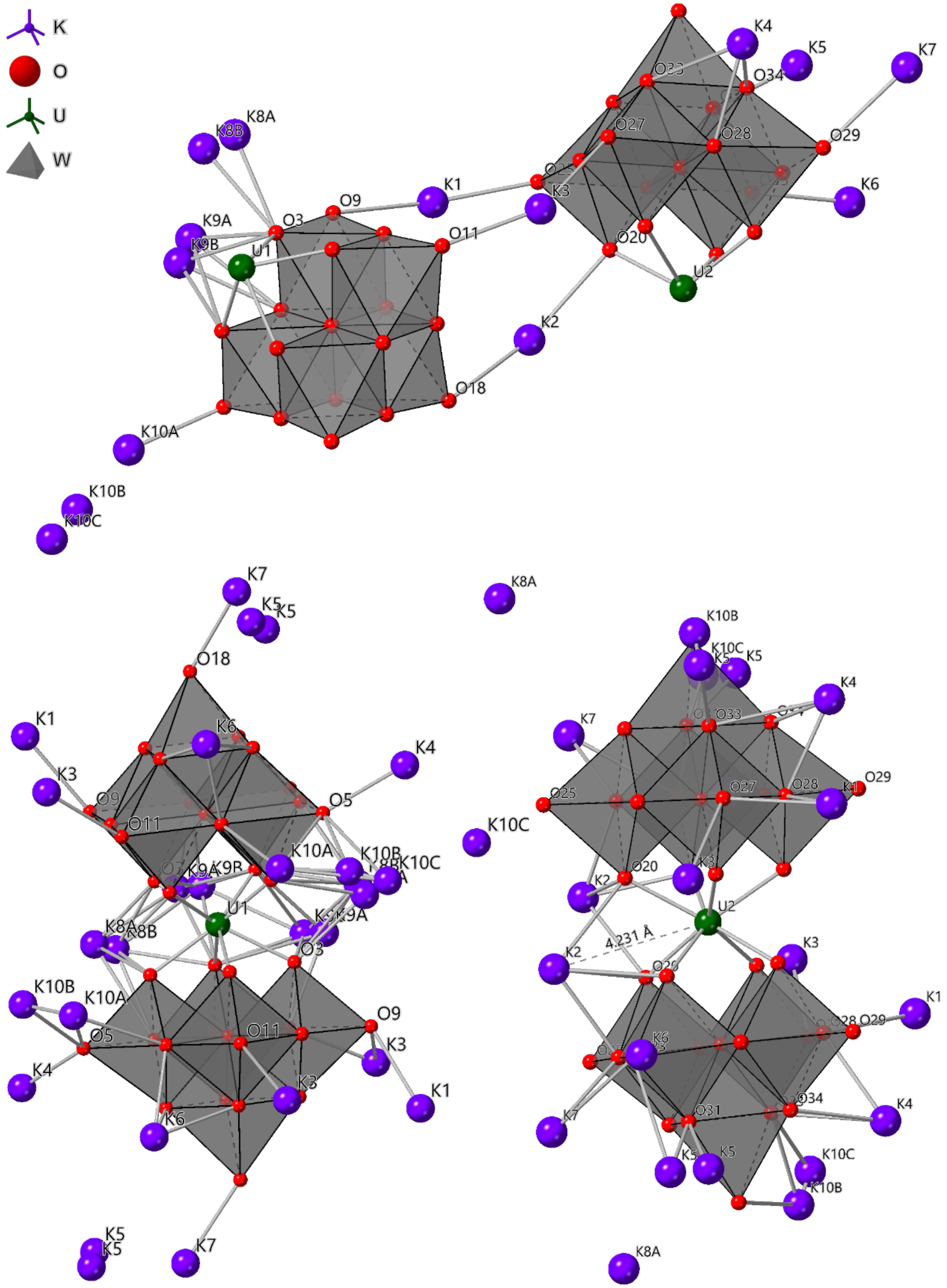

Figure S9. (Top) Polyhedral representation of the asymmetric unit of **7**. (**Bottom**) Polyhedral representation of the packing of the two unique UW_{10} moieties in **7** excluding the lattice water molecules for clarity.

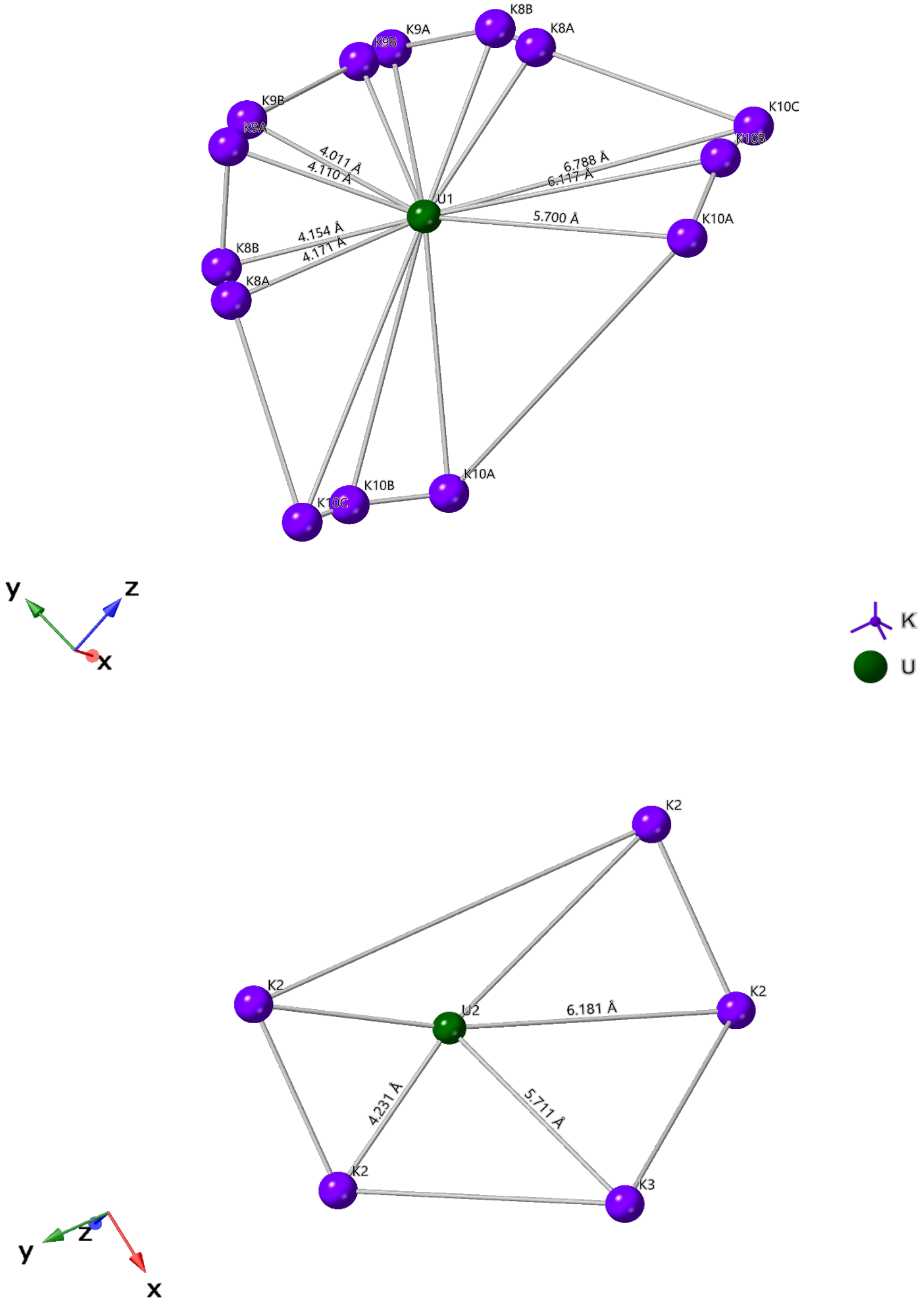

Figure S10. Ball and stick representation of the belt area of the two unique UW_{10} moieties in **7** highlighting alkali metal interactions distances to the U(IV) metal center.

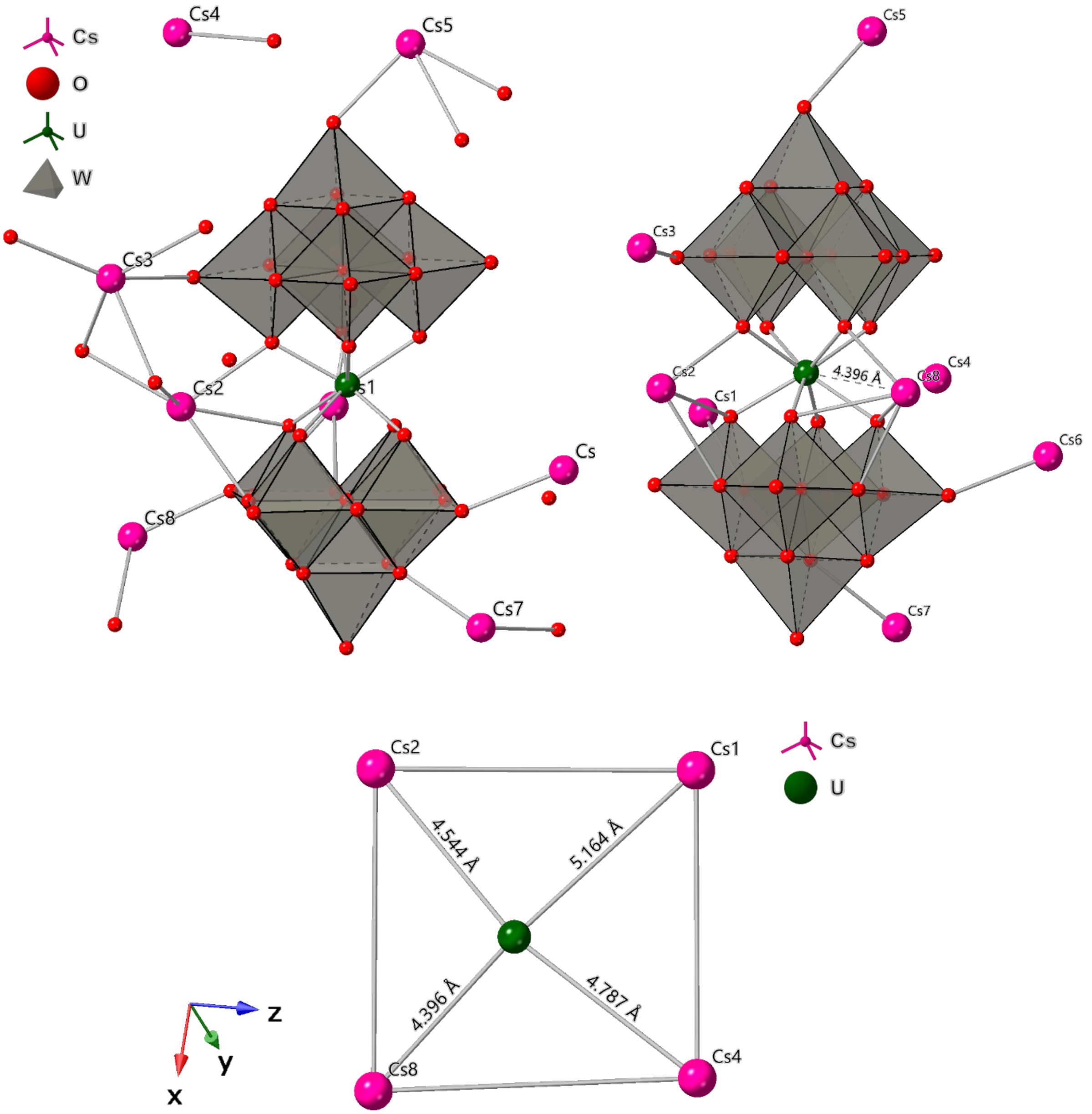

Figure S11. (**Top Left)** Polyhedral representation of the asymmetric unit of **8**. **(Top Right)** Polyhedral representation of the packing of **8** excluding lattice water molecules for clarity. (**Bottom)** Ball and stick representation of the belt area of **8** highlighting alkali metal interactions distances to the U(IV) metal center.

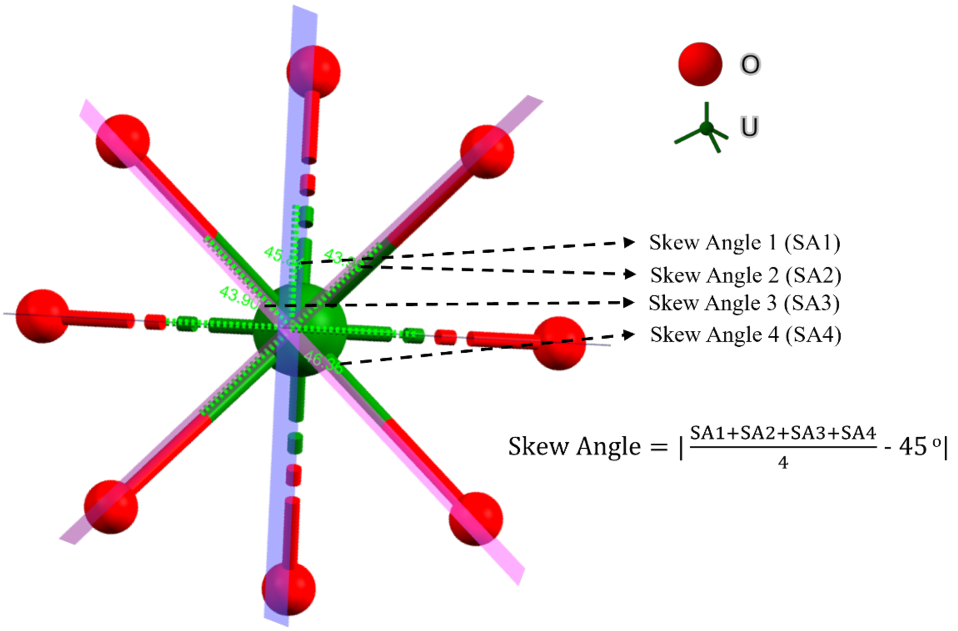

Figure S12. Top-down view of ball and stick representation of the UO_8 moiety along with calculated planes used to determine skew angle values.

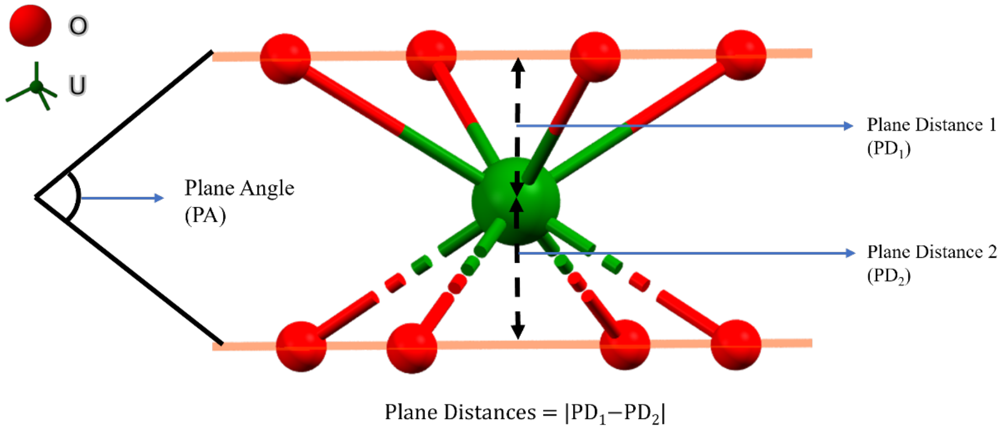

Figure S13. Side view of ball and stick representation of the UO_8 moiety along with calculated planes used to determine plane angle and distance values.

Methodology for acquiring distortion parameters. The skew angle (SA) is calculated by measuring the angle between planes (pink planes) constructed out of the metal center and two crossing oxygen from the top coordinating plane and the metal center with the planes (purple planes) constructed out of the same oxygens and metal center but from the bottom coordinating plane (**Figure S12)**. This method produces four different SAs which are then subtracted from/with 45 ^{o} and averaged to produce the final SA values. Planes are generated and measurements are done in the Mercury software package. The plane angle (PA) is the angle between the two coordinating planes (orange planes) presented in **Figure S13**. The plane distance (PD) is the difference in distances between the top coordinating plane with the metal center and the bottom coordinating plane with the metal center (**Figure S13)**.

Equation S1.

$$\text{Effective Ionic Radius (eIR)} = \sum \frac{N * cMIR}{N}$$

N = Number of counterion
cMIR* = Ionic radius of the counterion with matching coordination number

*All ionic radius (IR) values are obtained from Shannon.[1] When a counterion possesses a coordination number that is not listed from Shannon the ionic radii table, the IR of the closest available coordination number for the counterion is used.[1] For atoms disordered over multiple position, the highest coordination number is used.

Table S3. Crystallographic data and list of ionic radii obtained from Shannon[1] for calculation of effective ionic radii (eIR) values and average $d_{U\text{-}M}$ distances.

Complex	Counterion Identity	Coordination Number to Oxygen	$d_{U\text{-}M}$ (Å)	average $d_{U\text{-}M}$ (Å)	Effective Ionic Radius (Å)
1	Li1	4	7.764	6.396	0.736
	Li2	4	6.102	6.412^{Li}	
	Li3	6	5.613	6.358^{Na}	
	Li4	4	5.894		
	Li5	3	6.426		
	Li6	4	6.391		
	Li7	2	6.697		
	Na1	6	5.837		
	Na2	6	6.634		
	Na3	6	6.604		
2	Na1	6	5.851	6.494	1.014
	Na2	6	6.56		
	Na3	6	7.494		
	Na4	6	6.604		
	Na5	4	5.96		
3	K1	9	7.027	6.293	1.228
	K2	8	4.035	6.189^{K}	
	K3	9	6.909	6.375^{Na}	
	K4	8	6.786		
	Na1	5	8.003		
	Na2	7	5.575		
	Na3	1	4.420		
	Na4A	4	5.891		
	Na4B	4	5.501		
	Na5	4	8.182		
4	Rb1	9	7.058	6.531	1.425
	Rb2	7	6.533	6.010^{Rb}	
	Rb3	3	4.212	8.102^{Na}	
	Rb4	8	7.136		
	Rb5	5	6.674		
	Rb6A	3	4.826		
	Rb6B	4	4.253		
	Rb6C	4	4.345		
	Rb6D	4	4.299		
	Na1	6	8.212		
	Na2	6	7.992		
5	Cs1	8	7.155	6.417	1.520
	Cs2A	9	4.465	5.802^{Cs}	
	Cs2B	7	4.784	7.646^{Na}	
	Cs2C	8	4.943		
	Cs3A	7	4.499		
	Cs3B	9	4.628		
	Cs4	9	6.763		
	Na1	6	8.323		
	Na2	N/A	6.970		

Counterion with CN	Ionic Radius (Å)
4 CN Li	0.59
6 CN Li	0.76
8 CN Li	0.92
4 CN Na	0.99
5 CN Na	1
6 CN Na	1.02
7 CN Na	1.12
8 CN Na	1.18
9 CN Na	1.24
10 CN Na	1.39
4 CN K	1.37
6 CN K	1.38
7 CN K	1.46
8 CN K	1.51
9 CN K	1.55
10 CN K	1.59
12 CN K	1.64
6 CN Rb	1.52
7 CN Rb	1.56
8 CN Rb	1.61
9 CN Rb	1.63
10 CN Rb	1.66
11 CN Rb	1.69
12 CN Rb	1.72
14 CN Rb	1.83
6 CN Cs	1.67
8 CN Cs	1.74
9 CN Cs	1.78
10 CN Cs	1.81
11 CN Cs	1.85
12 CN Cs	1.88

Table S3. Crystallographic data and list of ionic radii obtained from Shannon[1] for calculation of effective ionic radii (eIR) values and average d_{U-M} distances.

Complex	Counterion Identity	Coordination Number to Oxygen	d_{U-M} (Å)	average d_{U-M} (Å)	Effective Ionic Radius (Å)
6	Li1	4	3.352	5.766	0.590
	Li2	4	5.966		
	Li3	4	5.3618		
	Li4	4	5.867		
	Li5	4	7.053		
	Li6	4	5.076		
	Li7	4	6.571		
	Li8	4	6.879		
7	K1	8	7.6284	7.190	1.473
			6.3598		
	K2	10	8.9694		
			4.231		
	K3	7	6.7831		
			5.7107		
	K4	7	7.2638		
			6.7691		
	K5	6	9.895		
			7.3104		
	K6	8	6.8617		
			7.08		
	K7	9	9.4721		
			6.8954		
	K8A	5	4.1711		
			10.2158		
	K8B	4	4.1544		
			10.0429		
	K9A	8	4.1104		
			9.6393		
	K9B	7	4.0113		
			9.7589		
	K10A	6	5.7		
			9.1234		
	K10B	5	6.117		
			8.1021		
	K10C	6	6.7781		
			7.7322		
8	Cs1	7	5.1637	5.941	1.736
	Cs2	6	4.5443		
	Cs3	9	7.4749		
	Cs4	8	4.7872		
	Cs5	8	7.581		
	Cs6	8	6.8909		
	Cs7	7	6.6924		
	Cs8	8	4.3964		

Counterion with CN	Ionic Radius (Å)
4 CN Li	0.59
6 CN Li	0.76
8 CN Li	0.92
4 CN Na	0.99
5 CN Na	1
6 CN Na	1.02
7 CN Na	1.12
8 CN Na	1.18
9 CN Na	1.24
10 CN Na	1.39
4 CN K	1.37
6 CN K	1.38
7 CN K	1.46
8 CN K	1.51
9 CN K	1.55
10 CN K	1.59
12 CN K	1.64
6 CN Rb	1.52
7 CN Rb	1.56
8 CN Rb	1.61
9 CN Rb	1.63
10 CN Rb	1.66
11 CN Rb	1.69
12 CN Rb	1.72
14 CN Rb	1.83
6 CN Cs	1.67
8 CN Cs	1.74
9 CN Cs	1.78
10 CN Cs	1.81
11 CN Cs	1.85
12 CN Cs	1.88

Partial Least Squares (PLS) Analysis. PLS is a mix of both principal component analysis and multiple regressions and is often used to identify factors that contribute to the covariances between independent and dependent variables.[2] It does this by decomposing the independent variables into latent variables with weighting scores that best correlate with the dependent variables which differentiate it from principle component analysis that use weighting scores to capture the variance in independent variables. Partial Least Square analysis was conducted with the OriginPro2024 software package[3] using the singular value decomposition (SVD) method in which:

$$X = n \bullet m$$
$$Y = n \bullet r$$

Where X is the matrix size for the independent variables and Y is the matrix size for the dependent variables and n, m, and r are the number of observations, number of independent variables, and number of dependent variables, respectively. The mean from each column in matrix X and Y are subtracted to produce X_0 and Y_0. In SVD the weight (w) of the independent variables is extracted by normalizing the first left singular vector of $X_0^T Y_0$ which then is used to calculate:

$$t = X_0 w$$
$$p = X_0^T t$$
$$q = Y_0^T t$$
$$u = Y_0 q$$

where t, p, q, and u are the x scores, y scores, x loadings, and y loadings which can also be expressed by matrices T, P, Q, and U. These parameters are then refined against the residual matrices, **k**, until they converge, and **k** factors can be synthesized to construct the model.

The leave-one-out cross-validation (CV) method tests the performance of a model and is often used to prevent overfitting of a model. It does this by iteratively generating models with n-number of latent variables (v = 1, 2, 3, …, m) and leaving one of the datasets as a benchmark to test the model produced by the latent variables. The parameter often used to measure the quality of CV method model is the minimum root mean PRESS (predicted residual sum of squares) which is calculated using the following equation in Origin2024:

$$PRESS = \sum_{i=1}^{n} \sum_{j=1}^{r} \left(Y_{ij} - \hat{Y}_{ij}\right)^2$$

$$Root\ Mean\ PRESS = \sqrt{\frac{PRESS}{(n-1)r}}$$

Y_{ij} represents the predicted value of Y based on the leave-one-out cross-validation test. Prediction of dependent variables can then be done by calculating the coefficients of the fitted model which can be obtained from the following equation:

$$C = W(P^T W)^{-1} Q^T$$

where C and W are coefficient and weight matrices, respectively. The predicted dependent variables value can then be calculated by:

$$\hat{Y}_0 = C X_0$$

From PLS analysis, a variety of figures are generated; however, only the root mean PRESS plots, variable influence on projection (VIP) plots, X & Y loading plots, Y-variance accountability plots, and diagnostic plots are presented here. The root mean PRESS plot shows the results of the CV test with the optimal number of latent variables having the lowest root mean PRESS value. The VIP plots show the significance of each independent variable related to the dependent variables with a VIP value above 0.8 indicating statistical significance. The loading plots offer a visual look on how each of the dependent and independent variables relate with the extracted latent variables, and these are powerful plots which show true correlations between the dependent and independent variables. Variables with vectors pointing to similar areas in the plot have proportional correlation while vectors pointing to opposite direction indicate inversely proportional correlation. The Y-variance accountability plots show how each factor accounts for the variance in the dependent (Y) variable. For model that doesn't pass the CV test, the Y-variance accountability plot is invalid due to overfitting of the model. In Origin2024 the variance explained for Y-variables is calculated by:

$$\frac{\sum_{j=1}^{k} Q_{lj}^{2}}{\sum_{i=1}^{n} {Y_0}_{il}^{2}}$$

Four diagnostic plots are generated by the PLS analysis which include: the actual versus predicted dependent variable value plots which show the quality of the model generated based on the independent variables inputted, the residual versus observed and predicted dependent variable plots that provide information regarding the distribution of the dependent variable dataset where a normal distribution exhibits random scattering of the data around the fitted line with a constant bandwidth, and the normal percentile plot which also provides information about data distribution with a normal distribution of data represented by the residual against percentiles forming a linear line.

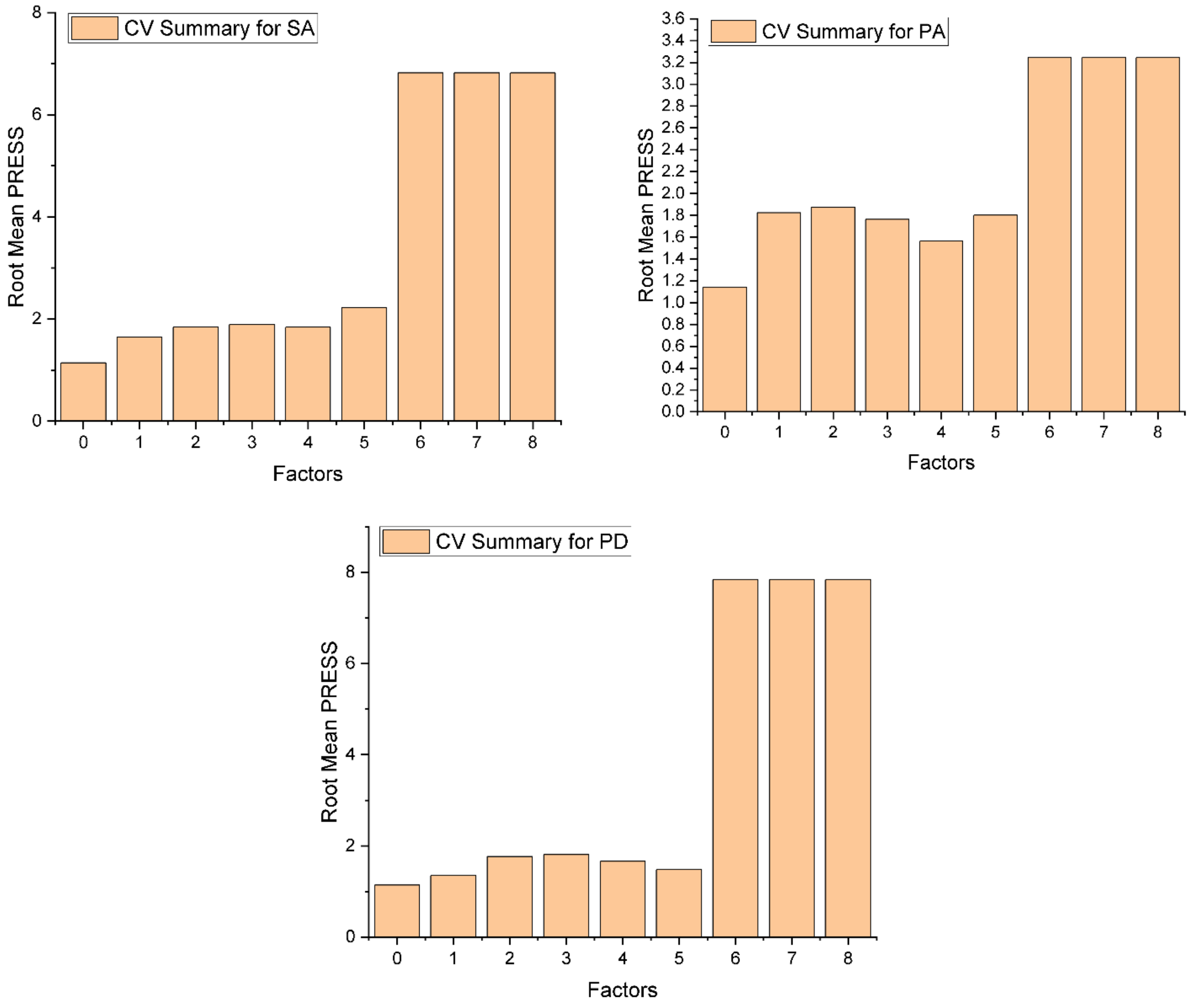

Figure S14. (**Top Left**) Root mean PRESS plot for skew angles, (**Top Right**) Root mean PRESS plot for plane angles, and (**Bottom**) Root mean PRESS plot for plane distances where structural parameters (a, b, c, β, V, average $d_{U\text{-}O}$ distance, average $d_{U\text{-}M}$ distance, and eIR) are independent variables and structure distortion parameters are dependent variables, which show the optimal number of latent variables needed to account for the variances in the distortion parameters in **1-8**.

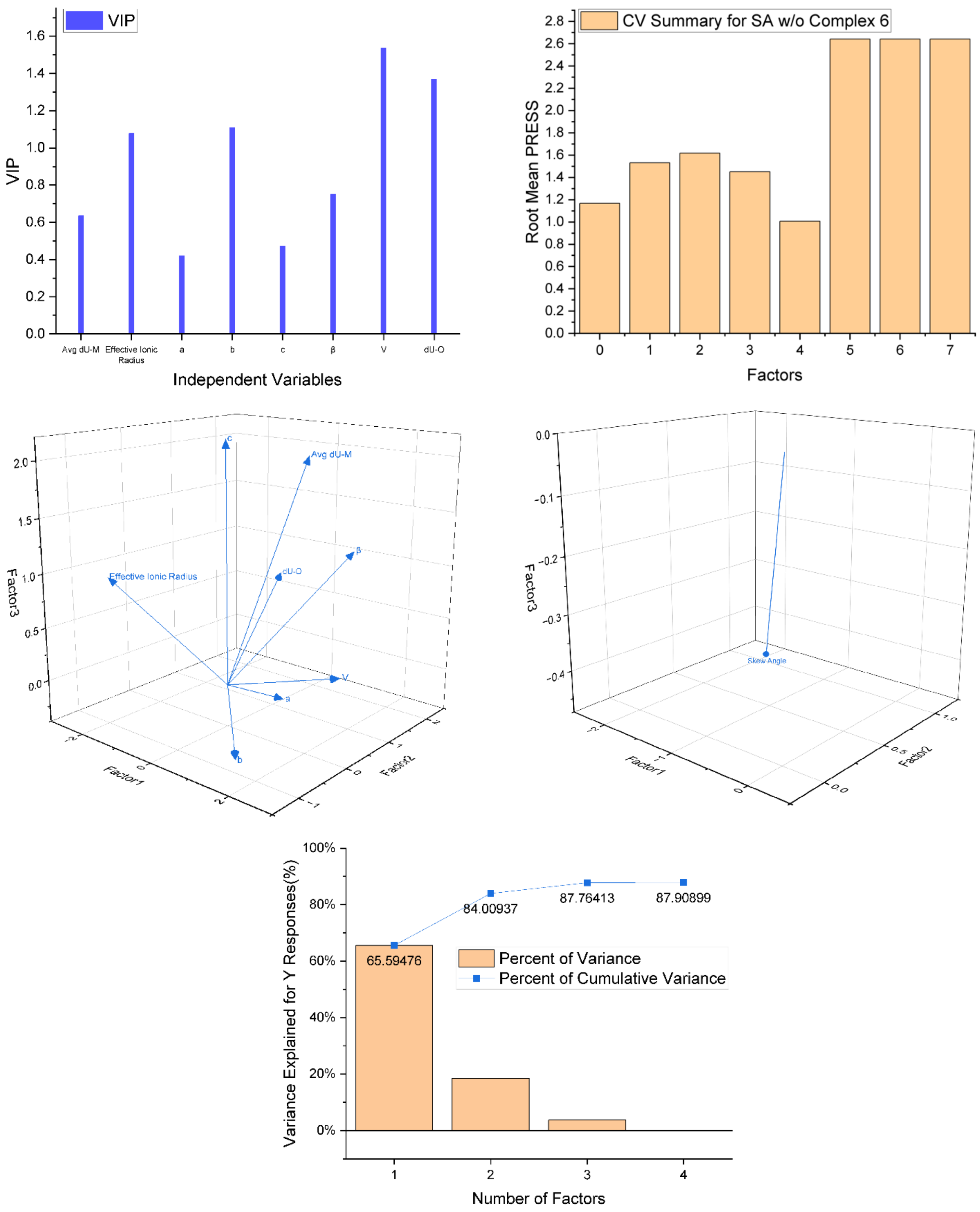

Figure S15. (**Top Left**) VIP plot, (**Top Right**) RMS plot, (**Middle Left**) X-loading plot, (**Middle Right**) Y-loading plot (mid right), and (**Bottom**) Y-variance accountability plot for PLS analysis with structural parameters (a, b, c, β, V, average d_{U-O} distance, average d_{U-M} distance, and eIR) as the independent variables and skew angle as the dependent variable.

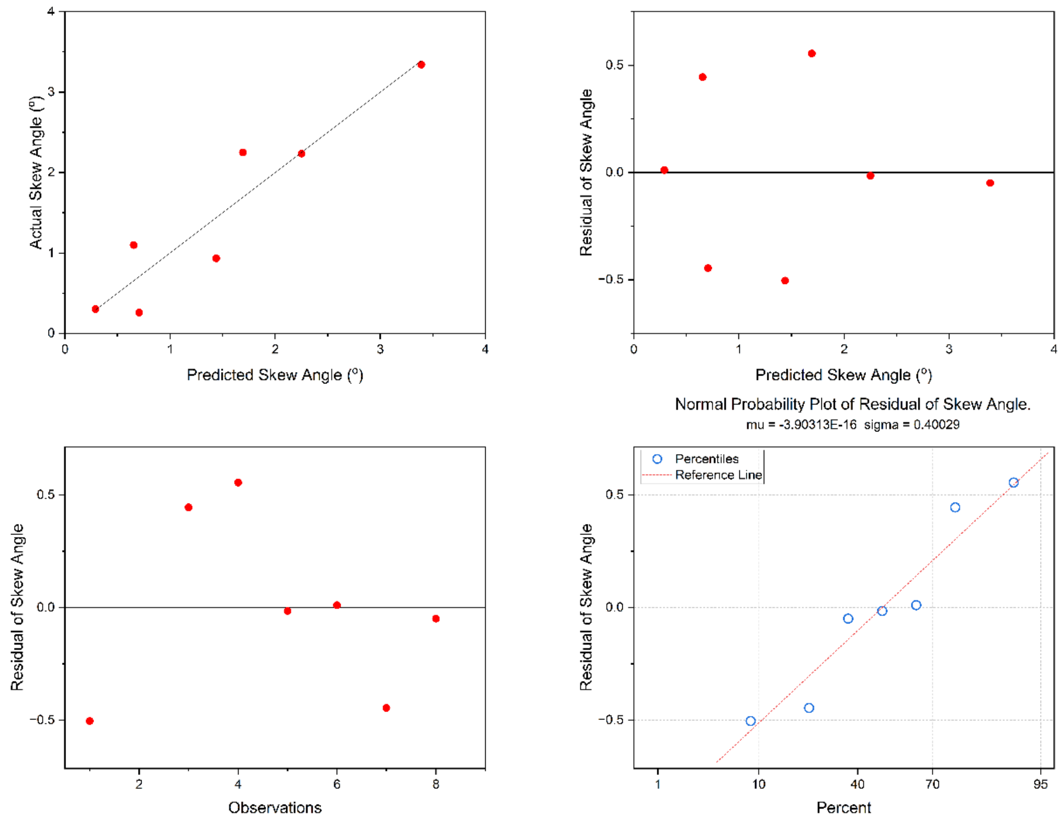

Figure S16. PLS analysis diagnostic plots for model with structural parameters (a, b, c, β, V, average $d_{U\text{-}O}$ distance, average $d_{U\text{-}M}$ distance, and eIR) as the independent variables and skew angle as the dependent variable.

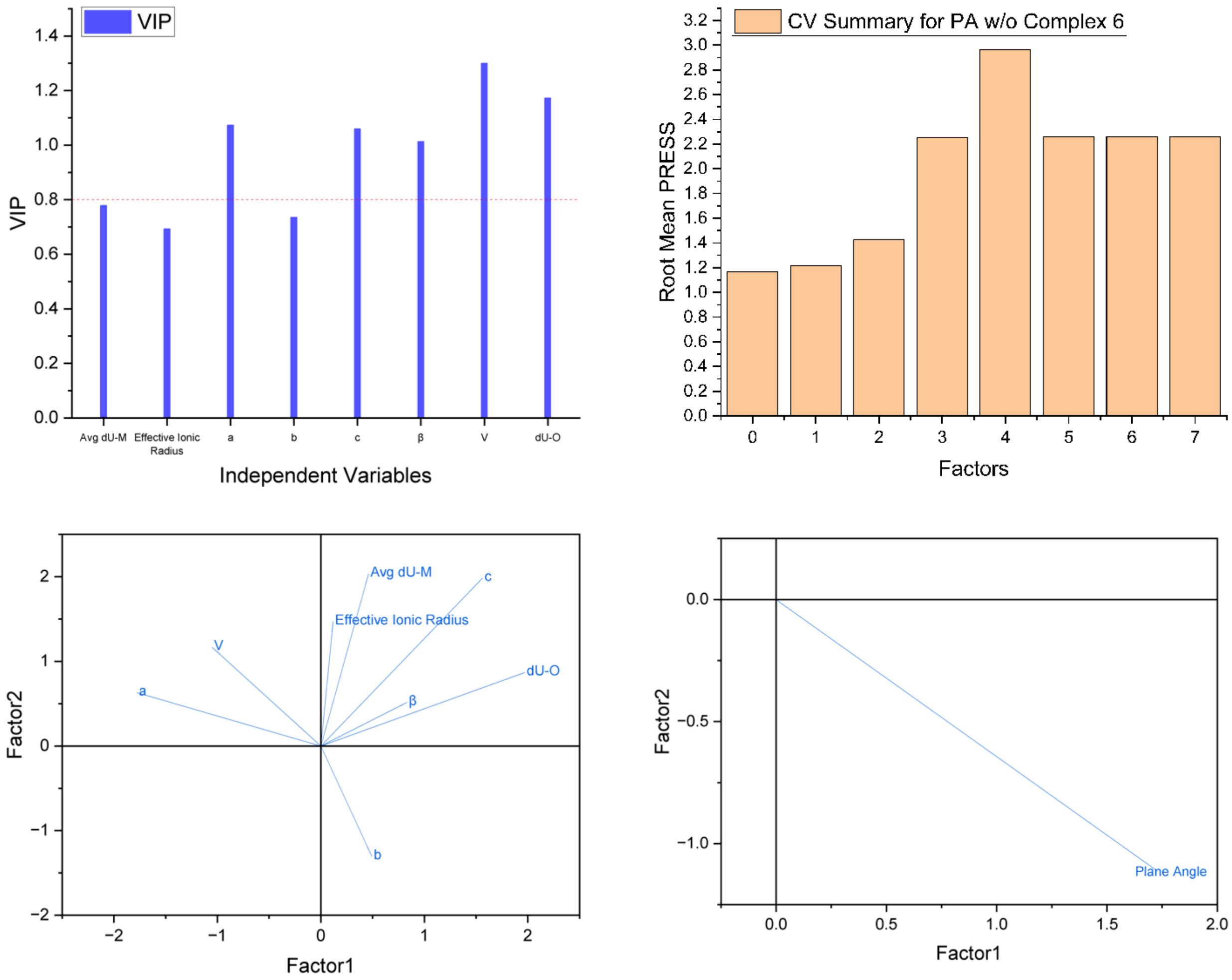

Figure S17. (**Top Left**) VIP plot, **(Top Right**) RMS plot, (**Bottom Left**) X-loading plot, and (**Bottom Right**) Y-loading plot for PLS analysis comparing structural parameters (a, b, c, β, V, average $d_{U\text{-}O}$ distance, average $d_{U\text{-}M}$ distance, and eIR) as the independent variables and plane angle as the dependent variable. The RMS plot shows that there are no latent variables can be built out of the independent variables. The VIP plot and X- and Y-loading plots were acquired by bypassing the cross-validation test. The loading plots show that averaged $d_{U\text{-}O}$ bond distances and a axis lengths to be most correlated with plane angles

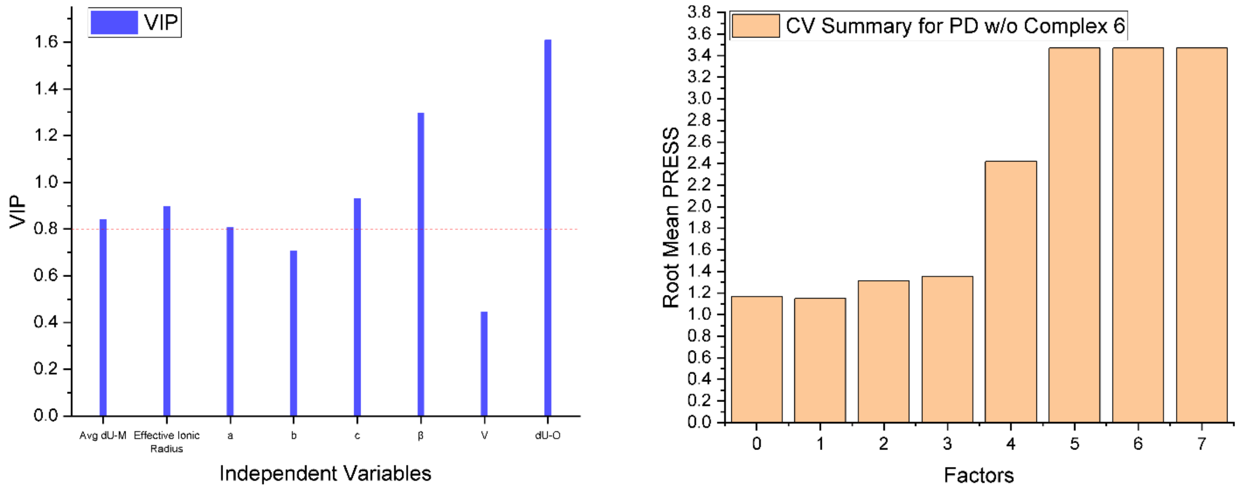

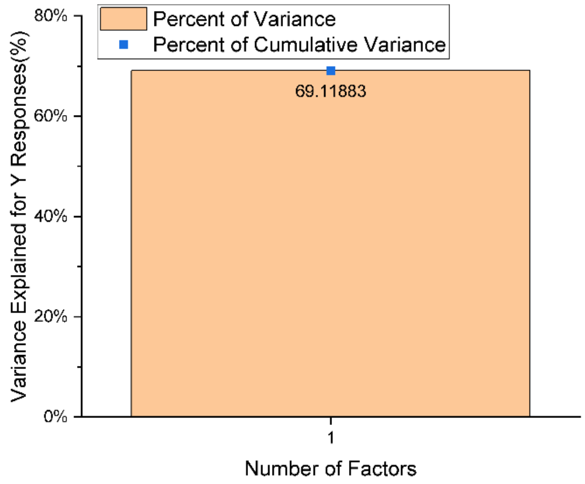

X Variable	Factor1	Y Variable	Factor1
Average $d_{U\text{-}M}$	1.72797	Plane Distance	-2.03645
Effective Ionic Radius	-1.06358		
a	-0.76667		
b	0.90123		
c	1.65317		
β	2.07104		
V	1.01698		
dU-O	1.8135		

Figure S18. (**Top Left**) VIP plot, (**Top Right**) RMS plot, (**Bottom Left**) Y-variance accountability plot, and (**Bottom Right**) loading table for PLS analysis with structural parameters (a, b, c, β, V, average $d_{U\text{-}O}$ distance, average $d_{U\text{-}M}$ distance, and eIR) as the independent variables and plane distance as the dependent variable.

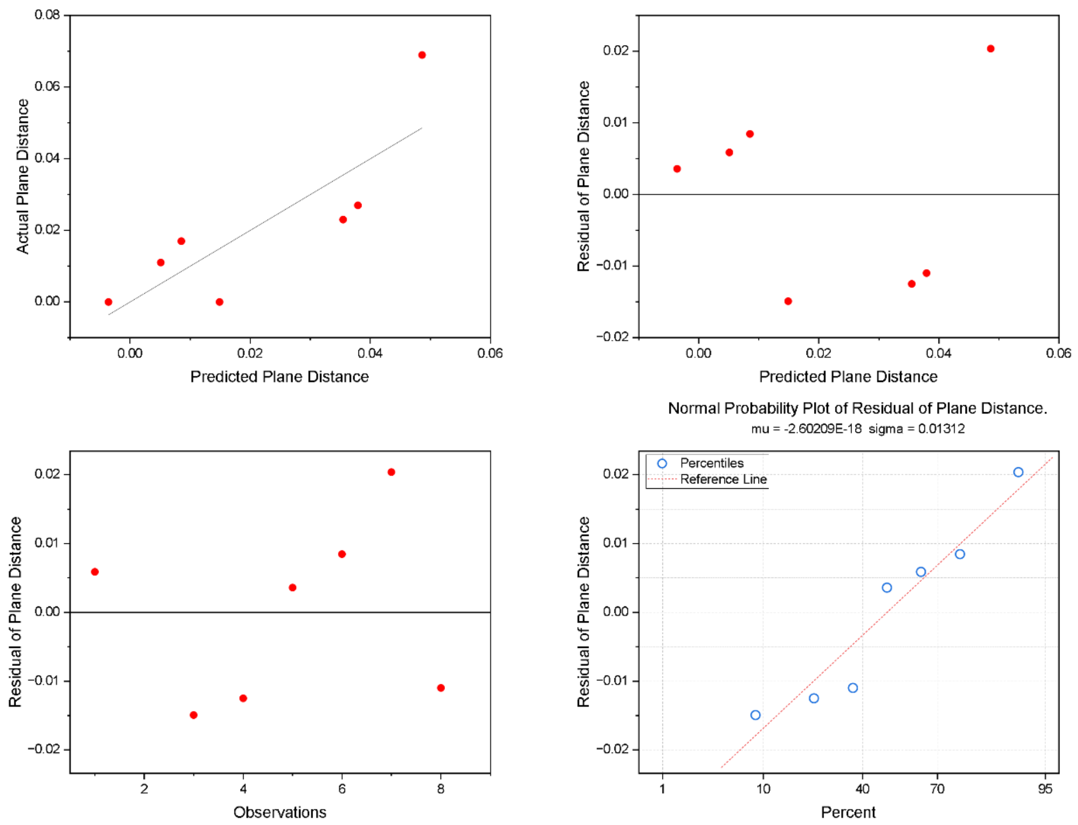

Figure S19. PLS analysis diagnostic plots for model with structural parameters (a, b, c, β, V, average $d_{U\text{-}O}$ distance, average $d_{U\text{-}M}$ distance, and eIR) as the independent variables and plane distance as the dependent variable.

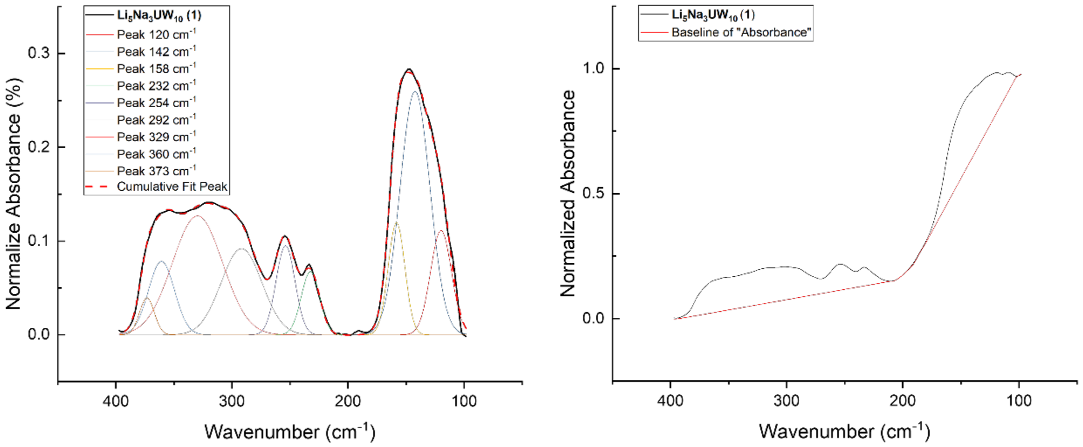

Figure S20. (**Left**) Baseline corrected FIR spectrum of complex **1** ($Li_5Na_3[UW_{10}]$) fit to Gaussian functions. (**Right**) Raw FIR spectrum for complex **1** and baseline used for baseline correction.

Table S4. Peak fitting parameters for FIR spectrum of complex **1**.

Model	Gauss								
Equation	y=y0 + (A/(w*sqrt(pi/2)))*exp(-2*((x-xc)/w)^2)								
Plot	Peak1	Peak2	Peak3	Peak4	Peak5	Peak6	Peak7	Peak8	Peak9
y0	-2.03096E-4 ± 8.60774E-4	-2.03096E-4 ± 8.60774E-4	-2.03096E-4 ± 8.60774E-4	-2.03096E-4 ± 8.60774E-4	-2.03096E-4 ± 8.60774E-4	-2.03096E-4 ± 8.60774E-4	-2.03096E-4 ± 8.60774E-4	-2.03096E-4 ± 8.60774E-4	-2.03096E-4 ± 8.60774E-4
xc	119.92835 ± 0.79841	142.3227 ± 1.23895	158.26848 ± 0.38488	232.4577 ± 0.52765	253.90189 ± 0.3486	291.80422 ± 5.5708	329.74718 ± 3.88353	360.76675 ± 4.84083	373.34227 ± 0.96665
w	18.06522 ± 0.84825	25.72409 ± 1.50184	14.4503 ± 1.02939	15.84854 ± 0.86227	16.31117 ± 0.84179	35.17672 ± 4.53679	43.26784 ± 18.9052	22.45852 ± 10.37438	12.82969 ± 4.48592
A	2.52128 ± 0.37995	8.37979 ± 0.67755	2.16605 ± 0.54641	1.33775 ± 0.09331	1.94591 ± 0.14436	4.05903 ± 2.39472	6.88578 ± 4.07859	2.2079 ± 2.70327	0.63184 ± 0.9542
Reduced Chi-Sqr	1.00973E-5								
R-Square (COD)	0.99859								
Adj. R-Square	0.99829								

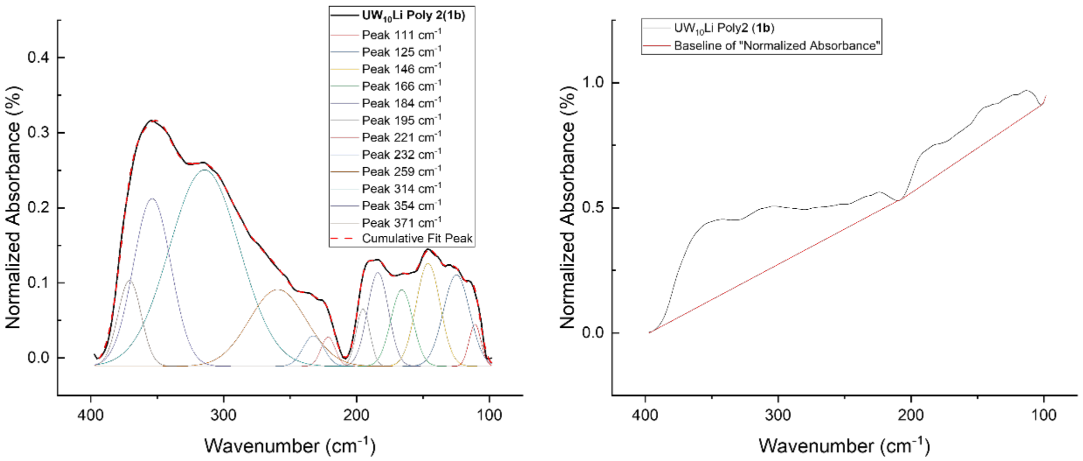

Figure S21. (**Left**) Baseline corrected FIR spectrum of complex **1b** ($UW_{10}Li$ polymorph) fit to Gaussian functions. (**Right**) Raw FIR spectrum for complex **1b** and baseline used for baseline correction.

Table S5. Peak fitting parameters for FIR spectrum of complex **1b**.

Model	Gauss											
Equation	y=y0 + (A/(w*sqrt(pi/2)))*exp(-2*((x-xc)/w)^2)											
Plot	Peak1	Peak2	Peak3	Peak4	Peak5	Peak6	Peak7	Peak8	Peak9	Peak10	Peak11	Peak12
y0	-0.01119 ± 0.0019	-0.01119 ± 0.0019	-0.01119 ± 0.0019	-0.01119 ± 0.0019	-0.01119 ± 0.0019	-0.01119 ± 0.0019	-0.01119 ± 0.0019	-0.01119 ± 0.0019	-0.01119 ± 0.0019	-0.01119 ± 0.0019	-0.01119 ± 0.0019	-0.01119 ± 0.0019
xc	110.78843 ± 0.28949	124.69768 ± 1.84578	146.05676 ± 1.97539	165.85352 ± 3.70955	183.84179 ± 1.76724	195.10791 ± 1.40377	221.35933 ± 1.40845	232.43321 ± 3.75016	259.25173 ± 4.55581	314.407 ± 1.33723	353.8329 ± 1.94894	370.81846 ± 0.63702
w	9.03203 ± 1.03248	19.84784 ± 3.1231	18.04785 ± 4.75132	16.57888 ± 10.32922	15.77877 ± 11.69537	10.26098 ± 2.58448	10.71649 ± 2.07289	15.63346 ± 4.74377	42.78328 ± 6.10318	52.6099 ± 9.08122	28.24858 ± 4.33432	17.44421 ± 1.73215
A	0.63371 ± 0.20115	3.03474 ± 0.74732	3.10502 ± 1.46278	2.12275 ± 2.56511	2.47178 ± 3.0271	0.98118 ± 1.36437	0.52407 ± 0.38533	0.79647 ± 0.47188	5.49613 ± 1.87277	17.26451 ± 3.69307	7.91691 ± 3.09248	2.50512 ± 1.23495
Reduced Chi-Sqr	7.20338E-6											
R-Square (COD)	0.99929											
Adj. R-Square	0.99908											

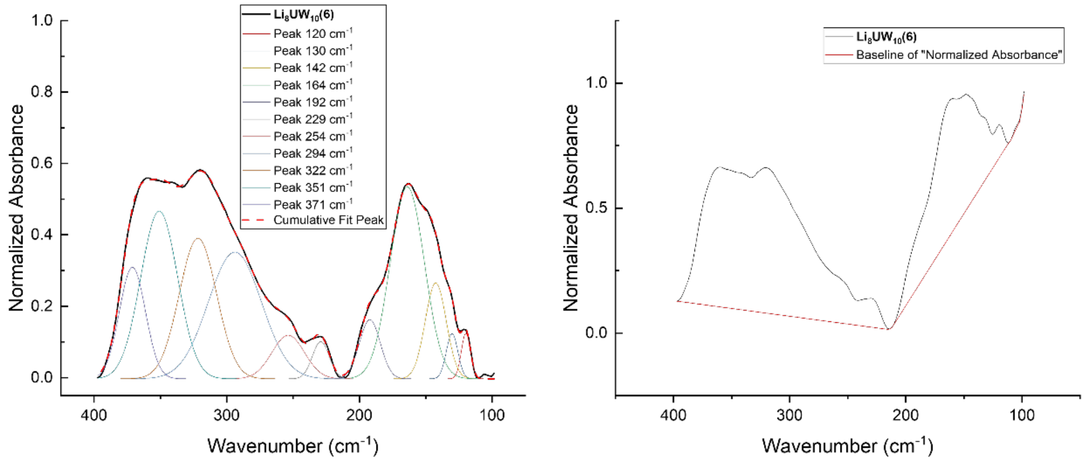

Figure S22. (**Left**) Baseline corrected FIR spectrum of complex **6** ($Li_8[UW_{10}]$) fit to Gaussian functions. (**Right**) Raw FIR spectrum for complex **6** and baseline used for baseline correction.

Table S6. Peak fitting parameters for FIR spectrum of complex **6**.

Model	Gauss								
Equation	y=y0 + (A/(w*sqrt(pi/2)))*exp(-2*((x-xc)/w)^2)								
Plot	Peak1	Peak2	Peak3	Peak4	Peak5	Peak6	Peak7	Peak8	Peak9
y0	-0.00298	-0.00298	-0.00298	-0.00298	-0.00298	-0.00298	-0.00298	-0.00298	-0.00298
	± 0.00172	± 0.00172	± 0.00172	± 0.00172	± 0.00172	± 0.00172	± 0.00172	± 0.00172	± 0.00172
xc	119.70141	130.10179	142.42993	163.88458	192.09657	229.09064	253.64715	293.85201	321.70241
	± 0.30947	± 0.38582	± 0.49482	± 0.7637	± 0.512	± 0.39062	± 3.82717	± 22.51419	± 3.94814
w	6.9503	8.73108	15.83783	25.87148	15.75573	12.71102	24.59651	40.04385	28.12417
	± 0.46131	± 1.3194	± 2.08814	± 1.8263	± 0.68429	± 0.70175	± 5.80945	± 33.76097	± 16.1154
A	1.10681	1.38034	5.32392	17.42803	3.27114	1.66165	3.7571	17.81935	13.86751
	± 0.1063	± 0.50463	± 1.53644	± 1.46504	± 0.35703	± 0.18684	± 3.36756	± 25.38381	± 30.97531
Plot	Peak10	Peak11							
y0	-0.00298	-0.00298							
	± 0.00172	± 0.00172							
xc	350.90676	371.09358							
	± 2.31172	± 2.11217							
w	27.85996	19.63799							
	± 16.27449	± 2.68318							
A	16.40187	7.68469							
	± 16.79129	± 7.19946							
Reduced Chi-Sqr	2.66E-05								
R-Square (COD)	0.99947								
Adj. R-Square	0.99932								

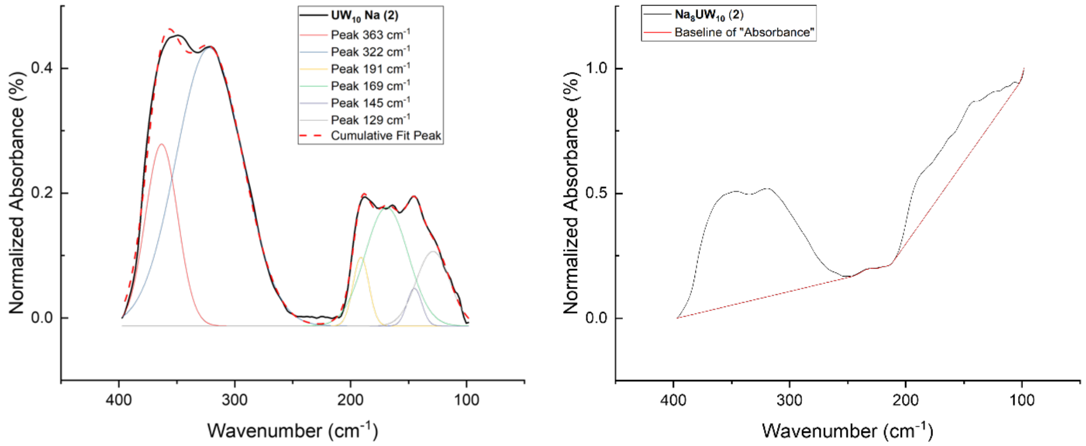

Figure S23. Baseline corrected FIR spectrum of complex **2** ($Na_8[UW_{10}]$) fit to Gaussian functions. (**Right**) Raw FIR spectrum for complex **2** and baseline used for baseline correction.

Table S7. Peak fitting parameters for FIR spectrum of complex **2**

Model	Gauss					
Equation	y=y0 + (A/(w*sqrt(pi/2)))*exp(-2*((x-xc)/w)^2)					
Plot	Peak1	Peak2	Peak3	Peak4	Peak5	Peak6
y0	--0.01291 ± 0.0028	--0.01291 ± 0.0028	--0.01291 ± 0.0028	--0.01291 ± 0.0028	--0.01291 ± 0.0028	--0.01291 ± 0.0028
xc	128.99301 ± 3.80297	145.01953 ± 1.02274	168.99171 ± 2.2171	191.14131 ± 0.46737	321.97876 ± 0.76352	363.20816 ± 0.30178
w	28.91956 ± 4.44724	12.78379 ± 3.21521	37.16238 ± 2.92343	13.73074 ± 1.33606	57.45007 ± 1.30763	27.81764 ± 0.75932
A	4.33285 ± 1.136	0.96599 ± 0.63274	8.85514 ± 0.86658	1.89383 ± 0.36842	32.09084 ± 0.96952	10.15948 ± 0.67304
Reduced Chi-Sqr	8.18912E-5					
R-Square (COD)	0.99702					
Adj. R-Square	0.99663					

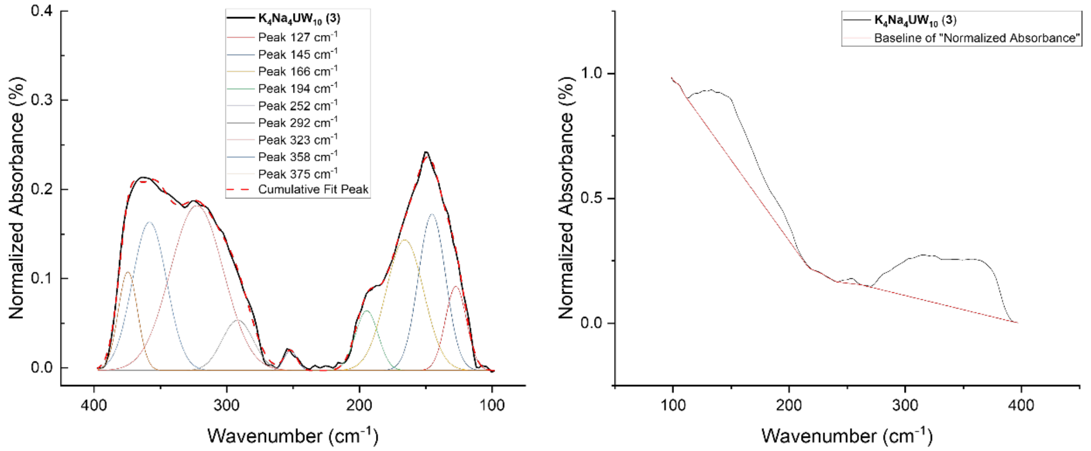

Figure S24. (**Left**) Baseline corrected FIR spectrum of complex **3** ($K_4Na_4[UW_{10}]$) fit to Gaussian functions. (**Right**) Raw FIR spectrum for complex **3** and baseline used for baseline correction.

Table S8. Peak fitting parameters for FIR spectrum of complex **3**.

Model	Gauss								
Equation	y=y0 + (A/(w*sqrt(pi/2)))*exp(-2*((x-xc)/w)^2)								
Plot	Peak1	Peak2	Peak3	Peak4	Peak5	Peak6	Peak7	Peak8	Peak9
y0	-0.00305 ± 0.00101	-0.00305 ± 0.00101	-0.00305 ± 0.00101	-0.00305 ± 0.00101	-0.00305 ± 0.00101	-0.00305 ± 0.00101	-0.00305 ± 0.00101	-0.00305 ± 0.00101	-0.00305 ± 0.00101
xc	127.24406 ± 2.61697	145.23577 ± 4.23626	165.74302 ± 19.15949	194.40947 ± 2.46186	251.95641 ± 0.68395	291.72739 ± 3.23013	322.50043 ± 2.02126	358.2202 ± 2.11125	374.51647 ± 0.40222
w	15.82953 ± 1.89683	20.51088 ± 10.08883	28.45093 ± 28.29195	17.06999 ± 2.92303	9.70011 ± 1.52972	23.50343 ± 7.61903	40.06467 ± 16.41011	25.81859 ± 3.97985	14.57698 ± 1.43
A	1.87186 ± 1.12684	4.50718 ± 8.65185	5.23017 ± 8.73718	1.43515 ± 1.07334	0.27251 ± 0.05033	1.66672 ± 2.393	9.28883 ± 4.39961	5.3885 ± 2.38258	2.02218 ± 0.69979
Reduced Chi-Sqr	1.83067E-5								
R-Square (COD)	0.99776								
Adj. R-Square	0.99729								

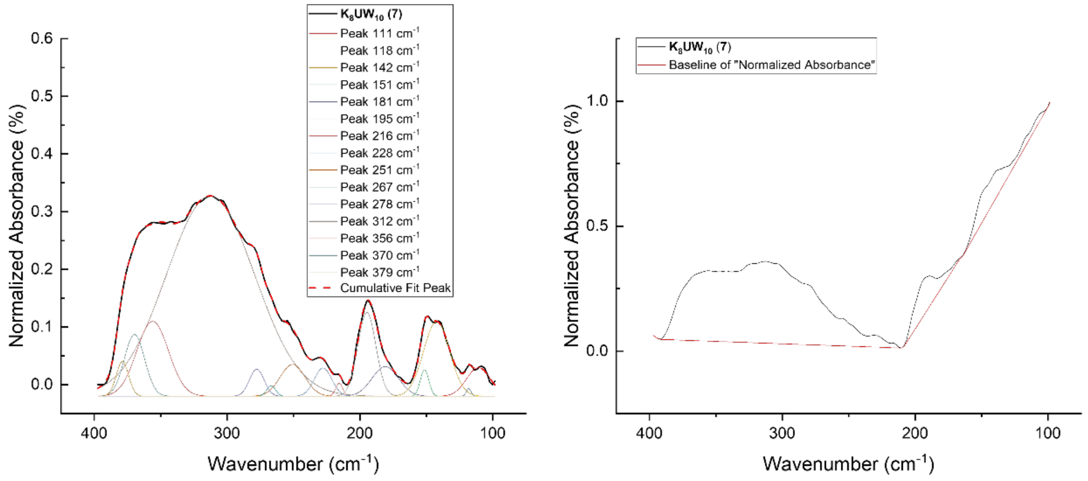

Figure S25. (**Left**) Baseline corrected FIR spectrum of complex **7** ($K_8[UW_{10}]$) fit to Gaussian functions. (**Right**) Raw FIR spectrum for complex **7** and baseline used for baseline correction.

Table S9. Peak fitting parameters for FIR spectrum of complex **7**.

Model	Gauss								
Equation	y=y0 + (A/(w*sqrt(pi/2)))*exp(-2*((x-xc)/w)^2)								
Plot	Peak1	Peak2	Peak3	Peak4	Peak5	Peak6	Peak7	Peak8	Peak9
y0	-0.01995 ± 0.00451	-0.01995 ± 0.00451	-0.01995 ± 0.00451	-0.01995 ± 0.00451	-0.01995 ± 0.00451	-0.01995 ± 0.00451	-0.01995 ± 0.00451	-0.01995 ± 0.00451	-0.01995 ± 0.00451
xc	110.66547 ± 0.64589	118.09153 ± 0.44816	142.21352 ± 0.45541	151.25425 ± 0.2084	180.8543 ± 5.16323	194.7441 ± 0.56233	215.55732 ± 0.41228	227.72417 ± 0.49821	250.62901 ± 0.59929
w	18.05097 ± 1.41375	3.57942 ± 1.14167	19.67108 ± 0.91048	6.41765 ± 0.69148	19.97274 ± 6.35364	13.59719 ± 0.82287	5.3526 ± 0.97479	13.17837 ± 1.54486	18.71052 ± 2.31828
A	1.12157 ± 0.16527	0.05931 ± 0.02518	3.18709 ± 0.23777	0.36926 ± 0.08046	1.28859 ± 0.691	2.47531 ± 0.65827	0.15147 ± 0.0453	0.80837 ± 0.11905	1.29513 ± 0.21705
Plot	Peak10	Peak11	Peak12	Peak13	Peak14	Peak15			
y0	-0.01995 ± 0.00451	-0.01995 ± 0.00451	-0.01995 ± 0.00451	-0.01995 ± 0.00451	-0.01995 ± 0.00451	-0.01995 ± 0.00451			
xc	266.80945 ± 0.87894	277.63162 ± 0.56064	312.47315 ± 0.57972	356.0299 ± 5.40819	369.70914 ± 2.45754	378.78567 ± 0.68558			
w	6.63862 ± 1.87734	11.06124 ± 1.34783	66.5155 ± 2.92366	23.82076 ± 5.39698	15.25297 ± 6.01216	9.33526 ± 2.27941			
A	0.15142 ± 0.08055	0.64885 ± 0.12987	28.95207 ± 1.61324	3.88502 ± 2.176	2.04593 ± 2.39586	0.7039 ± 0.79164			
Reduced Chi-Sqr	8.03E-06								
R-Square (COD)	0.99955								
Adj. R-Square	0.99937								

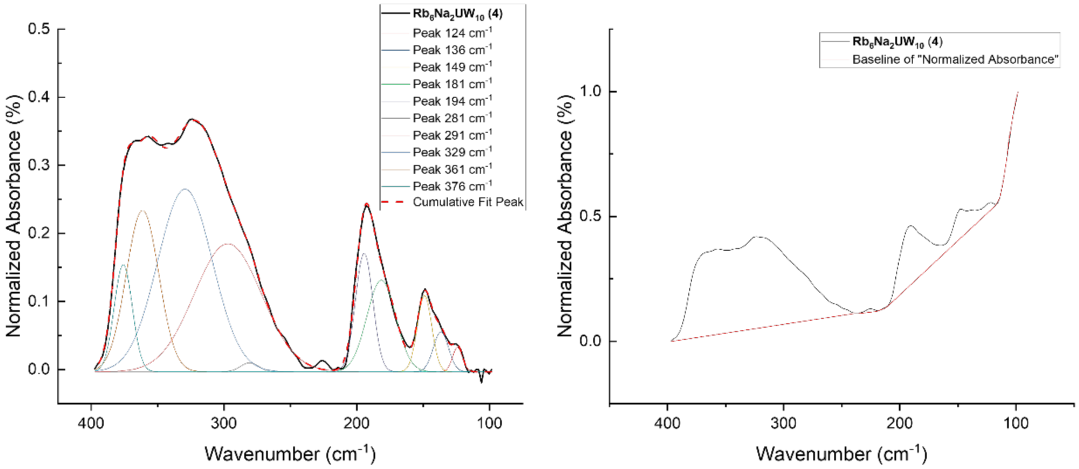

Figure S26. (**Left**) Baseline corrected FIR spectrum of complex **4** ($Rb_6Na_2[UW_{10}]$) fit to Gaussian functions. (**Right**) Raw FIR spectrum for complex **4** and baseline used for baseline correction.

Table S10. Peak fitting parameters for FIR spectrum of complex **4**.

	Gauss									
Equation	y=y0 + (A/(w*sqrt(pi/2)))*exp(-2*((x-xc)/w)^2)									
Plot	Peak1	Peak2	Peak3	Peak4	Peak5	Peak6	Peak7	Peak8	Peak9	Peak10
y0	-0.00619 ± 0.00237	-0.00619 ± 0.00237	-0.00619 ± 0.00237	-0.00619 ± 0.00237	-0.00619 ± 0.00237	-0.00619 ± 0.00237	-0.00619 ± 0.00237	-0.00619 ± 0.00237	-0.00619 ± 0.00237	-0.00619 ± 0.00237
xc	131.8297 ± 1.82835	144.34097 ± 1.6427	178.65823 ± 1.17784	195.29134 ± 0.63318	223.84427 ± 0.47997	250.90324 ± 1.4629	286.41821 ± 9.44483	325.75347 ± 3.07908	360.14822 ± 1.20258	375.9034 ± 0.36233
w	12.9807 ± 1.78884	12.44877 ± 1.62135	18.56329 ± 1.56475	13.70992 ± 0.72884	9.64545 ± 1.05759	11.06411 ± 4.48096	36.64753 ± 8.38508	39.44801 ± 12.05939	23.4657 ± 4.15365	13.3277 ± 1.10896
A	2.39308 ± 0.73005	2.3695 ± 0.72084	4.46297 ± 0.54287	3.74176 ± 0.51808	0.68781 ± 0.0804	0.30181 ± 0.20838	9.07758 ± 6.15109	19.72949 ± 8.7773	10.08715 ± 4.20293	4.54625 ± 1.41203
Reduced Chi-Sqr	6.5793E-5									
R-Square (COD)	0.9978									
Adj. R-Square	0.99728									

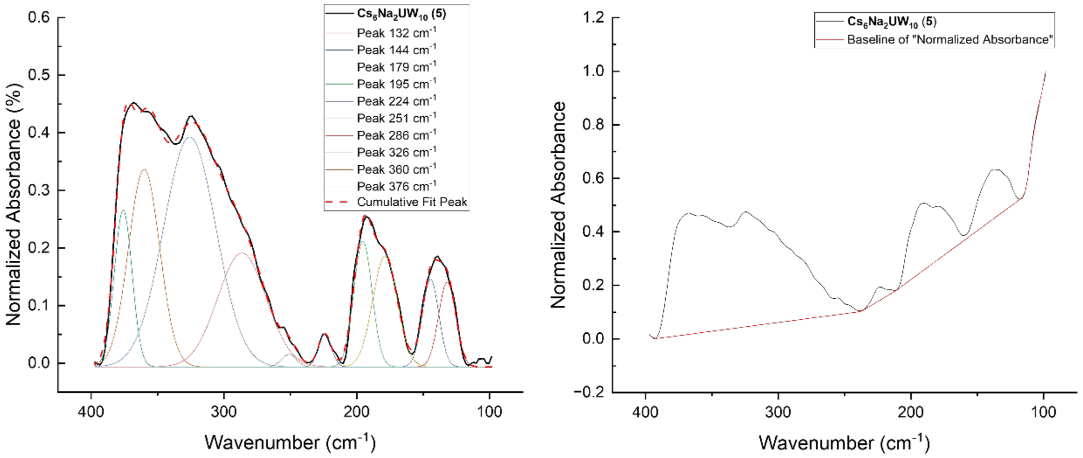

Figure S27. (**Left**) Baseline corrected FIR spectrum of complex **5** ($Cs_{5.5}Na_{2.5}[UW_{10}]$) fit to Gaussian functions. (**Right**) Raw FIR spectrum for complex **5** and baseline used for baseline correction.

Table S11. Peak fitting parameters for FIR spectrum of complex **5**.

Model	Gauss									
Equation	y=y0 + (A/(w*sqrt(pi/2)))*exp(-2*((x-xc)/w)^2)									
Plot	Peak1	Peak2	Peak3	Peak4	Peak5	Peak6	Peak7	Peak8	Peak9	Peak10
y0	-0.00318 ± 0.00131	-0.00318 ± 0.00131	-0.00318 ± 0.00131	-0.00318 ± 0.00131	-0.00318 ± 0.00131	-0.00318 ± 0.00131	-0.00318 ± 0.00131	-0.00318 ± 0.00131	-0.00318 ± 0.00131	-0.00318 ± 0.00131
xc	124.05252 ± 1.13706	136.5439 ± 2.03017	148.9018 ± 1.25966	181.44933 ± 2.69002	194.36752 ± 0.31921	280.68754 ± 2.02866	297.04884 ± 20.63355	329.30025 ± 4.97988	361.33413 ± 1.06671	375.8449 ± 0.23462
w	7.55042 ± 1.53639	11.42375 ± 5.09115	10.88301 ± 1.33028	21.72929 ± 2.8032	13.10301 ± 1.0031	13.59905 ± 6.12166	50.70308 ± 11.33969	41.17064 ± 13.96951	24.49718 ± 3.64016	13.65961 ± 1.06812
A	0.33821 ± 0.14363	0.83751 ± 0.50687	1.52361 ± 0.41021	3.66456 ± 0.9046	2.856 ± 0.83973	0.22483 ± 0.22394	11.95017 ± 12.23797	13.84247 ± 14.47513	7.27762 ± 3.21438	2.6912 ± 0.78649
Reduced Chi-Sqr	1.96515E-5									
R-Square (COD)	0.99908									
Adj. R-Square	0.99887									

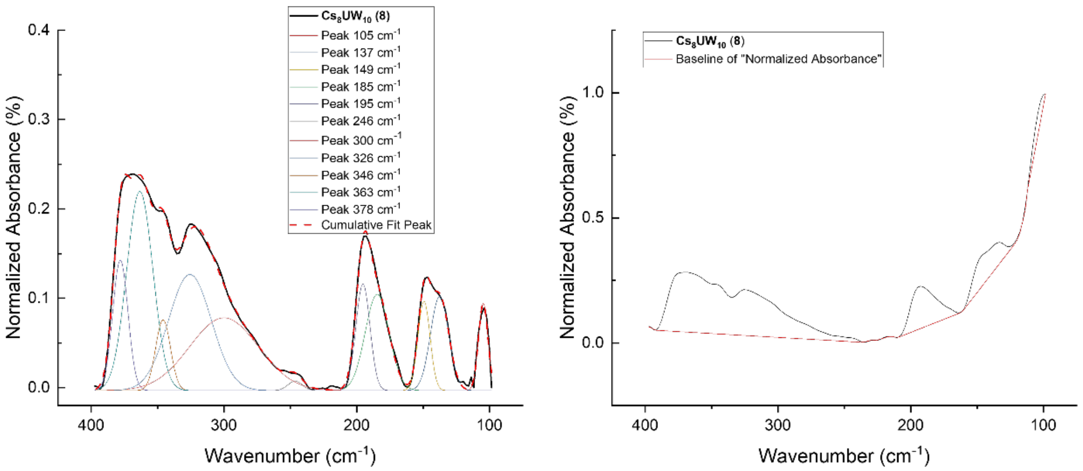

Figure S28. (**Left**) Baseline corrected FIR spectrum of complex **8** ($Cs_8[UW_{10}]$) fit to Gaussian functions. (**Right**) Raw FIR spectrum for complex **8** and baseline used for baseline correction.

Table S12. Peak fitting parameters for FIR spectrum of complex **8**.

Model	Gauss								
Equation	y=y0 + (A/(w*sqrt(pi/2)))*exp(-2*((x-xc)/w)^2)								
Plot	Peak1	Peak2	Peak3	Peak4	Peak5	Peak6	Peak7	Peak8	Peak9
y0	-0.0033 ± 0.00157	-0.0033 ± 0.00157	-0.0033 ± 0.00157	-0.0033 ± 0.00157	-0.0033 ± 0.00157	-0.0033 ± 0.00157	-0.0033 ± 0.00157	-0.0033 ± 0.00157	-0.0033 ± 0.00157
xc	104.67896 ± 0.11586	137.49743 ± 0.75122	149.29505 ± 0.41099	184.5631 ± 2.16243	195.28574 ± 0.3189	246.3884 ± 1.38989	299.94894 ± 24.70832	325.62653 ± 0.75879	345.86399 ± 0.7927
w	7.11248 ± 0.27543	13.75421 ± 1.05738	9.12973 ± 0.54847	18.02153 ± 2.20671	10.59092 ± 0.98215	10.87542 ± 4.00429	51.69604 ± 21.36156	32.36106 ± 6.88583	11.18321 ± 1.50227
A	0.86923 ± 0.03728	1.81667 ± 0.1945	1.14339 ± 0.17122	2.4338 ± 0.58078	1.58244 ± 0.52597	0.14381 ± 0.07963	5.27305 ± 5.32519	5.27159 ± 4.9722	1.11066 ± 0.53574
Plot	Peak10	Peak11							
y0	-0.0033 ± 0.00157	-0.0033 ± 0.00157							
xc	363.37718 ± 1.19077	378.03966 ± 0.52437							
w	19.79287 ± 4.69616	11.44985 ± 1.32187							
A	5.52969 ± 1.51045	2.09472 ± 0.98986							
Reduced Chi-Sqr	1.62E-05								
R-Square (COD)	0.9978								
Adj. R-Square	0.99721								

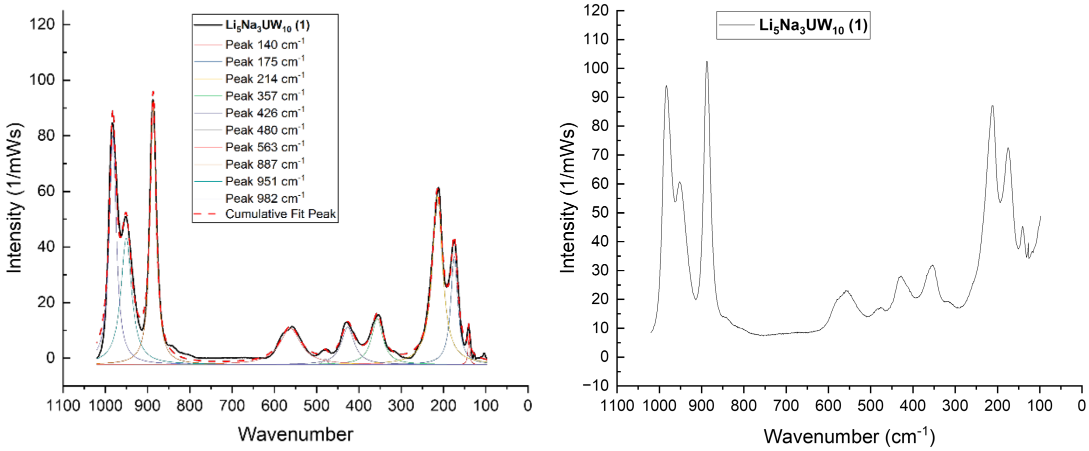

Figure S29. (**Left**) Baseline corrected Raman spectrum of complex **1** ($Li_5Na_3[UW_{10}]$) fit to Lorentzian functions. (**Right**) Raw Raman spectrum for complex **1**.

Table S13. Peak fitting parameters for Raman spectrum of complex **1**.

Equation	y = y0 + (2*A/pi)*(w/(4*(x-xc)^2 + w^2))								
Plot	Peak1	Peak2	Peak3	Peak4	Peak5	Peak6	Peak7	Peak8	Peak9
y0	-2.31178<br>± 0.14399	-2.31178<br>± 0.14399	-2.31178<br>± 0.14399	-2.31178<br>± 0.14399	-2.31178<br>± 0.14399	-2.31178<br>± 0.14399	-2.31178<br>± 0.14399	-2.31178<br>± 0.14399	-2.31178<br>± 0.14399
xc	140.22827<br>± 0.41193	174.52233<br>± 0.20212	214.35074<br>± 0.16296	356.81239<br>± 0.58015	426.40384<br>± 0.75887	480.03176<br>± 2.69647	562.52731<br>± 0.9579	886.96894<br>± 0.06773	950.55313<br>± 0.24397
w	5.07974<br>± 1.21997	20.06099<br>± 0.6961	29.44949<br>± 0.53876	32.76802<br>± 1.89692	36.91194<br>± 2.6521	11.68267<br>± 8.41097	59.91034<br>± 3.30067	16.98331<br>± 0.21191	30.80459<br>± 0.82846
A	72.23902<br>± 12.81707	1223.0461<br>± 35.75472	2797.91921<br>± 43.20626	850.38384<br>± 40.09915	784.36866<br>± 44.95211	37.58394<br>± 20.9502	1232.80736<br>± 57.4286	2526.63303<br>± 24.56035	2201.54648<br>± 55.44759
Plot	Peak10								
y0	-2.31178<br>± 0.14399								
xc	982.33354<br>± 0.09627								
w	19.82574<br>± 0.33432								
A	2563.94491<br>± 42.8396								
Reduced Chi-Sqr	3.8268								
R-Square (COD)	0.98991								
Adj. R-Square	0.98956								

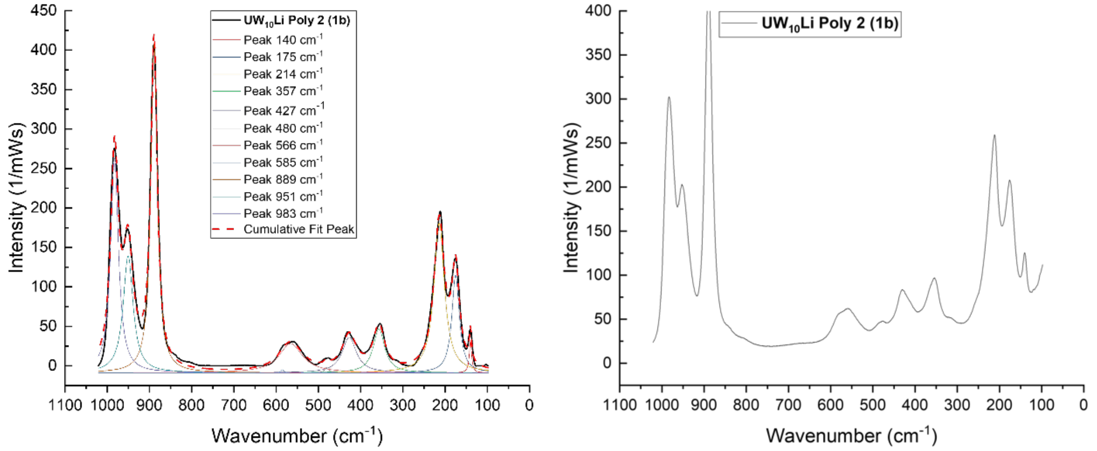

Figure S30. (**Left**) Baseline corrected Raman spectrum of complex **1b** ($UW_{10}Li$ polymorph) fit to Lorentzian functions. (**Right**) Raw Raman spectrum for complex **1b**.

Table S14. Peak fitting parameters for Raman spectrum of complex **1b**.

Model	Lorentz								
Equation	y = y0 + (2*A/pi)*(w/(4*(x-xc)^2 + w^2))								
Plot	Peak1	Peak2	Peak3	Peak4	Peak5	Peak6	Peak7	Peak8	Peak9
y0	-8.83522 ± 0.51269	-8.83522 ± 0.51269	-8.83522 ± 0.51269	-8.83522 ± 0.51269	-8.83522 ± 0.51269	-8.83522 ± 0.51269	-8.83522 ± 0.51269	-8.83522 ± 0.51269	-8.83522 ± 0.51269
xc	140.06054 ± 0.35574	175.2489 ± 0.22328	214.03769 ± 0.17955	356.71537 ± 0.57021	427.05887 ± 0.8036	480.32964 ± 2.75152	565.57879 ± 1.78341	584.90606 ± 3.78216	889.20053 ± 0.05348
w	6.73077 ± 1.07528	21.47789 ± 0.79206	29.92771 ± 0.59506	32.56043 ± 1.88154	40.4035 ± 2.89483	12.0403 ± 8.73241	67.34601 ± 4.3169	7.83241 ± 14.72243	18.50267 ± 0.16904
A	424.81681 ± 51.12175	4171.85004 ± 133.43359	8911.06597 ± 154.67425	2859.12033 ± 136.62882	2856.63411 ± 165.32719	128.37843 ± 73.47942	3923.18983 ± 256.82034	49.12591 ± 90.29668	12115.3211 ± 87.06682
Plot	Peak10	Peak11							
y0	-8.83522 ± 0.51269	-8.83522 ± 0.51269							
xc	950.57023 ± 0.23283	982.71803 ± 0.10583							
w	28.38541 ± 0.80096	22.27538 ± 0.36075							
A	6614.32497 ± 176.34942	9500.97774 ± 151.0752							
Reduced Chi-Sqr	42.29069								
R-Square (COD)	0.99209								
Adj. R-Square	0.99179								

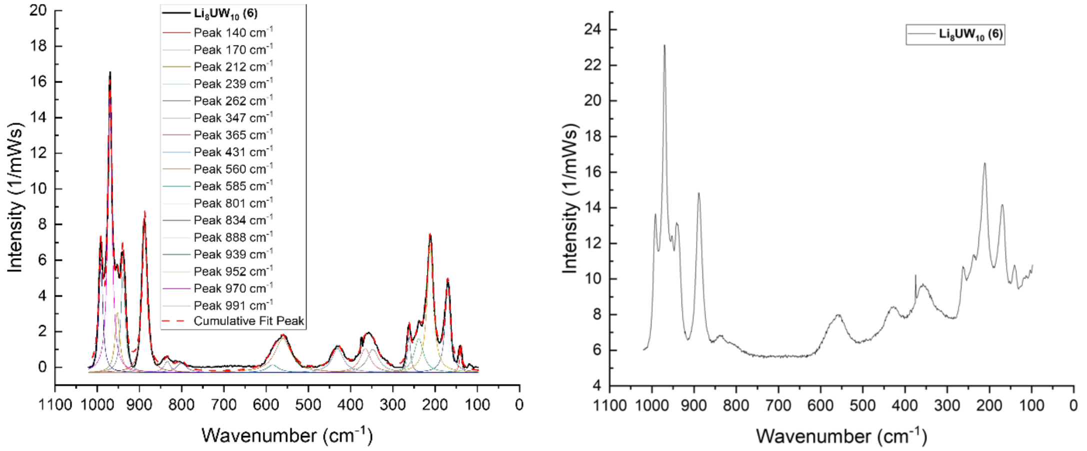

Figure S31. (**Left**) Baseline corrected Raman spectrum of complex **6** ($Li_8[UW_{10}]$) fit to Lorentzian functions. (**Right**) Raw Raman spectrum for complex **6**.

Table S15. Peak fitting parameters for Raman spectrum of complex **6**.

Model	Lorentz								
Equation	y = y0 + (2*A/pi)*(w/(4*(x-xc)^2 + w^2))								
Plot	Peak1	Peak2	Peak3	Peak4	Peak5	Peak6	Peak7	Peak8	Peak9
y0	-0.29412 ± 0.02184	-0.29412 ± 0.02184	-0.29412 ± 0.02184	-0.29412 ± 0.02184	-0.29412 ± 0.02184	-0.29412 ± 0.02184	-0.29412 ± 0.02184	-0.29412 ± 0.02184	-0.29412 ± 0.02184
xc	140.10181 ± 0.40499	170.2297 ± 0.15964	211.7569 ± 0.14089	238.6211 ± 0.72679	261.9496 ± 0.30579	347.36707 ± 3.77662	365.47466 ± 2.9758	430.6875 ± 0.88024	560.49574 ± 1.73892
w	6.11775 ± 1.20922	14.68128 ± 0.51036	17.40938 ± 0.51763	26.05086 ± 3.39489	8.83041 ± 1.15466	33.38254 ± 6.21477	29.63426 ± 6.58173	32.85389 ± 2.94512	46.15996 ± 3.67347
A	11.78614 ± 1.73433	112.2257 ± 3.01367	196.6329 ± 6.24655	77.9124 ± 10.01982	28.09061 ± 3.62316	68.32078 ± 32.77218	63.33374 ± 32.3527	69.02599 ± 5.16901	138.78501 ± 16.15471
Plot	Peak10	Peak11	Peak12	Peak13	Peak14	Peak15	Peak16	Peak17	
y0	-0.29412 ± 0.02184	-0.29412 ± 0.02184	-0.29412 ± 0.02184	-0.29412 ± 0.02184	-0.29412 ± 0.02184	-0.29412 ± 0.02184	-0.29412 ± 0.02184	-0.29412 ± 0.02184	
xc	584.52457 ± 3.92298	800.9875 ± 2.17184	834.2518 ± 1.36285	887.97022 ± 0.0778	938.9546 ± 0.16494	952.3027 ± 0.30967	969.96767 ± 0.05071	991.3219 ± 0.10077	
w	32.61389 ± 0	28.5836 ± 0	18.57484 ± 4.55277	13.28699 ± 0.23786	11.70689 ± 0.52327	13.44809 ± 1.39236	11.88768 ± 0.1965	10.77731 ± 0.32865	
A	22.25109 ± 10.61208	23.61788 ± 2.94947	18.28643 ± 3.6805	183.92124 ± 2.51781	106.5761 ± 5.83736	71.04133 ± 8.25384	286.4442 ± 4.71452	107.3875 ± 2.69079	
Reduced Chi-Sqr	0.05626								
R-Square (COD)	0.98987								
Adj. R-Square	0.98928								

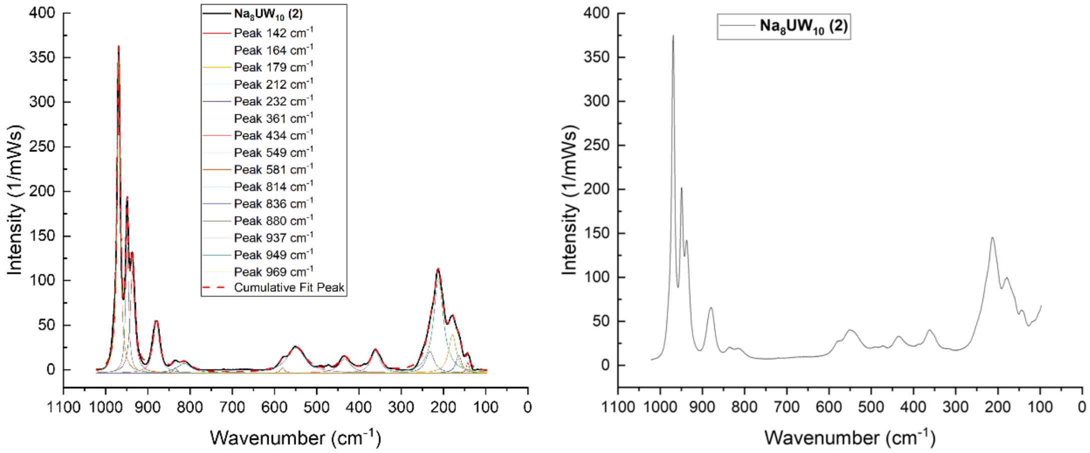

Figure S32. (**Left**) Baseline corrected Raman spectrum of complex **2** ($Na_8[UW_{10}]$) fit to Lorentzian functions. (**Right**) Raw Raman spectrum for complex **2**.

Table S16. Peak fitting parameters for Raman spectrum of complex **2**.

Model	Lorentz								
Equation	y = y0 + (2*A/pi)*(w/(4*(x-xc)^2 + w^2))								
Plot	Peak1	Peak2	Peak3	Peak4	Peak5	Peak6	Peak7	Peak8	Peak9
y0	-3.75311 ± 0.21508	-3.75311 ± 0.21508	-3.75311 ± 0.21508	-3.75311 ± 0.21508	-3.75311 ± 0.21508	-3.75311 ± 0.21508	-3.75311 ± 0.21508	-3.75311 ± 0.21508	-3.75311 ± 0.21508
xc	142.36012 ± 0.49662	163.61932 ± 0.87676	178.57192 ± 0.60092	211.74931 ± 0.25914	232.23883 ± 1.00028	360.87908 ± 0.42134	433.75047 ± 0.71858	549.25158 ± 0.72809	581.24471 ± 1.10054
w	8.07934 ± 1.68636	16.31815 ± 3.07962	21.79064 ± 2.21923	28.00391 ± 1.10274	24.58488 ± 2.63162	26.78169 ± 1.34852	34.37447 ± 2.3451	52.41457 ± 2.24688	11.57855 ± 4.33455
A	150.04905 ± 27.39224	518.44685 ± 159.1996	1481.00679 ± 224.70557	4636.48565 ± 264.83262	939.68636 ± 188.09932	1015.43 ± 40.90637	867.69124 ± 47.10476	2340.6347 ± 99.70106	110.64771 ± 41.7836
Plot	Peak10	Peak11	Peak12	Peak13	Peak14	Peak15			
y0	-3.75311 ± 0.21508	-3.75311 ± 0.21508	-3.75311 ± 0.21508	-3.75311 ± 0.21508	-3.75311 ± 0.21508	-3.75311 ± 0.21508			
xc	814.04737 ± 2.29047	835.55345 ± 1.04434	879.97259 ± 0.13499	936.59401 ± 0.07799	949.22379 ± 0.03915	969.45428 ± 0.01567			
w	42.21205 ± 5.59459	10.28703 ± 4.64307	17.70342 ± 0.4297	12.82337 ± 0.24166	8.09136 ± 0.14293	8.93595 ± 0.04902			
A	714.58399 ± 107.505	98.98247 ± 52.00172	1630.96009 ± 31.80244	2310.59836 ± 41.85404	1998.27703 ± 35.19138	5003.0142 ± 21.1			
Reduced Chi-Sqr	5.48423								
R-Square (COD)	0.99701								
Adj. R-Square	0.99685								

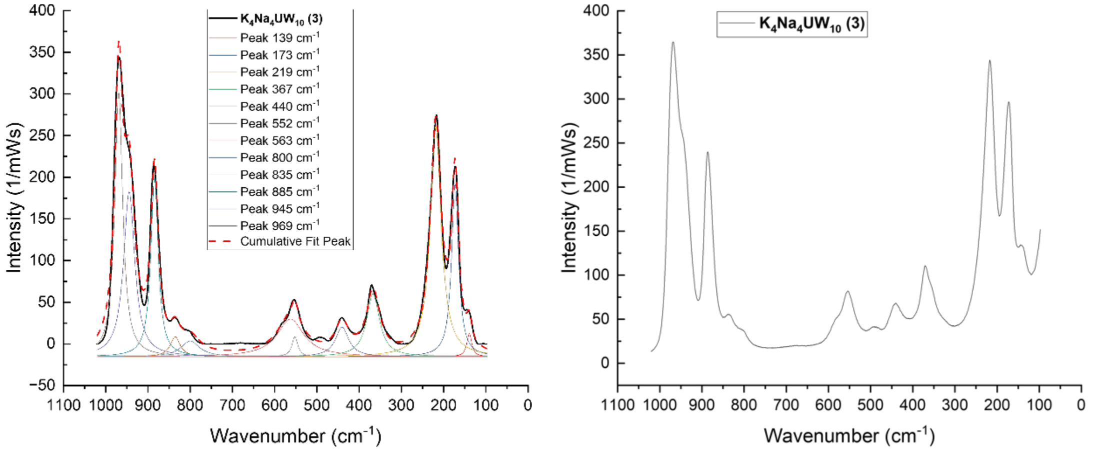

Figure S33. (**Left**) Baseline corrected Raman spectrum of complex **3** ($K_4Na_4[UW_{10}]$) fit to Lorentzian functions. (**Right**) Raw Raman spectrum for complex **3**.

Table S17. Peak fitting parameters for Raman spectrum of complex **3**.

Model	Lorentz								
Equation	y = y0 + (2*A/pi)*(w/(4*(x-xc)^2 + w^2))								
Plot	Peak1	Peak2	Peak3	Peak4	Peak5	Peak6	Peak7	Peak8	Peak9
y0	-15.3661<br>± 0.75438	-15.3661<br>± 0.75438	-15.3661<br>± 0.75438	-15.3661<br>± 0.75438	-15.3661<br>± 0.75438	-15.3661<br>± 0.75438	-15.3661<br>± 0.75438	-15.3661<br>± 0.75438	-15.3661<br>± 0.75438
xc	139.17047<br>± 0.90635	172.90046<br>± 0.15042	218.74623<br>± 0.14097	367.0597<br>± 0.53949	440.48289<br>± 1.15987	552.17764<br>± 1.15961	562.70698<br>± 1.97503	799.95254<br>± 3.29541	835.49158<br>± 1.63661
w	13.84668<br>± 2.92603	19.94841<br>± 0.53703	31.01106<br>± 0.47481	36.83333<br>± 1.8077	37.82702<br>± 3.98172	16.65381<br>± 5.48815	76.95319<br>± 0	53.05317<br>± 0	28.06435<br>± 0
A	603.65395<br>± 102.87065	6488.73521<br>± 145.55843	13455.7844<br>± 174.42729	4361.23699<br>± 180.00091	2112.48436<br>± 184.35169	629.52403<br>± 256.74946	5462.22184<br>± 451.85594	1574.14737<br>± 160.33478	1057.15964<br>± 118.74687
Plot	Peak10	Peak11	Peak12						
y0	-15.3661<br>± 0.75438	-15.3661<br>± 0.75438	-15.3661<br>± 0.75438						
xc	884.5595<br>± 0.13599	944.5177<br>± 0.34894	969.31551<br>± 0.14625						
w	21.05596<br>± 0.46002	31.78353<br>± 1.01048	22.68573<br>± 0.47737						
A	7088.28279<br>± 127.66231	9876.71794<br>± 387.72774	11303.5721<br>± 321.82122						
Reduced Chi-Sqr	60.86186								
R-Square (COD)	0.9901								
Adj. R-Square	0.98971								

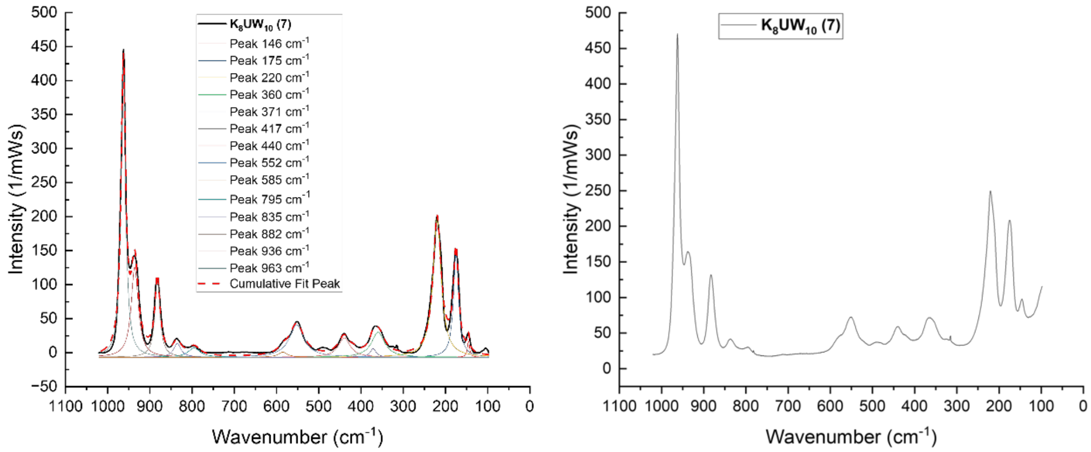

Figure S34. (**Left**) Baseline corrected Raman spectrum of complex **7** ($K_8[UW_{10}]$) fit to Lorentzian functions. (**Right**) Raw Raman spectrum for complex **7**.

Table S18. Peak fitting parameters for Raman spectrum of complex **7**.

Model	Lorentz								
Equation	y = y0 + (2*A/pi)*(w/(4*(x-xc)^2 + w^2))								
Plot	Peak1	Peak2	Peak3	Peak4	Peak5	Peak6	Peak7	Peak8	Peak9
y0	-6.55798 ± 0.43336	-6.55798 ± 0.43336	-6.55798 ± 0.43336	-6.55798 ± 0.43336	-6.55798 ± 0.43336	-6.55798 ± 0.43336	-6.55798 ± 0.43336	-6.55798 ± 0.43336	-6.55798 ± 0.43336
xc	145.54038 ± 0.48256	175.00851 ± 0.11626	219.93801 ± 0.10887	359.7222 ± 4.46561	371.35082 ± 3.036	417.20196 ± 5.17395	439.72774 ± 1.60892	552.40775 ± 0.83775	585.25472 ± 3.40187
w	6.15046 ± 1.43878	14.72092 ± 0.37144	23.43617 ± 0.34702	37.29441 ± 3.38352	16.73651 ± 16.25598	12.58778 ± 20.97959	33.79735 ± 4.45256	42.42351 ± 2.68748	25.24283 ± 0
A	225.32426 ± 39.08537	3499.67379 ± 67.70607	7501.77677 ± 86.89498	2149.58804 ± 798.29483	331.51641 ± 673.89323	69.63132 ± 132.25059	1508.4339 ± 209.75623	3160.88503 ± 192.0621	298.7322 ± 94.65523
Plot	Peak10	Peak11	Peak12	Peak13	Peak14				
y0	-6.55798 ± 0.43336	-6.55798 ± 0.43336	-6.55798 ± 0.43336	-6.55798 ± 0.43336	-6.55798 ± 0.43336				
xc	795.28423 ± 2.02948	835.20229 ± 1.02539	882.16428 ± 0.14465	935.91141 ± 0.15768	962.94094 ± 0.03689				
w	33.80942 ± 0	21.20422 ± 3.49521	14.67356 ± 0.45398	20.32892 ± 0.53398	12.79474 ± 0.12257				
A	728.22235 ± 71.84948	667.64738 ± 89.46736	2615.48961 ± 62.40051	4235.01766 ± 91.93587	8638.38814 ± 70.35548				
Reduced Chi-Sqr	28.7583								
R-Square (COD)	0.99163								
Adj. R-Square	0.99124								

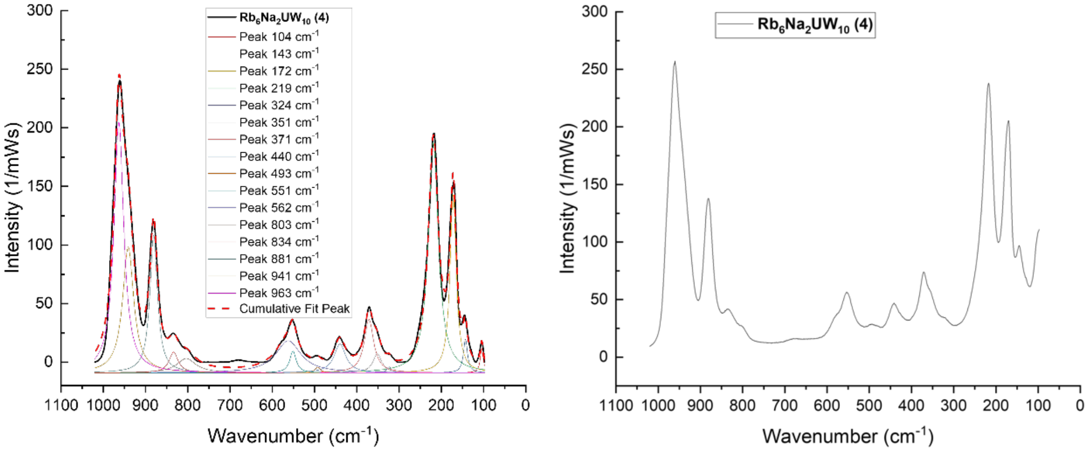

Figure S35. (**Left**) Baseline corrected Raman spectrum of complex **4** ($Rb_6Na_2[UW_{10}]$) fit to Lorentzian functions. (**Right**) Raw Raman spectrum for complex **4**.

Table S19. Peak fitting parameters for Raman spectrum of complex **4**.

Model	Lorentz								
Equation	y = y0 + (2*A/pi)*(w/(4*(x-xc)^2 + w^2))								
Plot	Peak1	Peak2	Peak3	Peak4	Peak5	Peak6	Peak7	Peak8	Peak9
y0	-9.0907<br>± 0.50011	-9.0907<br>± 0.50011	-9.0907<br>± 0.50011	-9.0907<br>± 0.50011	-9.0907<br>± 0.50011	-9.0907<br>± 0.50011	-9.0907<br>± 0.50011	-9.0907<br>± 0.50011	-9.0907<br>± 0.50011
xc	104.42367<br>± 0.612	142.96203<br>± 0.50404	172.05848<br>± 0.12057	218.93144<br>± 0.11685	323.62183<br>± 3.44884	351.49023<br>± 3.06756	371.32608<br>± 1.27401	440.02105<br>± 1.00125	492.69682<br>± 3.5149
w	8.83659<br>± 1.95106	11.5256<br>± 1.63019	17.70441<br>± 0.41824	28.56313<br>± 0.3896	13.85144<br>± 12.73862	25.00528<br>± 11.51089	26.99916<br>± 3.08428	33.31383<br>± 3.53699	15.82746<br>± 12.37777
A	287.169<br>± 46.97191	519.27134<br>± 58.75743	4229.60442<br>± 81.28077	8814.38079<br>± 100.11126	107.89938<br>± 102.16572	637.15753<br>± 433.58182	1933.66667<br>± 389.05371	1284.38995<br>± 112.99538	117.25003<br>± 80.39199
Plot	Peak10	Peak11	Peak12	Peak13	Peak14	Peak15	Peak16		
y0	-9.0907<br>± 0.50011	-9.0907<br>± 0.50011	-9.0907<br>± 0.50011	-9.0907<br>± 0.50011	-9.0907<br>± 0.50011	-9.0907<br>± 0.50011	-9.0907<br>± 0.50011		
xc	551.18787<br>± 1.03733	562.35073<br>± 2.98911	803.48357<br>± 3.78107	833.63526<br>± 1.39855	880.81057<br>± 0.15891	941.14235<br>± 0.55576	963.22066<br>± 0.22728		
w	18.55151<br>± 6.0251	71.86552<br>± 0	54.94777<br>± 0	26.08608<br>± 0	21.51934<br>± 0.54533	31.75257<br>± 1.36397	27.81882<br>± 0.58563		
A	537.68844<br>± 256.22379	3087.18442<br>± 406.47551	1051.39623<br>± 119.78252	722.38175<br>± 86.82234	3974.26051<br>± 84.16177	5351.19416<br>± 370.48052	9353.29604<br>± 342.94982		
Reduced Chi-Sqr	24.22525								
R-Square (COD)	0.99148								
Adj. R-Square	0.99102								

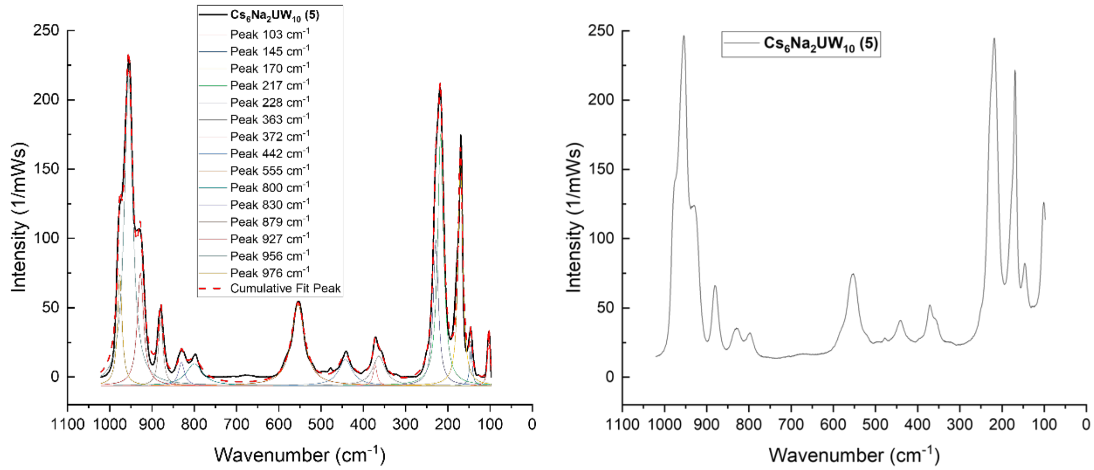

Figure S36. (**Left**) Baseline corrected Raman spectrum of complex **5** ($Cs_{5.5}Na_{2.5}[UW_{10}]$) fit to Lorentzian functions. (**Right**) Raw Raman spectrum for complex **5**.

Table S20. Peak fitting parameters for Raman spectrum of complex **5**.

Model	Lorentz								
Equation	y = y0 + (2*A/pi)*(w/(4*(x-xc)^2 + w^2))								
Plot	Peak1	Peak2	Peak3	Peak4	Peak5	Peak6	Peak7	Peak8	Peak9
y0	-6.21734 ± 0.42674	-6.21734 ± 0.42674	-6.21734 ± 0.42674	-6.21734 ± 0.42674	-6.21734 ± 0.42674	-6.21734 ± 0.42674	-6.21734 ± 0.42674	-6.21734 ± 0.42674	-6.21734 ± 0.42674
xc	102.91641 ± 0.26195	145.28602 ± 0.3306	170.28403 ± 0.08276	217.1323 ± 0.23291	228.45947 ± 0.33439	363.1481 ± 3.34903	372.3014 ± 1.0633	442.49282 ± 1.26504	554.50685 ± 0.38218
w	6.83133 ± 0.82104	6.7548 ± 1.00303	12.56428 ± 0.26344	16.65114 ± 0.51002	14.76183 ± 0.82301	35.90526 ± 4.23571	10.91322 ± 6.11074	38.5491 ± 4.27696	37.35665 ± 1.27282
A	412.94378 ± 36.40384	316.37931 ± 35.36299	3229.66826 ± 51.99581	4750.56164 ± 252.40609	2440.722 ± 241.04483	1193.92546 ± 291.81763	257.55009 ± 228.92526	1151.19545 ± 106.99542	3436.88793 ± 98.3884
Plot	Peak10	Peak11	Peak12	Peak13	Peak14	Peak15			
y0	-6.21734 ± 0.42674	-6.21734 ± 0.42674	-6.21734 ± 0.42674	-6.21734 ± 0.42674	-6.21734 ± 0.42674	-6.21734 ± 0.42674			
xc	799.82105 ± 2.79383	830.46751 ± 1.00945	879.43996 ± 0.25722	927.4633 ± 0.21893	955.66965 ± 0.11006	976.41457 ± 0.1913			
w	47.05134 ± 7.87061	17.15681 ± 4.04102	12.60142 ± 0.78935	17.98305 ± 0.77439	23.36366 ± 0.51049	12.87212 ± 0.72012			
A	1175.6085 ± 215.60142	451.76267 ± 122.87013	971.12786 ± 46.29821	2296.71557 ± 100.96912	8281.48691 ± 188.59196	1617.7927 ± 104.11942			
Reduced Chi-Sqr	19.97141								
R-Square (COD)	0.99142								
Adj. R-Square	0.99096								

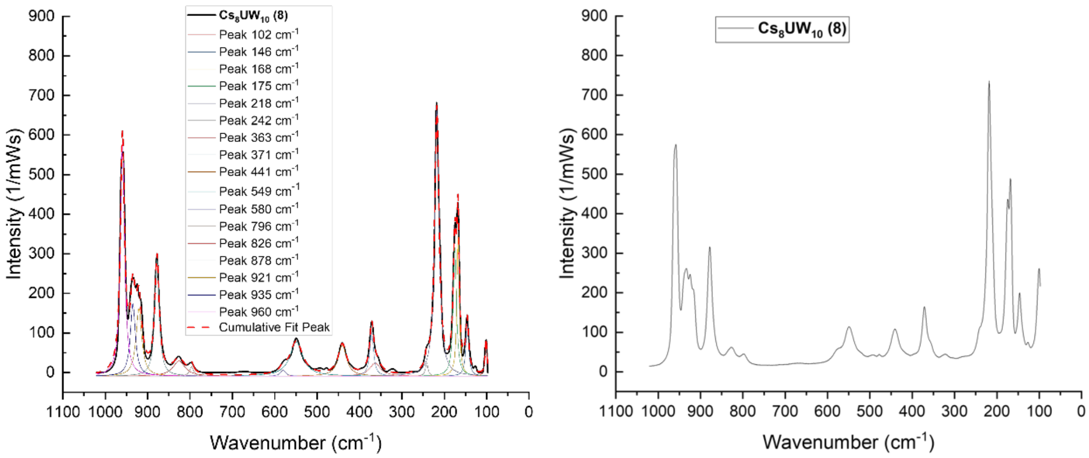

Figure S37. (**Left**) Baseline corrected Raman spectrum of complex **8** ($Cs_8[UW_{10}]$) fit to Lorentzian functions. (**Right**) Raw Raman spectrum for complex **8**.

Table S21. Peak fitting parameters for Raman spectrum of complex **8**.

Model	Lorentz								
Equation	y = y0 + (2*A/pi)*(w/(4*(x-xc)^2 + w^2))								
Plot	Peak1	Peak2	Peak3	Peak4	Peak5	Peak6	Peak7	Peak8	Peak9
y0	-9.12818 ± 0.79917	-9.12818 ± 0.79917	-9.12818 ± 0.79917	-9.12818 ± 0.79917	-9.12818 ± 0.79917	-9.12818 ± 0.79917	-9.12818 ± 0.79917	-9.12818 ± 0.79917	-9.12818 ± 0.79917
xc	101.6805 ± 0.22849	145.9709 ± 0.19687	167.5809 ± 0.09591	175.10362 ± 0.14922	217.51665 ± 0.04813	241.53758 ± 0.76384	363.32697 ± 5.41702	371.43662 ± 0.39911	440.61015 ± 0.56594
w	5.24062 ± 0.70736	8.05075 ± 0.59429	6.54041 ± 0.31925	9.00359 ± 0.40038	12.39667 ± 0.15112	7.40222 ± 2.32922	30.9095 ± 5.97163	10.86374 ± 2.26999	23.73303 ± 1.77817
A	771.27846 ± 74.97517	1682.67184 ± 92.23288	3654.64775 ± 216.80336	4589.50073 ± 242.08932	13197.7533 ± 121.32862	379.767 ± 90.07323	1618.52722 ± 788.71755	1872.01153 ± 675.57758	2904.90837 ± 168.96106
Plot	Peak10	Peak11	Peak12	Peak13	Peak14	Peak15	Peak16	Peak17	
y0	-9.12818 ± 0.79917	-9.12818 ± 0.79917	-9.12818 ± 0.79917	-9.12818 ± 0.79917	-9.12818 ± 0.79917	-9.12818 ± 0.79917	-9.12818 ± 0.79917	-9.12818 ± 0.79917	
xc	549.28553 ± 0.69537	580.463 ± 2.18568	796.35643 ± 1.30731	826.15018 ± 1.4453	877.96555 ± 0.10342	920.64271 ± 0.37251	934.92355 ± 0.28815	959.44057 ± 0.05101	
w	36.00709 ± 2.32483	13.69963 ± 0	11.83214 ± 0	33.91635 ± 5.16972	12.19458 ± 0.32829	16.30166 ± 1.04255	14.17611 ± 1.02579	11.53496 ± 0.1672	
A	4888.84813 ± 264.40909	335.06998 ± 100.36343	437.07461 ± 89.99488	2067.04027 ± 260.49513	5627.10431 ± 118.21667	4366.34646 ± 357.96136	4093.95774 ± 365.34949	10799.6086 ± 130.24787	
Reduced Chi-Sqr	118.05234								
R-Square (COD)	0.98989								
Adj. R-Square	0.9893								

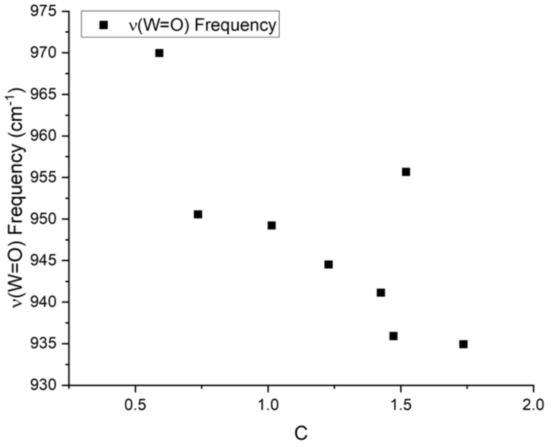

Figure S38. Plot of effective ionic radius vs. Raman ν(W=O) mode frequencies.

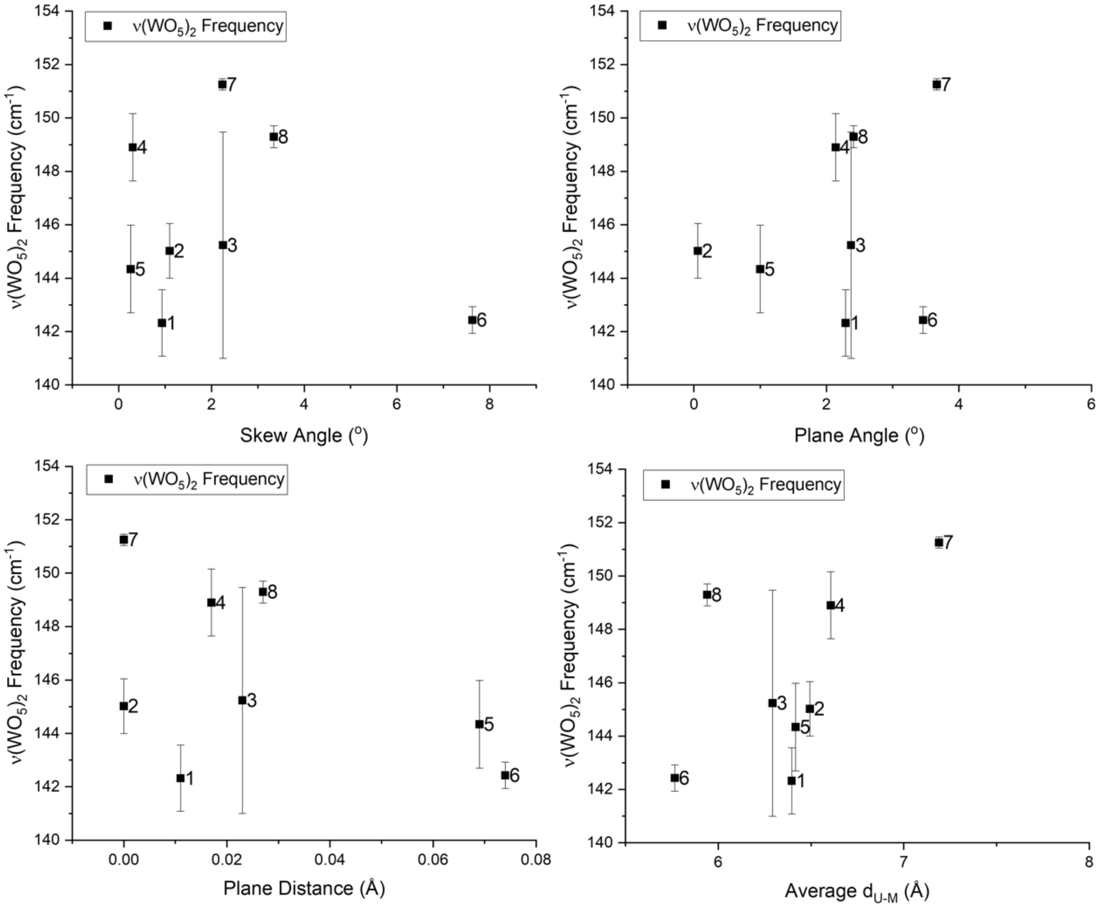

Figure S39. Plots of FIR $\nu(WO_5)_2$ frequencies vs. distortion and structural parameters for complexes **1**-**8**. (**Top Left**) Comparison of $\nu(WO_5)_2$ frequencies vs. skew angles. (**Top Right**) Comparison of $\nu(WO_5)_2$ frequencies vs. plane angles. (**Bottom Left**) Comparison of $\nu(WO_5)_2$ frequencies vs. plane distances. (**Bottom Right**) Comparison of $\nu(WO_5)_2$ frequencies vs. average $d_{U\text{-}M}$ distances. The $\nu(WO_5)_2$ frequencies were obtained from the fitted FIR spectra of complexes **1**-**8**. Error bars represent uncertainties of the peak center obtained from the fitting regime.

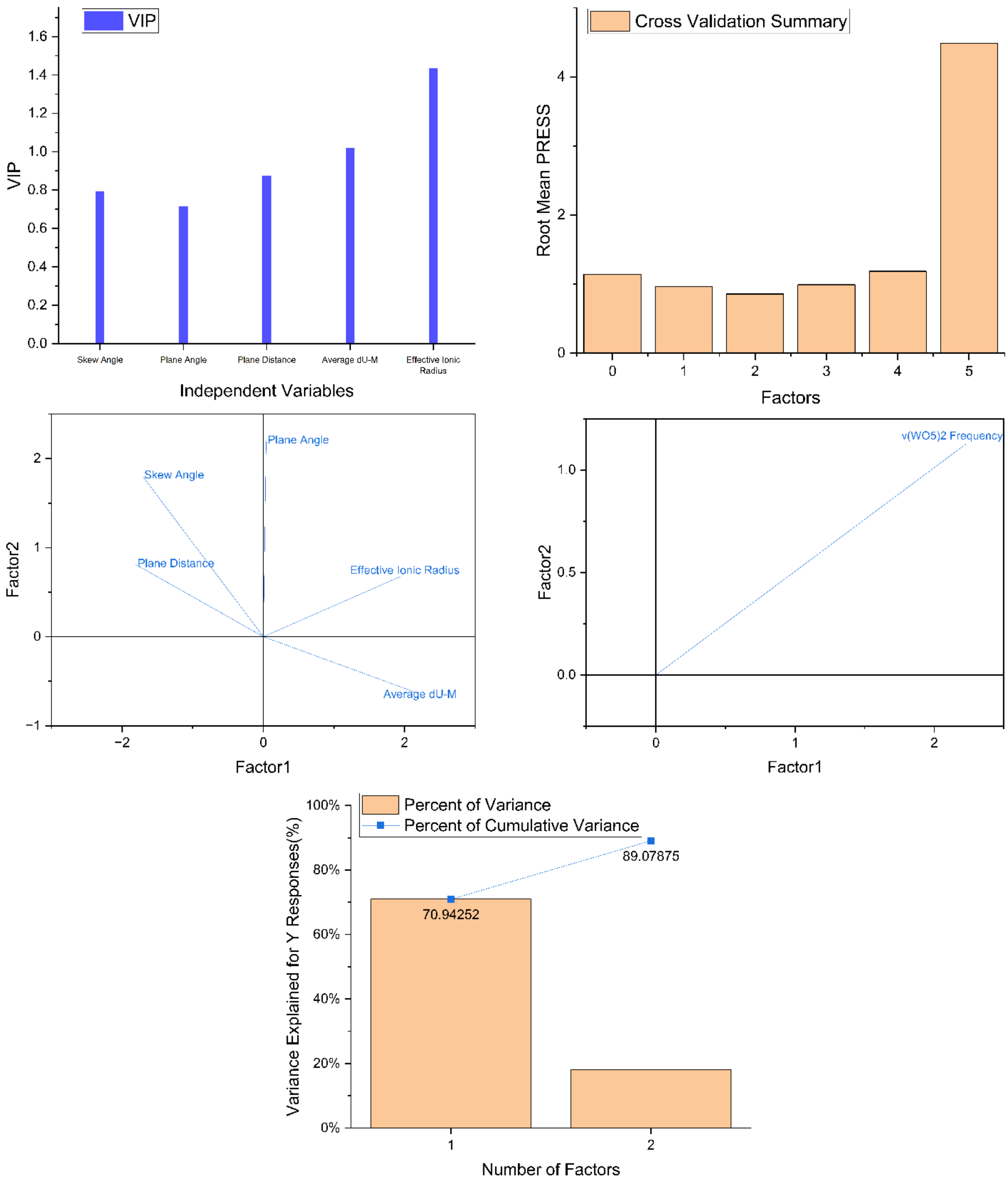

Figure S40. (**Top Left**) VIP plot, (**Top Right**) RMS plot, (**Middle Left**) X-loading plot, (**Middle Right**) Y-loading plot, and (**Bottom**) Y-variance accountability plot for PLS analysis comparing distortion and structural parameters (SA, PA, PD, counterion eIR, and average $d_{U\text{-}M}$ distance) and $\nu(WO_5)_2$ frequencies. FIR frequencies were obtained from the fitted spectra of complexes **1-8**.

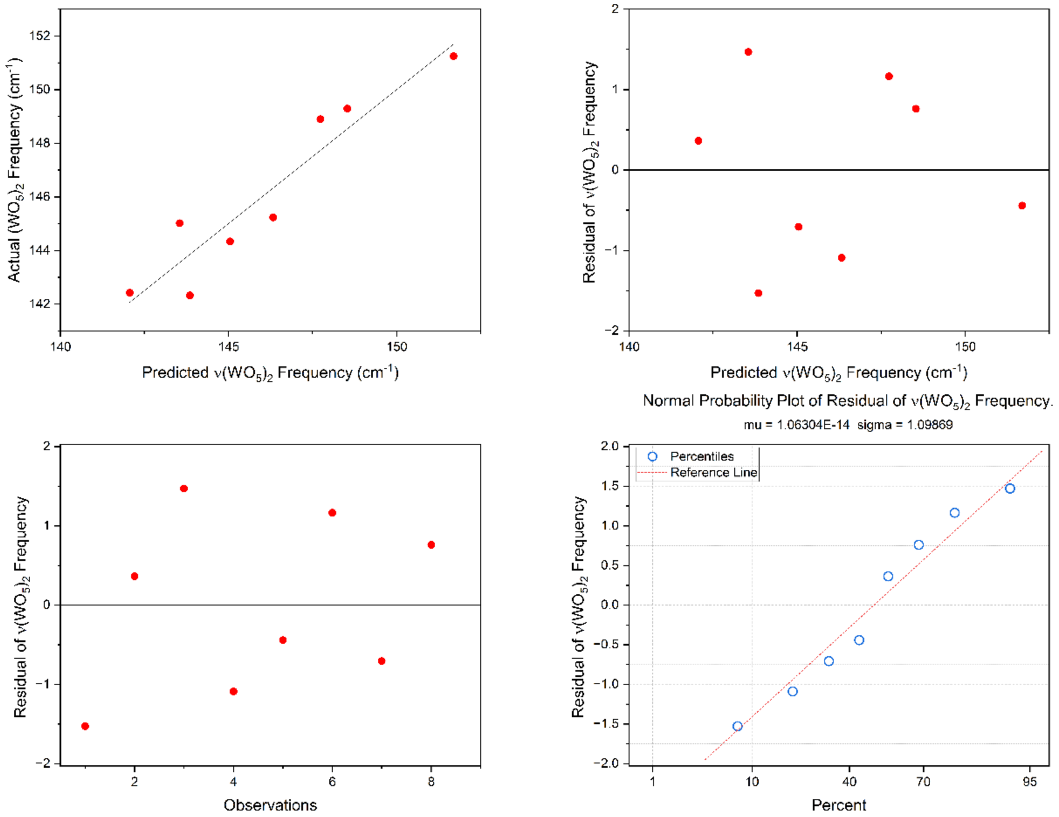

Figure S41. Diagnostic plots from the PLS analysis comparing distortion and structural parameters (SA, PA, PD, counterion eIR, and average $d_{U\text{-}M}$ distance) and FIR $\nu(WO_5)_2$ frequencies.

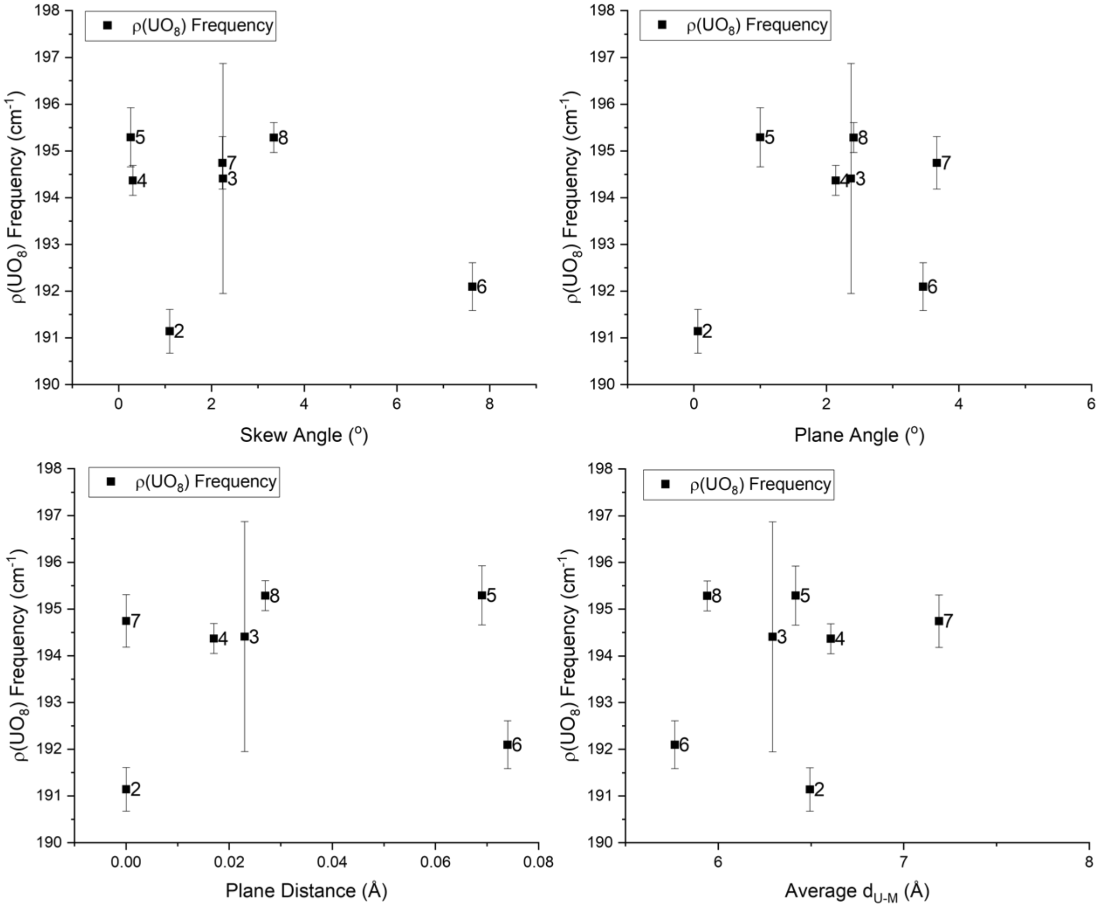

Figure S42. Plots of FIR $\rho(UO_8)$ frequencies vs. distortion and structural parameters for complexes **2-8** (this stretching mode was not observed in the spectrum for **1**). (**Top Left**) Comparison of $\rho(UO_8)$ frequencies vs. skew angles. (**Top Right**) Comparison of $\rho(UO_8)$ frequencies vs. plane angles. (**Bottom Left**) Comparison of $\rho(UO_8)_2$ frequencies vs. plane distances. (**Bottom Right**) Comparison of $\rho(UO_8)$ frequencies vs. average $d_{U\text{-}M}$ distances. The $\rho(UO_8)$ frequencies were obtained from the fitted FIR spectra of complexes **2-8**. Error bars represent uncertainties of the peak center obtained from the fitting regime.

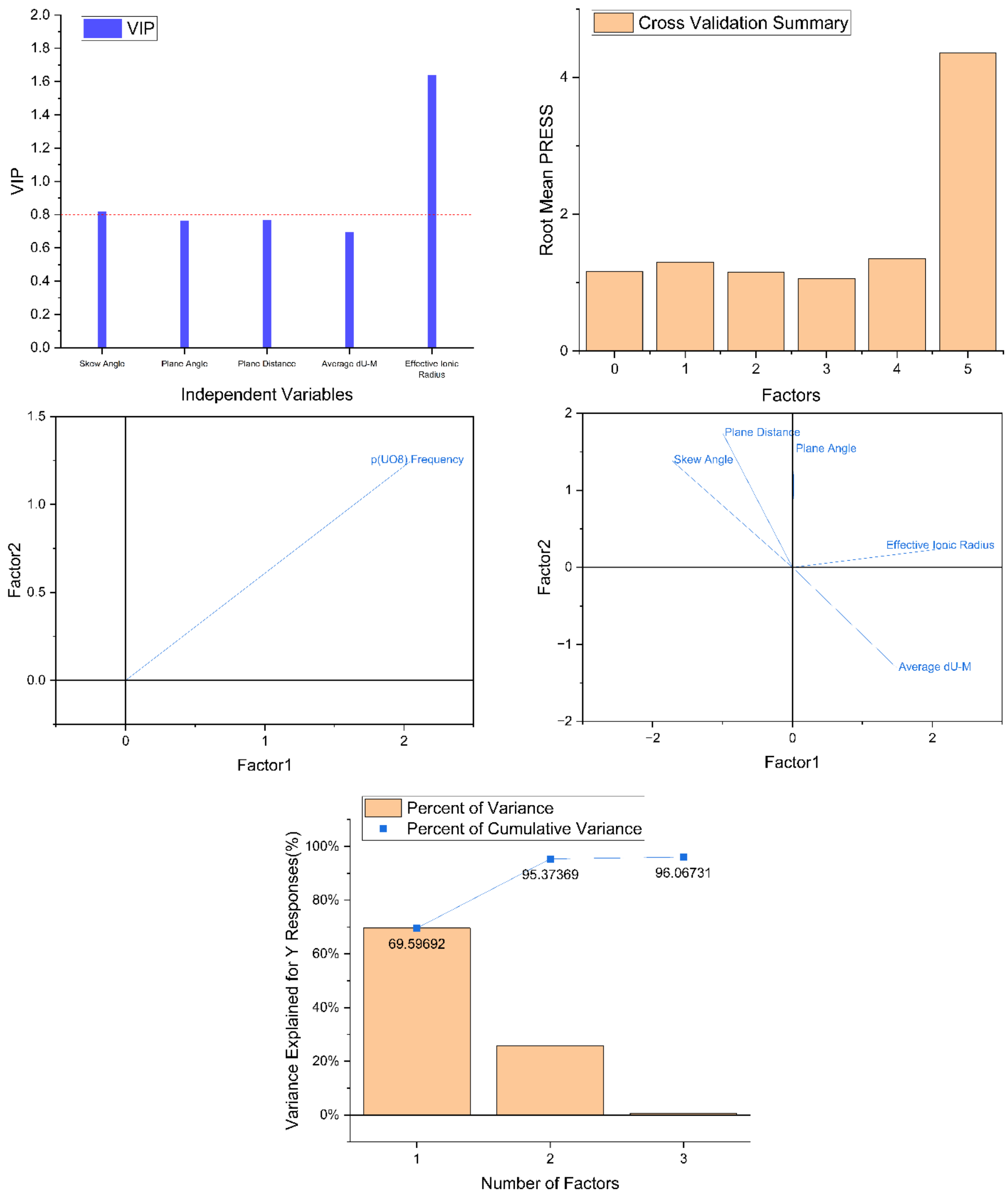

Figure S43. (**Top Left**) VIP plot, (**Top Right**) RMS plot, (**Middle Left**) X-loading plot, (**Middle Right**) Y-loading plot, and (**Bottom**) Y-variance accountability plot for PLS analysis comparing distortion and structural parameters (SA, PA, PD, counterion eIR, and average $d_{U\text{-}M}$ distance) and ρ(UO_8) frequencies. FIR frequencies were obtained from the fitted spectra of complexes **1-8**.

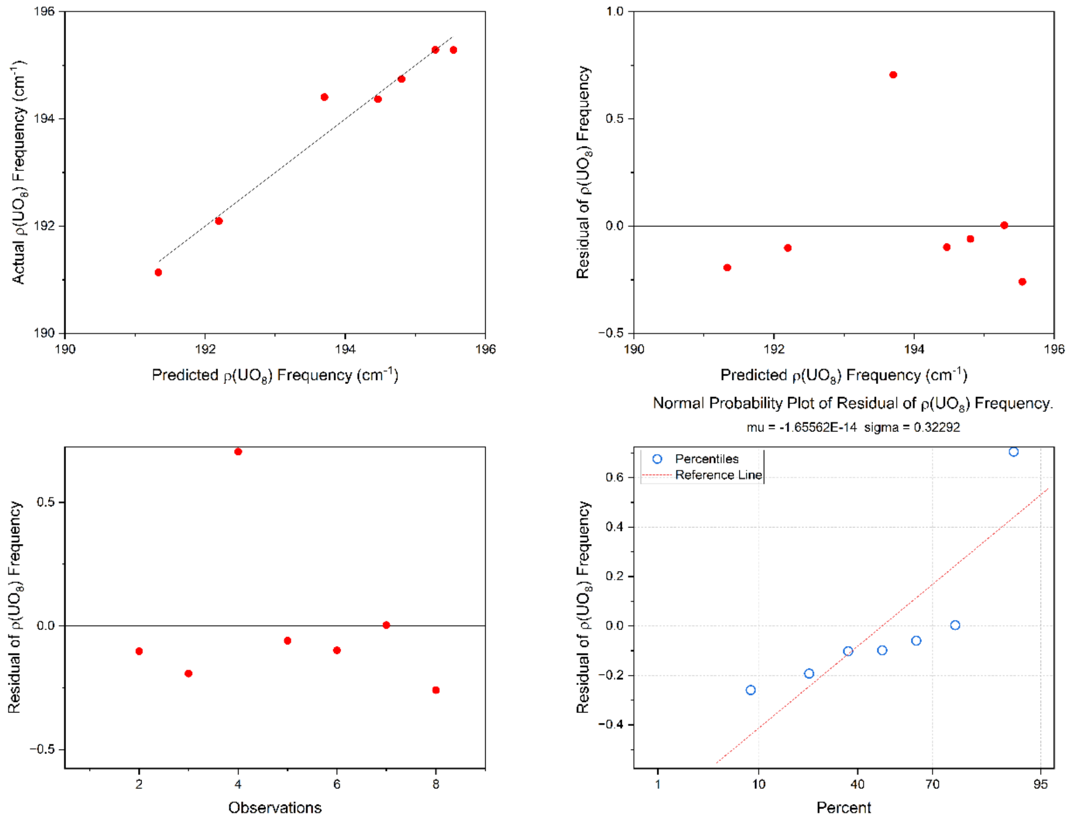

Figure S44. Diagnostic plots from the PLS analysis comparing distortion and structural parameters (SA, PA, PD, counterion eIR, and average $d_{U\text{-}M}$ distance) and FIR $\rho(UO_8)$ frequencies.

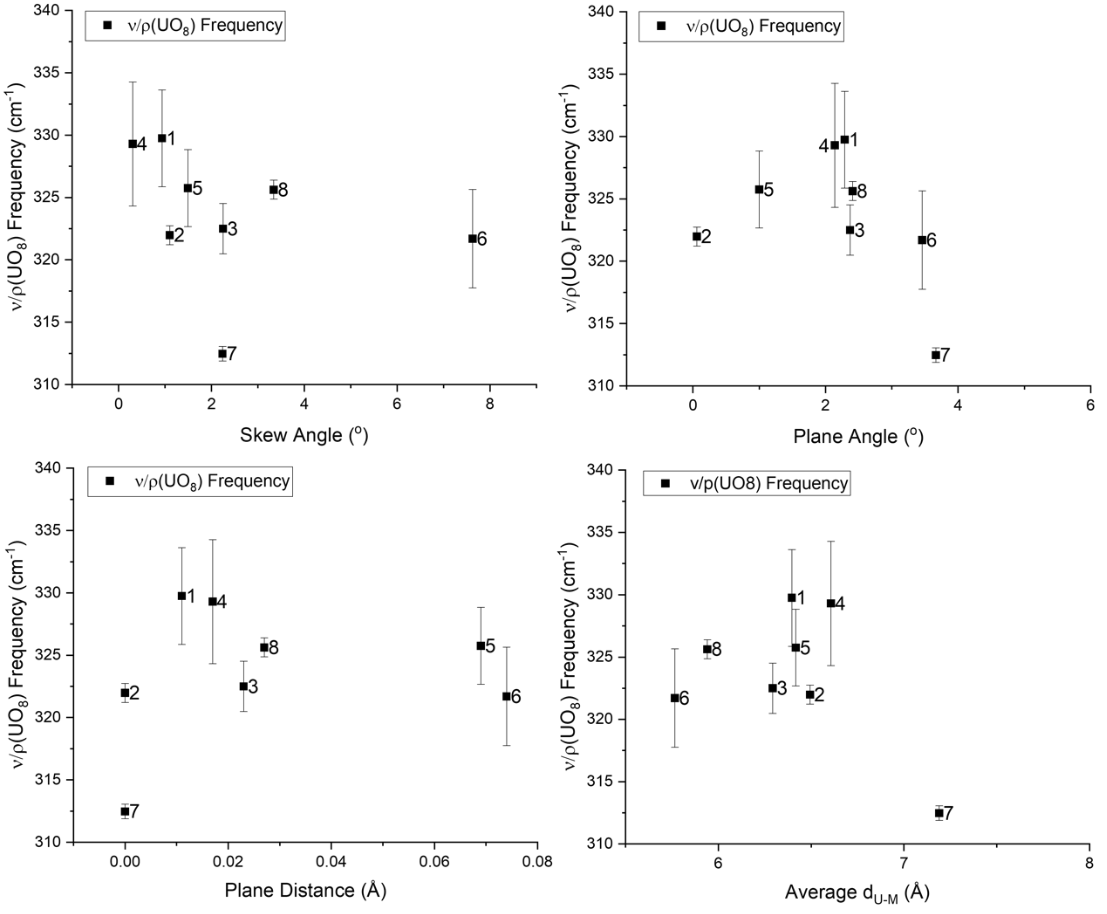

Figure S45. Plots of FIR $\nu/\rho(UO_8)$ frequencies vs. distortion and structural parameters for complexes **1**-**8**. **(Top Left)** Comparison of $\nu/\rho(UO_8)$ frequencies vs. skew angles. (**Top Right**) Comparison of $\nu/\rho(UO_8)$ frequencies vs. plane angles. (**Bottom Left**) Comparison of $\nu/\rho(UO_8)$ frequencies vs. plane distances. (**Bottom Right**) Comparison of $\nu/\rho(UO_8)$ frequencies vs. average $d_{U\text{-}M}$ distances. The $\nu/\rho(UO_8)$ frequencies were obtained from the fitted FIR spectra of complexes **1**-**8**. Error bars represent uncertainties of the peak center obtained from the fitting regime.

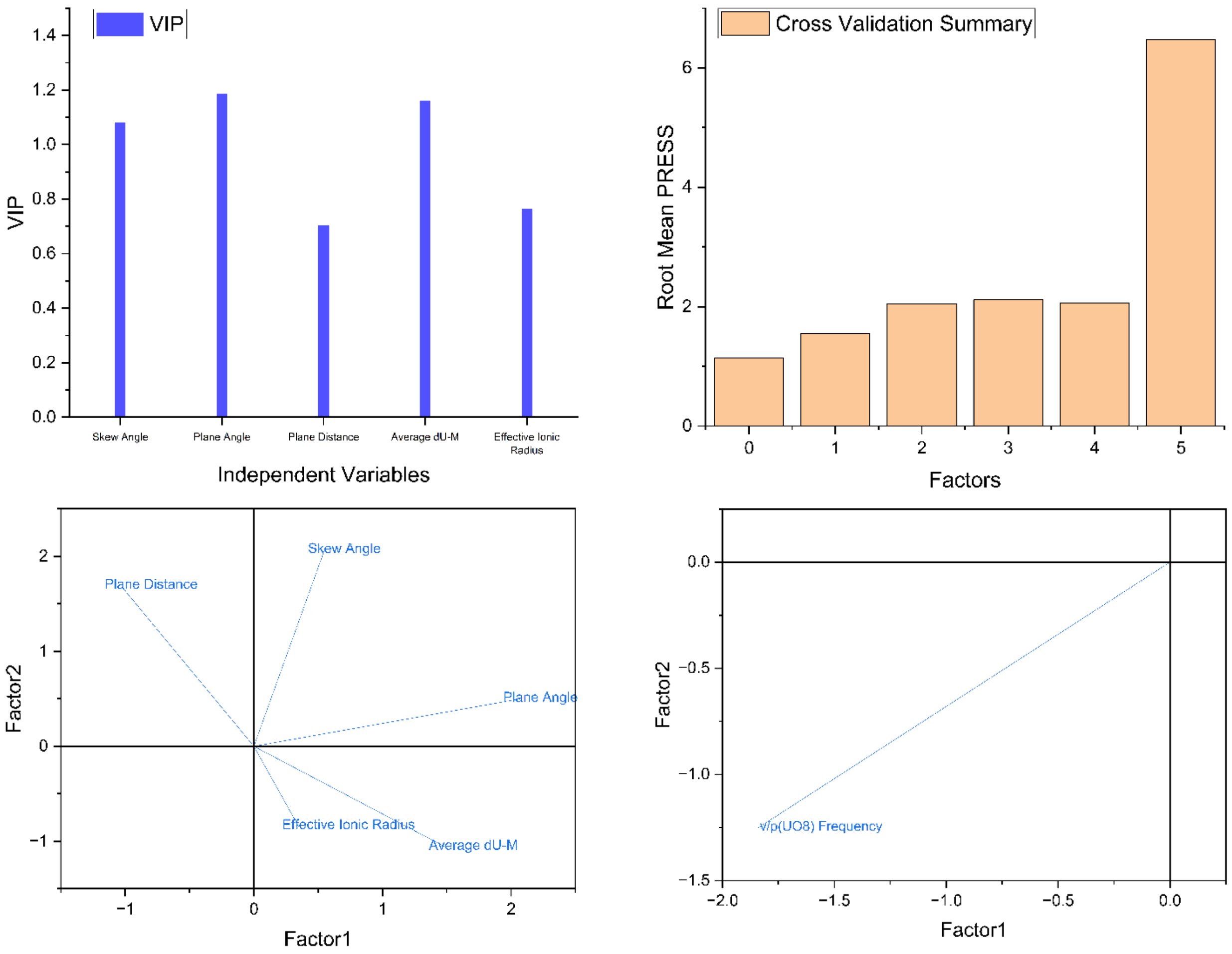

Figure S46. (**Top Left**) VIP plot, **(Top Right**) RMS plot, (**Bottom Left**) X-loading plot, and (**Bottom Right**) Y-loading plot for PLS analysis comparing distortion and structural parameters (SA, PA, PD, counterion eIR, and average $d_{U\text{-}M}$ distance) and ν/ρ(UO_8) frequencies. FIR frequencies were obtained from the fitted spectra of complexes **1-8**. The RMS plot shows that there are no latent variables can be built out of the independent variables. The VIP plot and X- and Y-loading plots were acquired by bypassing the cross-validation test. The loading plots show that plane angle and average $d_{U\text{-}M}$ distance are the most correlated variable with ν/ρ(UO_8) frequencies. FIR frequencies were obtained from the fitted spectra of complexes **1-8**.

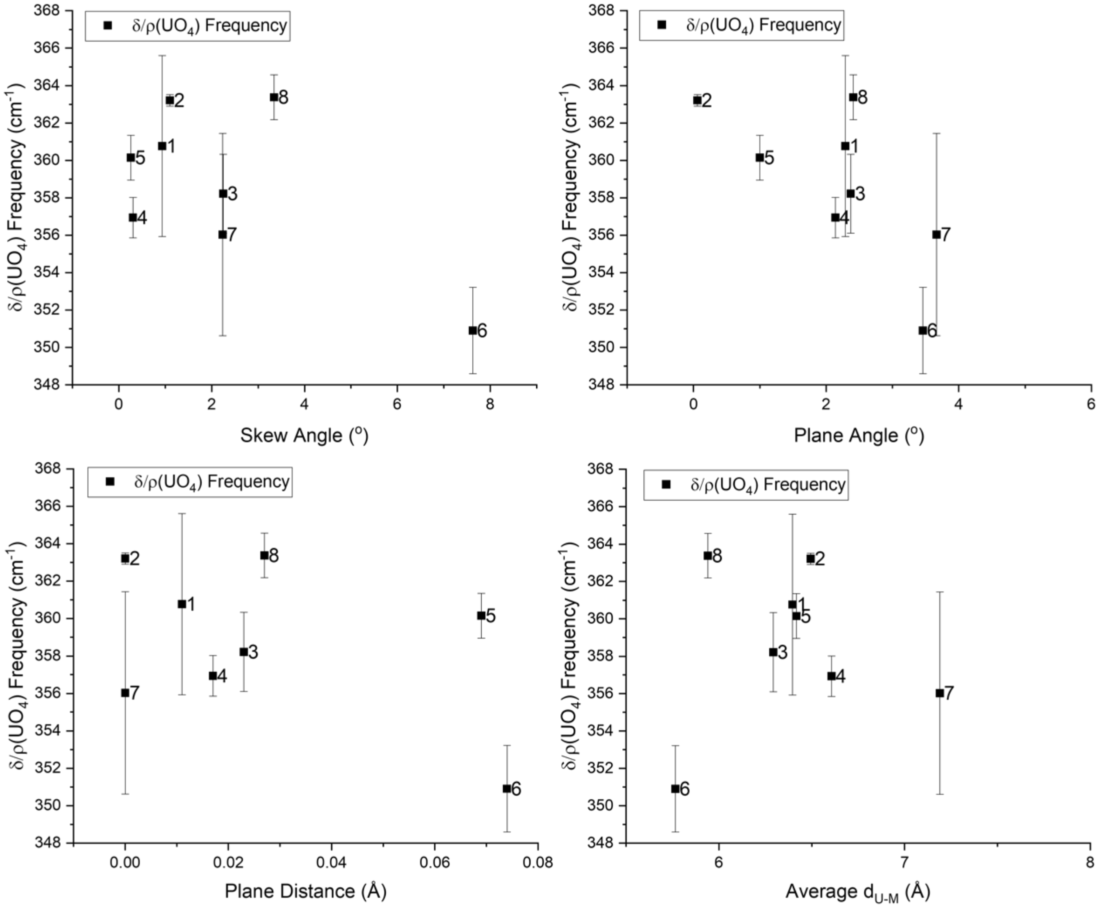

Figure S47. Plots of FIR δ/ρ(UO_4) frequencies vs. distortion and structural parameters for complexes **1-8**. **(Top Left**) Comparison of δ/ρ(UO_4) frequencies vs. skew angles. (**Top Right**) Comparison of δ/ρ(UO_4) frequencies vs. plane angles. (**Bottom Left**) Comparison of δ/ρ(UO_4) frequencies vs. plane distances. (**Bottom Right**) Comparison of δ/ρ(UO_4) frequencies vs. average d_{U-M} distances. The δ/ρ(UO_4) frequencies were obtained from the fitted FIR spectra of complexes **1-8**. Error bars represent uncertainties of the peak center obtained from the fitting regime.

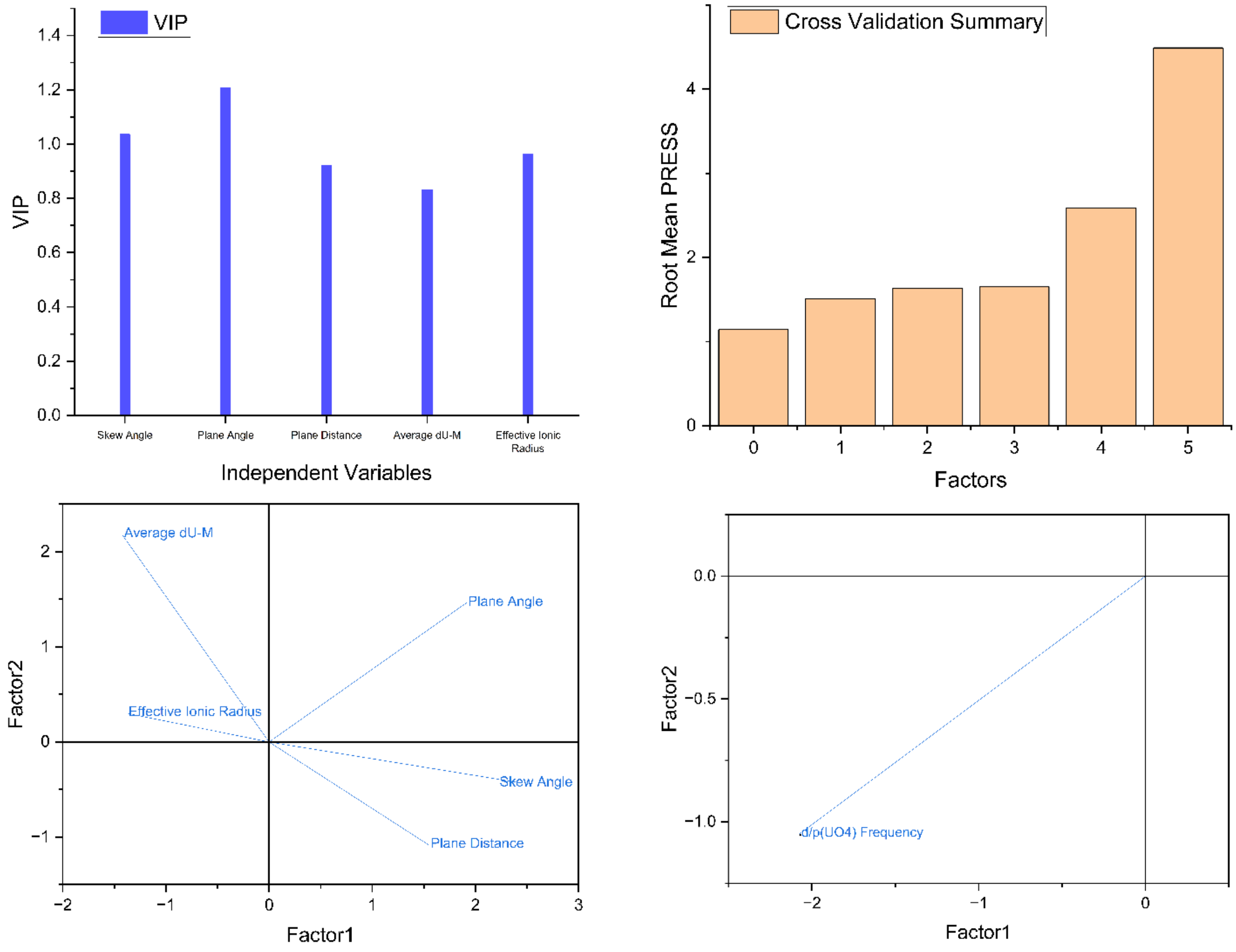

Figure S48. (**Top Left**) VIP plot, (**Top Right**) RMS plot, (**Bottom Left**) X-loading plot, and (**Bottom Right**) Y-loading plot for PLS analysis comparing distortion and structural parameters (SA, PA, PD, counterion eIR, and average $d_{U\text{-}M}$ distance) and $\delta/\rho(UO_4)$ frequencies. FIR frequencies were obtained from the fitted spectra of complexes **1-8**. The RMS plot shows that there are no latent variables can be built out of the independent variables. The VIP plot and X- and Y-loading plots were acquired by bypassing the cross-validation test. The loading plots show that plane angle is the most correlated variable with $\delta/\rho(UO_4)$ frequencies.

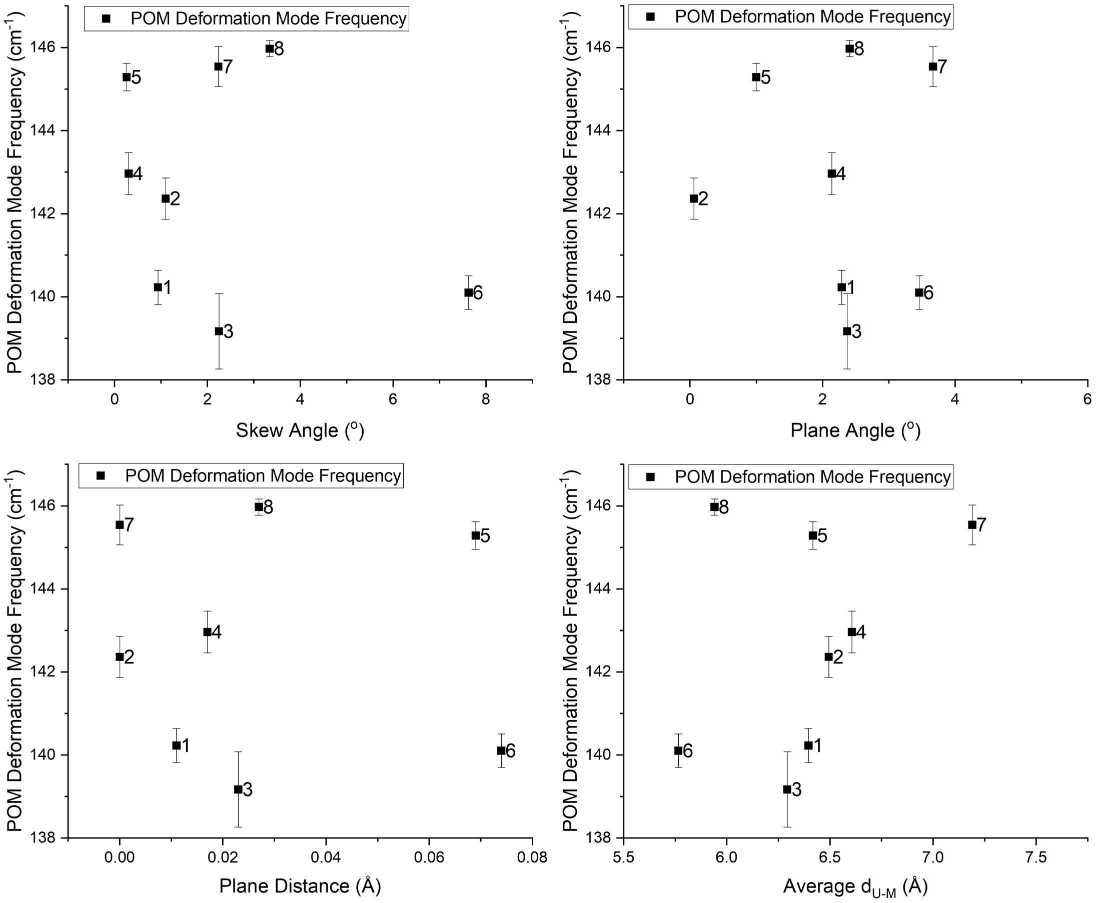

Figure S49. Plots of Raman POM deformation mode frequencies vs. distortion and structural parameters for complexes **1-8**. **(Top Left)** Comparison of POM deformation frequencies vs. skew angles. (**Top Right**) Comparison of POM deformation mode frequencies vs. plane angles. (**Bottom Left**) Comparison of POM deformation mode frequencies vs. plane distances. (**Bottom Right**) Comparison of POM deformation mode frequencies vs. average d_{U-M} distances. The POM deformation mode frequencies were obtained from the fitted Raman spectra of complexes **1-8**. Error bars represent uncertainties of the peak center obtained from the fitting regime.

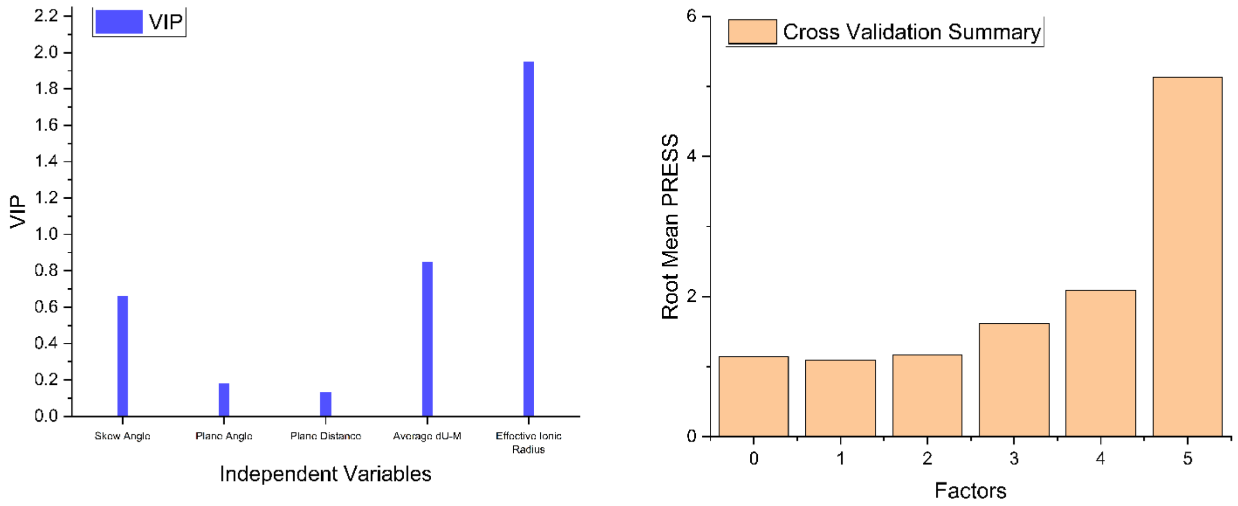

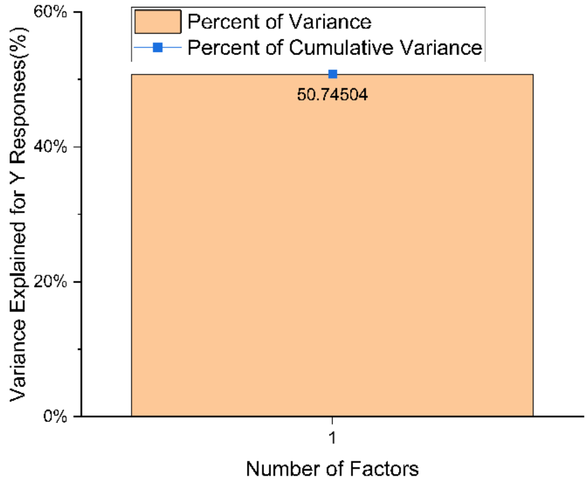

X Variable	Factor1	Y Variable	Factor1
Skew Angle	-2.00498	POM Deformation Mode Frequency	1.88472
Plane Angles	-0.6847		
Plane Distance	-1.20124		
Average dU-M	1.79449		
Effective Ionic Radii	2.32474		

Figure S50. (**Top Left**) VIP plot, (**Top Right**) RMS plot, (**Bottom Left**) Y-variance accountability plot, and (**Bottom Right**) loading table for PLS analysis comparing distortion and structural parameters (SA, PA, PD, counterion eIR, and average $d_{U\text{-}M}$ distance) and POM deformation mode frequencies. Raman frequencies were obtained from the fitted spectra of complexes **1-8**.

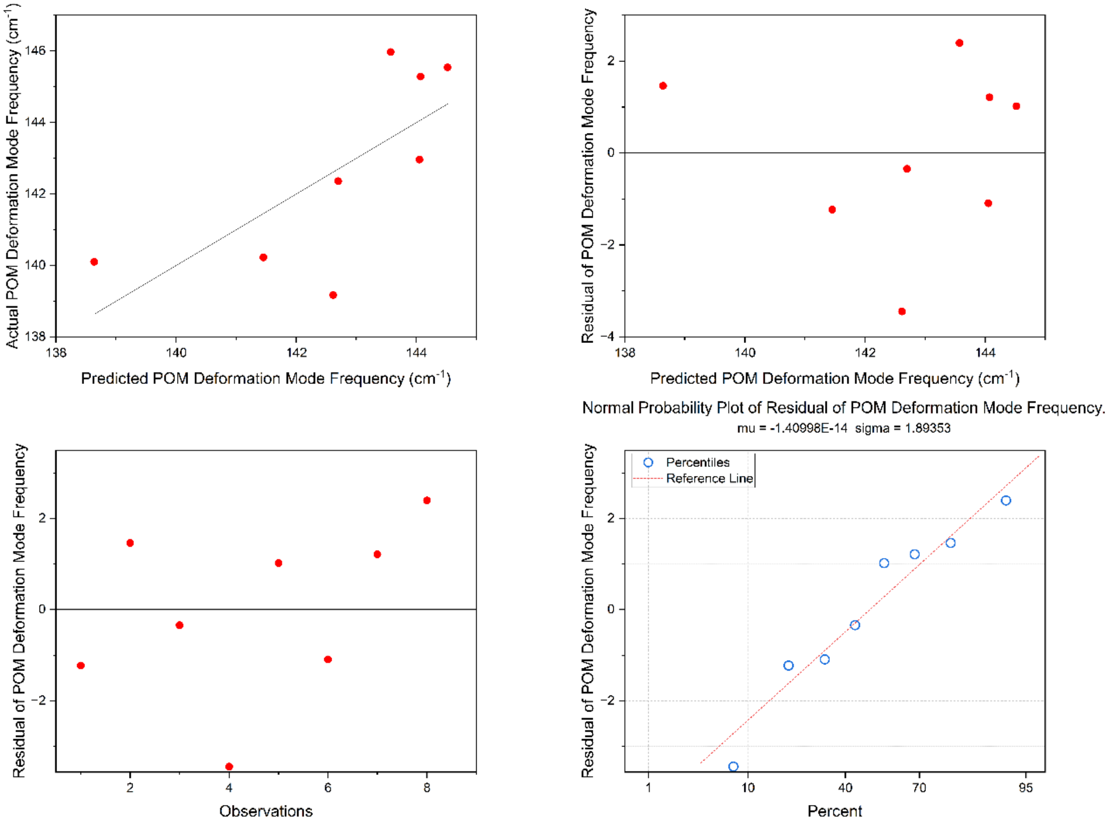

Figure S51. Diagnostic plots from the PLS analysis comparing distortion and structural parameters (SA, PA, PD, counterion eIR, and average $d_{U\text{-}M}$ distance) and Raman POM deformation mode frequencies.

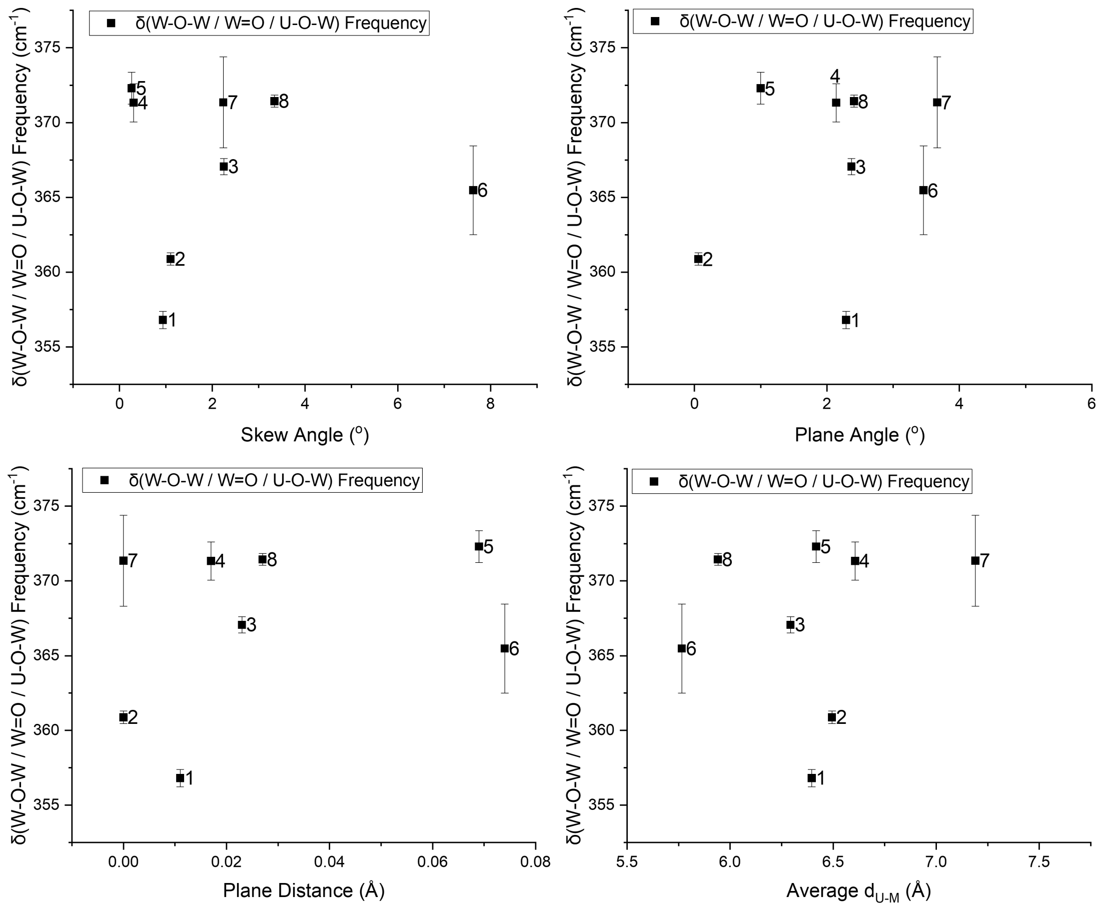

Figure S52. Plots of Raman δ(W-O-W/W=O/U-O-W) frequencies vs. distortion and structural parameters for complexes **1-8**. **(Top Left)** Comparison of δ(W-O-W/W=O/U-O-W) frequencies vs. skew angles. (**Top Right**) Comparison of δ(W-O-W/W=O/U-O-W) frequencies vs. plane angles. (**Bottom Left**) Comparison of δ(W-O-W/W=O/U-O-W) frequencies vs. plane distances. (**Bottom Right**) Comparison of δ(W-O-W/W=O/U-O-W) frequencies vs. average d_{U-M} distances. The δ(W-O-W/W=O/U-O-W) frequencies were obtained from the fitted Raman spectra of complexes **1-8**. Error bars represent uncertainties of the peak center obtained from the fitting regime.

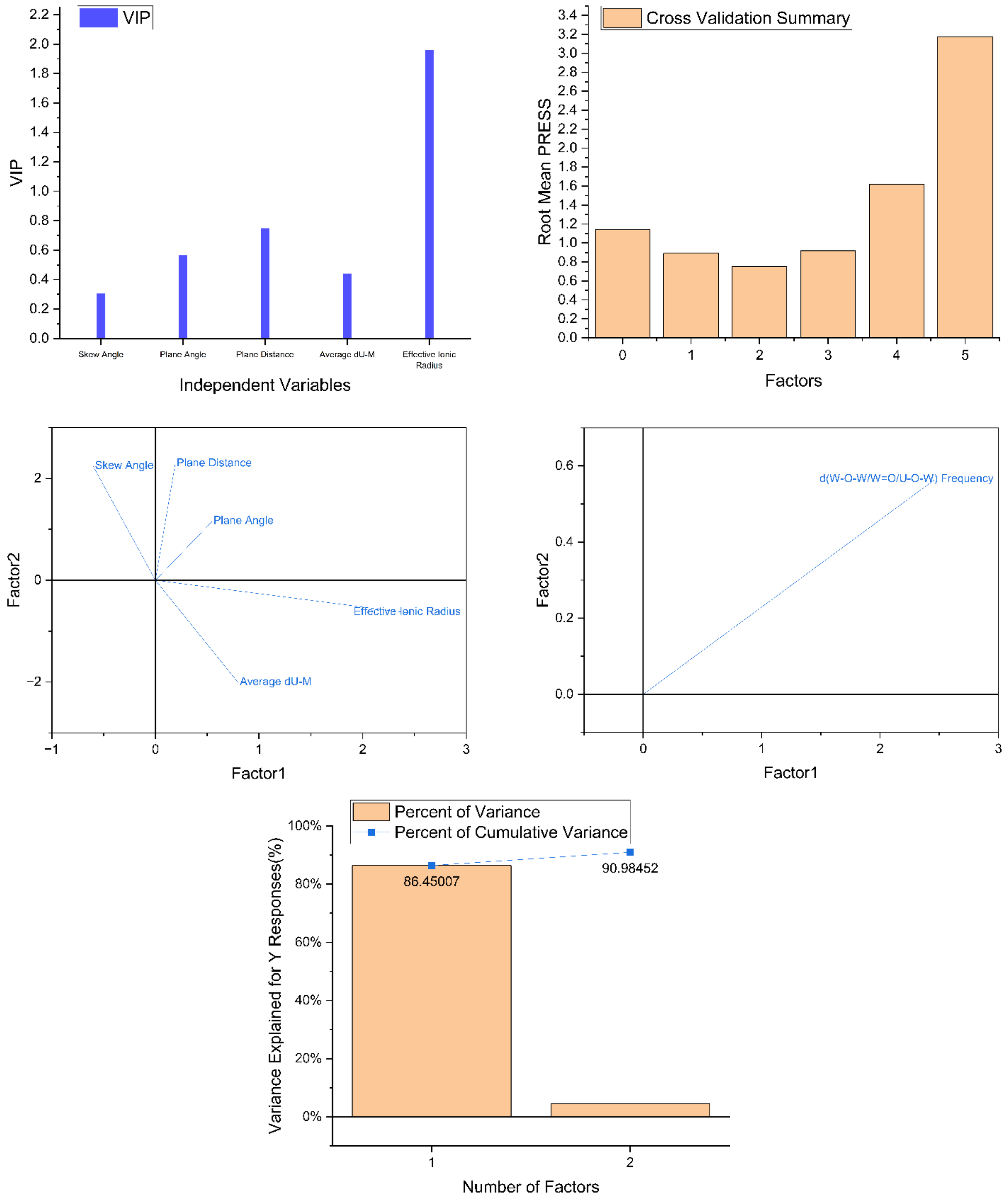

Figure S53. (**Top Left**) VIP plot, (**Top Right**) RMS plot, (**Middle Left**) X-loading plot, (**Middle Right**) Y-loading plot, and (**Bottom**) Y-variance accountability plot for PLS analysis comparing distortion and structural parameters (SA, PA, PD, counterion eIR, and average $d_{U\text{-}M}$ distance) and δ(W-O-W/W=O/U-O-W) frequencies. Raman frequencies were obtained from the fitted spectra of complexes **1-8**.

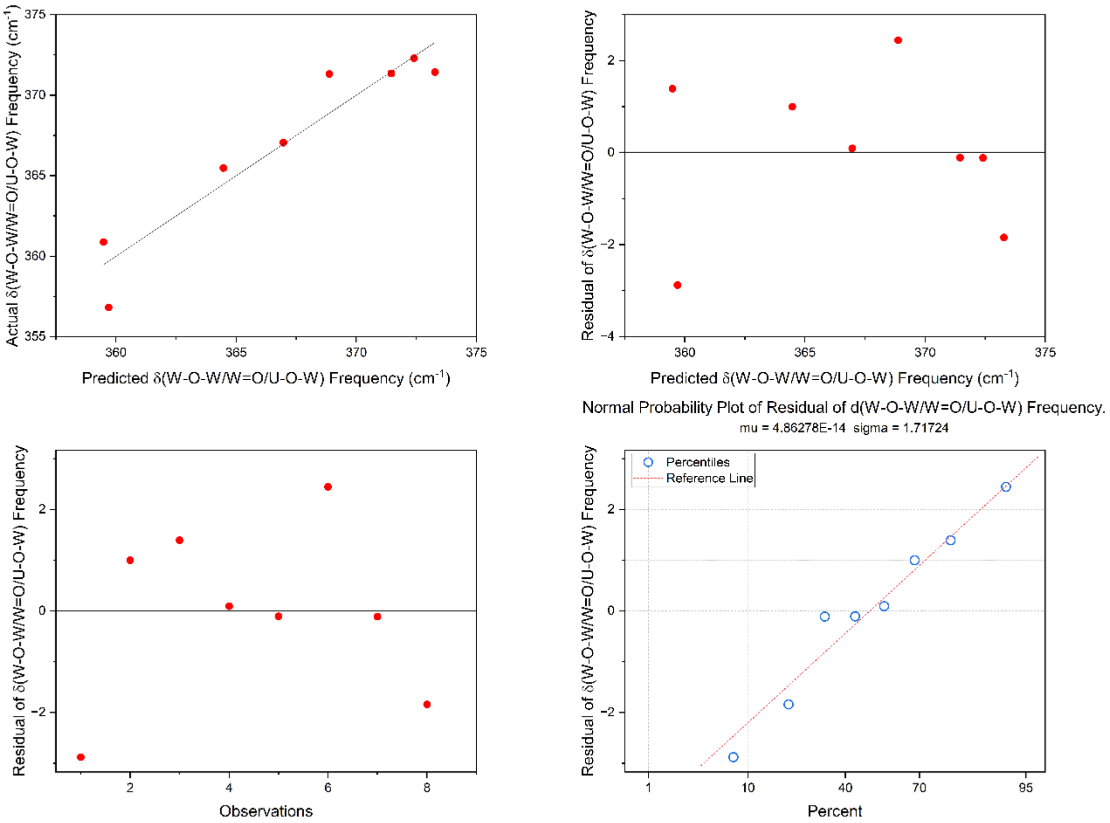

Figure S54. Diagnostic plots from the PLS analysis comparing distortion and structural parameters (SA, PA, PD, counterion eIR, and average $d_{U\text{-}M}$ distance) and Raman δ(W-O-W/W=O/U-O-W) frequencies.

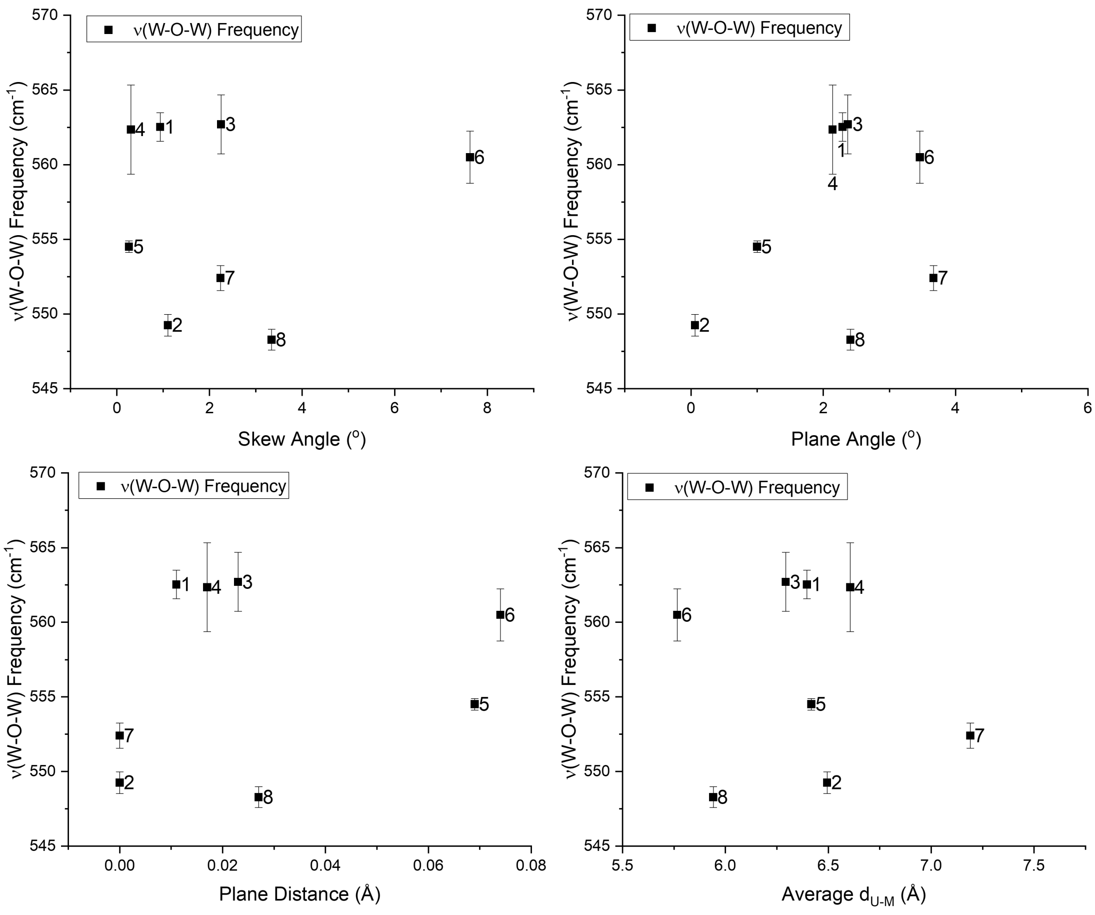

Figure S55. Plots of Raman ν(W-O-W) frequencies vs. distortion and structural parameters for complexes **1-8**. **(Top Left)** Comparison of ν(W-O-W) frequencies vs. skew angles. (**Top Right**) Comparison of ν(W-O-W) frequencies vs. plane angles. (**Bottom Left**) Comparison of ν(W-O-W) frequencies vs. plane distances. (**Bottom Right**) Comparison of ν(W-O-W) frequencies vs. average d_{U-M} distances. The ν(W-O-W) frequencies were obtained from the fitted Raman spectra of complexes **1-8**. Error bars represent uncertainties of the peak center obtained from the fitting regime.

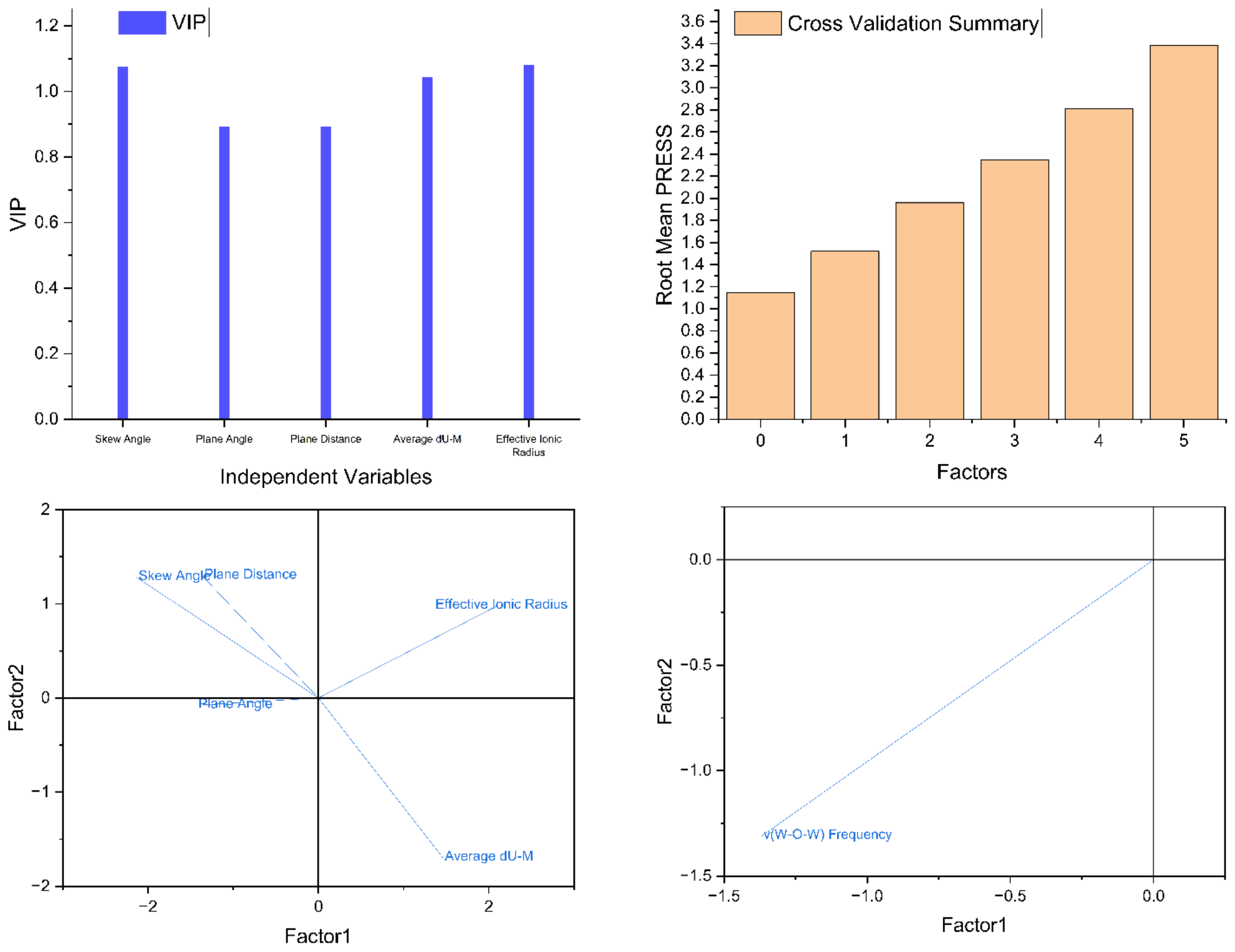

Figure S56. (**Top Left**) VIP plot, **(Top Right**) RMS plot, (**Bottom Left**) X-loading plot, and (**Bottom Right**) Y-loading plot for PLS analysis comparing distortion and structural parameters (SA, PA, PD, counterion eIR, and average d_{U-M} distance) and ν(W-O-W) frequencies. Raman frequencies were obtained from the fitted spectra of complexes **1**-**8**. RMS plot shows that there are no latent variables can be built out of the independent variables. The VIP plot and X- and Y-loading plots were acquired by bypassing the cross-validation test. The loading plots show that SAs and eIR are the two parameters that display the most correlation with the ν(W-O-W) frequencies.

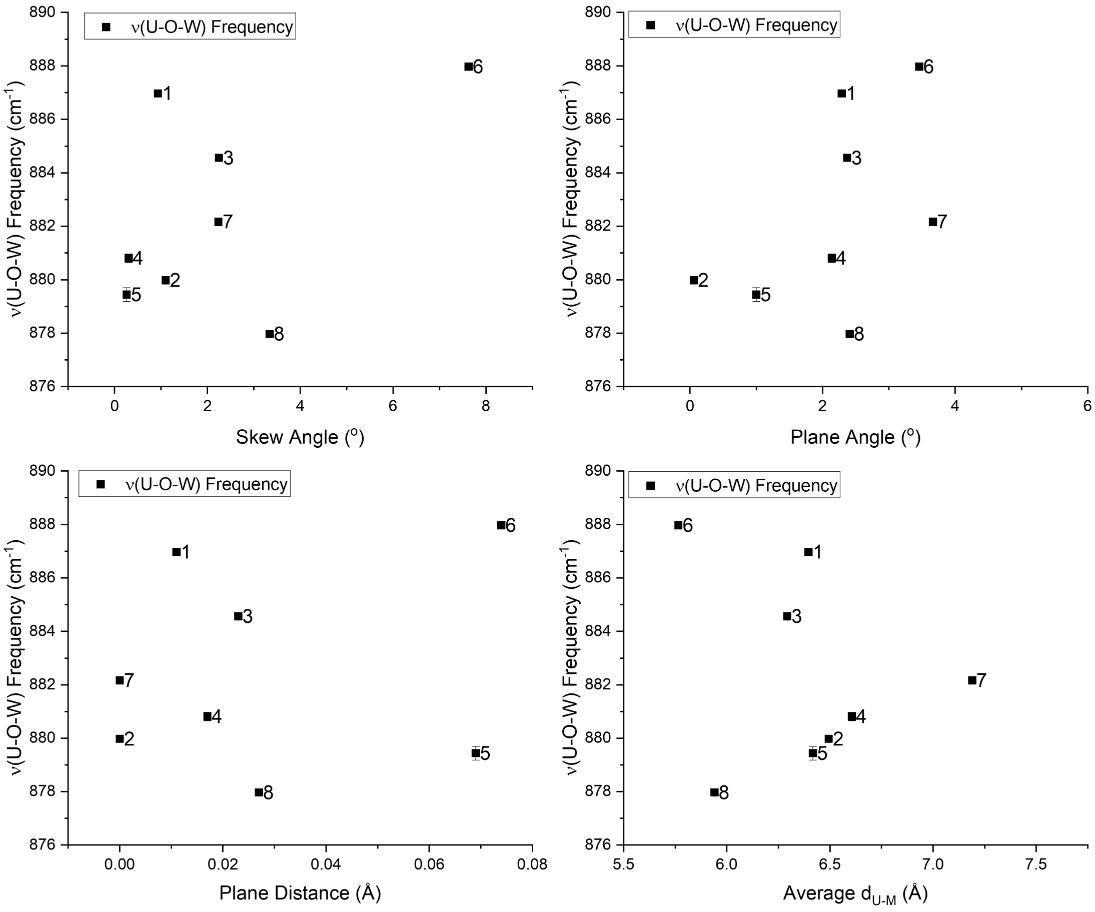

Figure S57. Plots of Raman ν(U-O-W) frequencies vs. distortion and structural parameters for complexes **1-8**. **(Top Left)** Comparison of ν(U-O-W) frequencies vs. skew angles. (**Top Right**) Comparison of ν(U-O-W) frequencies vs. plane angles. (**Bottom Left**) Comparison of ν(U-O-W) frequencies vs. plane distances. (**Bottom Right**) Comparison of ν(U-O-W) frequencies vs. average d_{U-M} distances. The ν(U-O-W) frequencies were obtained from the fitted Raman spectra of complexes **1-8**. Error bars represent uncertainties of the peak center obtained from the fitting regime.

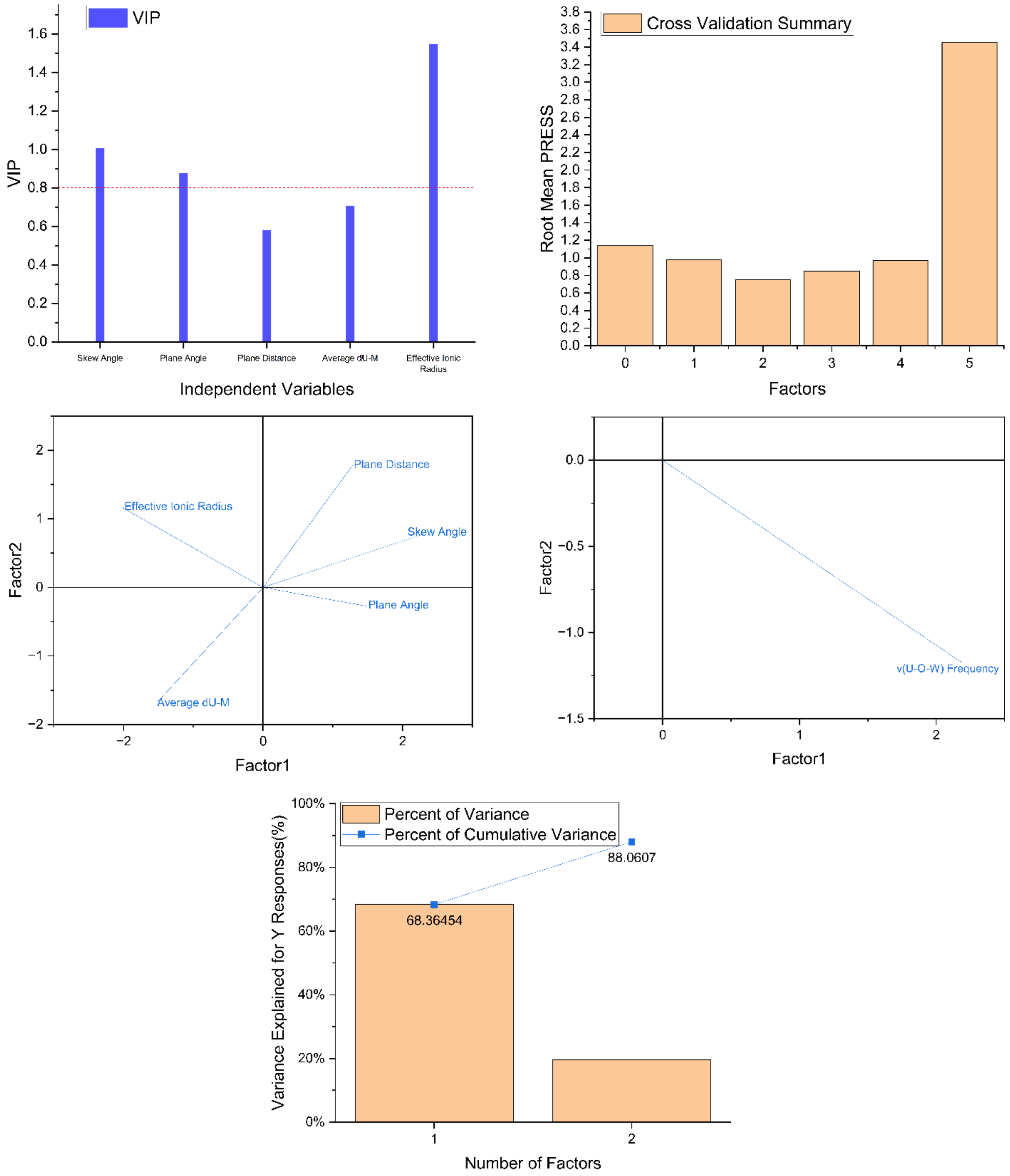

Figure S58. (**Top Left**) VIP plot, (**Top Right**) RMS plot, (**Middle Left**) X-loading plot, (**Middle Right**) Y-loading plot, and (**Bottom**) Y-variance accountability plot for PLS analysis comparing distortion and structural parameters (SA, PA, PD, counterion eIR, and average $d_{U\text{-}M}$ distance) and ν(U-O-W) frequencies. Raman frequencies were obtained from the fitted spectra of complexes **1-8**.

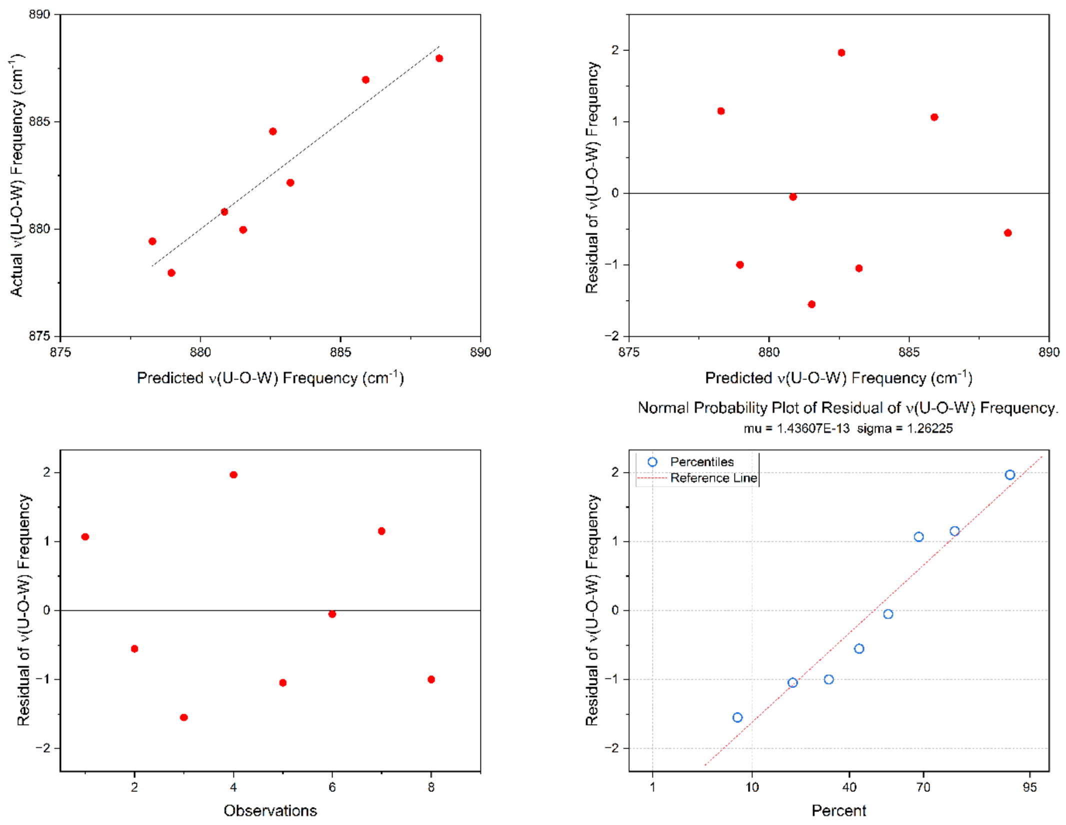

Figure S59. Diagnostic plots from the PLS analysis comparing distortion and structural parameters (SA, PA, PD, counterion eIR, and average $d_{U\text{-}M}$ distance) and Raman ν(U-O-W) frequencies.

References

(1) Shannon, R. D. Revised effective ionic radii and systematic studies of interatomic distances in halides and chalcogenides. *Acta Crystallographica Section A: Crystal Physics, Diffraction, Theoretical and General Crystallography* **1976**, *32*, 751-767.
(2) Geladi, P.; Kowalski, B. R. Partial least-squares regression: a tutorial. *Analytica Chimica Acta* **1986**, *185*, 1-17.
(3) OriginPro, Version 2024, Origin Lab Corporation, Northampton, MA, USA.
(4) Gelfand, A. E.; Dey, D. K.; Chang, H. Model determination using predictive distributions with implementation via sampling-based methods. *Bayesian Statistics* **1992**, *4*, 147-167.
